# Selective Deuteration Reveals the Importance of Multiple Branching Pathways in α-Pinene Autoxidation

**DOI:** 10.1021/jacs.4c14462

**Published:** 2025-04-15

**Authors:** Melissa Meder, Frans Graeffe, Yuanyuan Luo, Jingyi Luo, Siddharth Iyer, Rashid Valiev, Runlong Cai, Matti Rissanen, Theo Kurtén, Jonathan G. Varelas, Franz M. Geiger, Regan J. Thomson, Mikael Ehn

**Affiliations:** 1 Institute for atmospheric and earth system research (INAR/physics), University of Helsinki, Helsinki 00014, Finland; 2 Department of Chemistry, Northwestern University, Evanston, Illinois 60208, United States; 3 Aerosol Physics Laboratory, Tampere University, Tampere 33720, Finland; 4 Department of Chemistry, University of Helsinki, Helsinki 00014, Finland; 5 Shanghai Key Laboratory of Atmospheric Particle Pollution and Prevention (LAP3), Department of Environmental Science & Engineering, Fudan University, Shanghai 200438, China

## Abstract

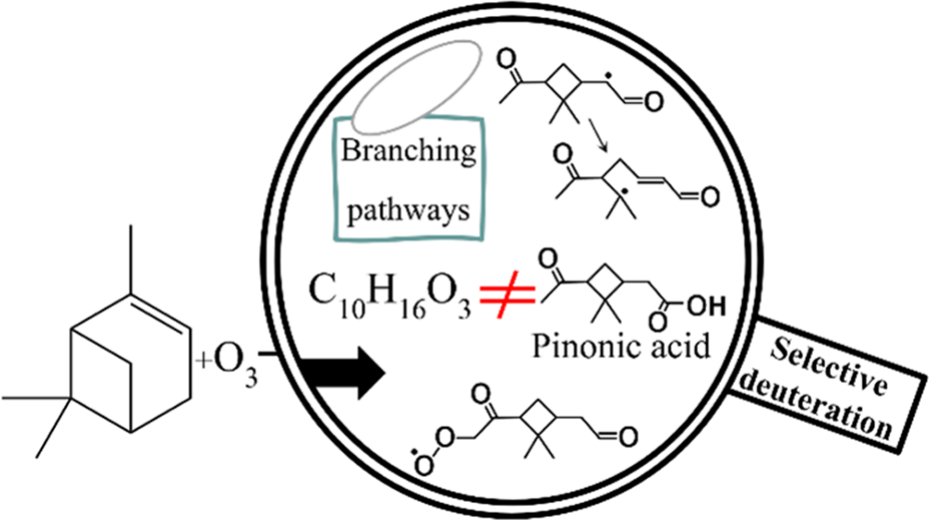

The oxidation of monoterpenes is one of the largest single sources of atmospheric secondary organic aerosol (SOA) significantly impacting the climate and air quality. Still, the autoxidation mechanisms converting these volatile precursors to low-volatility condensable products remain elusive even for the most abundant monoterpene α-pinene. We studied the ozonolysis of α-pinene by combining advanced isotopic labeling and state-of-the-art chemical ionization mass spectrometry supported by quantum chemical calculations. We reacted a full set of eight selectively deuterated α-pinene analogues separately in a flow reactor to probe the oxidation mechanisms on a molecular level. We found that surprisingly few carbon atoms participate in the autoxidation process when forming even the most oxygenated products. Additionally, prompt pinonic acid formation has likely been greatly overestimated, whereas the α-pinene-derived dioxirane appears more stable than previously thought. Importantly, we reveal that oxidation models should include multiple branching pathways rather than simple linear autoxidation progression from less to more oxygenated species. Correct modeling of the oxidation is crucial to enable accurate predictions in the changing climate and atmospheric conditions.

## Introduction

Biogenic volatile organic compounds (VOCs) impact atmospheric reactivity, and their oxidation is the main source of atmospheric organic aerosol.^1,2^ Monoterpenes (C_10_H_16_) constitute around 15 to 20% of the global nonmethane VOC emissions,^3^ wherefore the oxidation chemistry of these species has received broad scientific attention.^4−7^ The larger size of the monoterpene molecules makes them more efficient at forming SOA with higher total SOA production than the most abundant nonmethane VOC isoprene,^3^ making the oxidation of monoterpenes one of the largest, if not the largest, single sources of SOA in the atmosphere.^7^ Additionally, the larger size leads to more potential reaction pathways and increases the number of potential isomeric products.

A major SOA-forming pathway in monoterpene oxidation is autoxidation^8^ where peroxy radical (RO_2_) intermediates undergo hydrogen shift isomerizations (H-shifts) that allow the rapid addition of molecular O_2_.^4^ This process produces low-volatile, reactive, and highly oxygenated peroxides that are challenging to detect. H-shifts are also extremely sensitive to the exact structure of RO_2_,^9−11^ likely contributing to why SOA yields range from 0 to over 80% depending on which monoterpene and which oxidant are reacting under which conditions.^12^ Being the most abundant monoterpene,^3^ the ozonolysis of α-pinene is a significant source of atmospheric SOA. Accordingly, much work has been expended on constructing computational mechanisms^9,10^ and developing models to predict the behavior of this system in the atmosphere.^13−15^ While these have been constructed to match observations, questions remain concerning both the completeness of the mechanisms and mechanistic correctness of the models.

**Figure 1:**
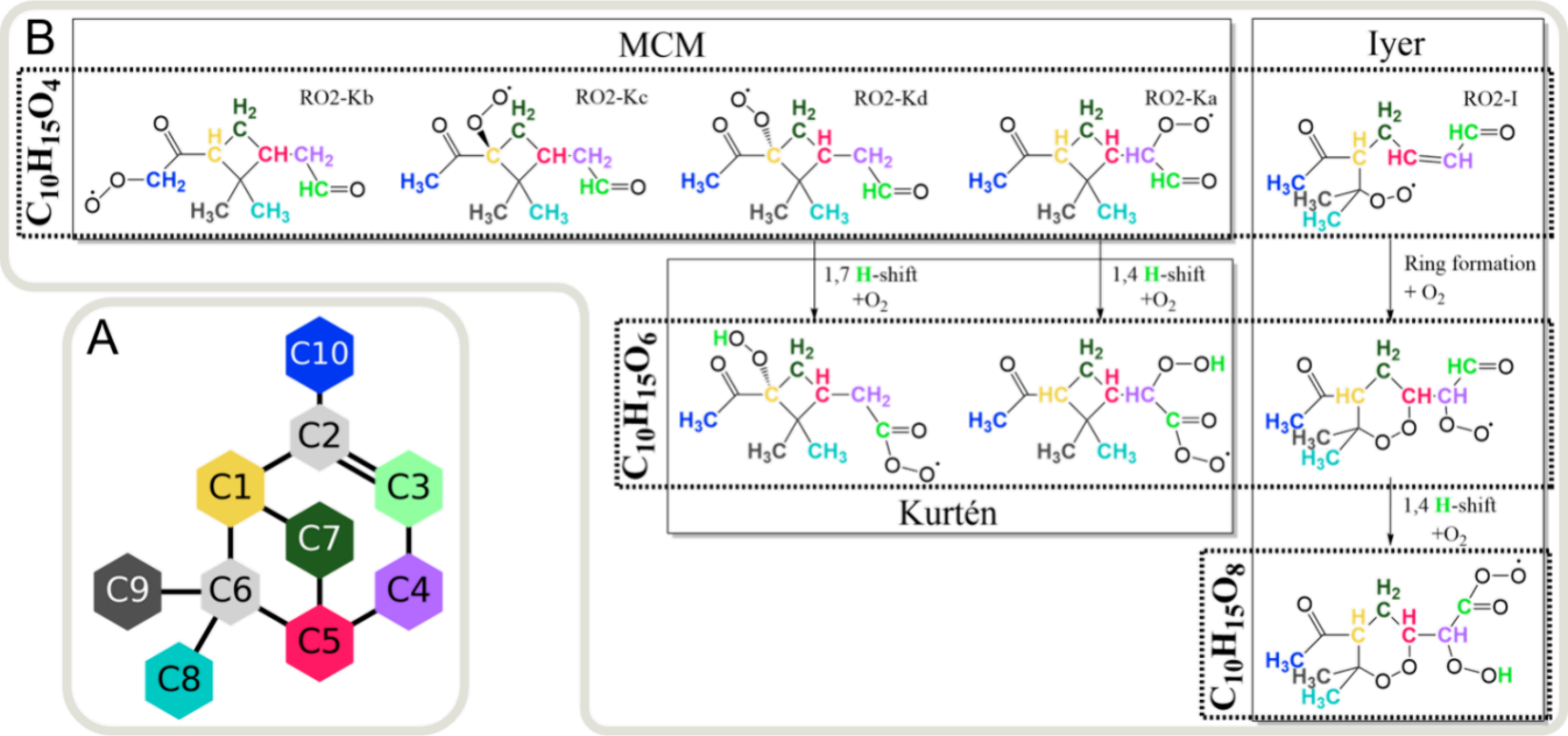
Proposed reaction pathways of the first steps of α-pinene autoxidation. (A) Naming and coloring scheme shown with the α-pinene chemical structure. The number after “C” tells the carbon number (IUPAC) of the carbon that is deuterated. (B) Reaction pathways for α-pinene ozonolysis starting from four-oxygen peroxy radicals O_4_-RO_2_ continuing to six-oxygen peroxy radicals O_6_-RO_2_ (Kurtén) and eight-oxygen peroxy radicals O_8_-RO_2_ (Iyer).^9,10,17^ The carbon and hydrogen atoms are highlighted with colors matching the coloring scheme chosen for the selectively deuterated precursors shown in panel (A). The first-generation peroxy radicals RO_2_–Kb and RO_2_–Kc,d correspond to O_4_-RO_2_ named C109O2 and C107O2 in the Master Chemical Mechanism, respectively.^17^

In this work, we inspect α-pinene ozonolysis and the subsequent autoxidation using a novel set of synthetic precursors to identify which proposed mechanisms need expansion or even rectification. We performed flow reactor experiments for which we synthesized a full set of eight unique selectively deuterated α-pinene analogues in which all hydrogen atoms (^1^H) were replaced with deuterium atoms (^2^H, denoted D hereafter) for each of the eight distinctive carbon atoms (C) with C–^1^H bonds (Figure 1A).^16^

This type of isotopic labeling enables probing which carbon atoms undergo H-shifts in the autoxidation process and H-loss in other reactions. In autoxidation, H-shifts lead to labile H in hydroxyl and hydroperoxy functional groups that can be exchanged with H from water vapor or with other labile H (both ^1^H and D; Section S1).^18^ When the exchange of H leads to ^1^H/D-exchange (H-loss), it can be observed using mass spectrometry, which normally only provides the elemental composition without structural information. Additionally, one can determine the number of total labile H in the oxidation products by using heavy water (D_2_O).^19^ We therefore determined the distribution of total labile H in each of the most prominent more-oxidized products using nonlabeled α-pinene and D_2_O. We utilized a high mass-resolving power (280 000 Th/Th) Chemical Ionization Orbitrap mass spectrometer (CI-Orbitrap) required for the advanced isotopic labeling methods employed in this study, together with two reagent ions to detect the full range of oxidation products. The acquired data provides unprecedented information on the oxidation pathways of the system, allowing us to deduce mechanistic insights of both the initial steps as well as the later autoxidation.

## Experimental Section

We conducted flow reactor experiments with the eight selectively deuterated α-pinene analogues and heavy water (D_2_O) experiments to infer the number of labile H species in the oxidation products. Additionally, we conducted quantum chemical (QC) calculations to explain the findings regarding C_10_H_16_O_3_ from the selectively deuterated precursor experiments. We will give a brief description of the methods used next, while the extended experimental information can be found in Section S2.

### Materials for Flow Reactor Experiments

The selectively deuterated analogues were synthesized for this study, and information on their synthesis can be found elsewhere.^16^ We used α-pinene (purity 98%, Sigma-Aldrich) for all nonlabeled α-pinene and D_2_O experiments and heavy water (deuteration degree min. 99.9% for NMR spectroscopy MagniSolv) in the D_2_O experiments. For generating CI-inlet reagent ions, we used *N*-butylamine C_4_H_9_NH_2_ (purity >99.0%) and nitric acid (purity >60%).

### Flow Reactor Experiments

We studied α-pinene ozonolysis using selectively deuterated precursors in a 0.7 l cylindrical glass flow reactor (L (59.5 ± 1.0) cm, i.d. (40 ± 1) mm) with a constant ozone concentration of around 100 ppb at (23 ± 1) °C. The schematic of the experimental set up is shown in Figure S3. We injected the precursor into the flow reactor using a 0.5 lpm nitrogen flow and a syringe pump (Fusion F100T2 digital dual syringe pump, Chemyx Inc., USA), so that we could monitor reactions happening at different precursor concentrations in the flow reactor. We generated the ozone using an ozone generator (Dasibi 1008-PC) and clean air from a zero-air generator (AADCO, Series 737-14, Ohio, USA), and we injected it into the flow reactor at a 6 lpm flow rate. We used zero air from the zero-air generator as an additional carrier flow aiming at a (15.5 ± 0.2) lpm total flow through the flow reactor, resulting in a roughly 3 s residence time.

We monitored the precursor and other VOC concentrations in the flow reactor using a Vocus PTR-ToF (Vocus PTR, Tofwerk AG),^20^ and we monitored the oxidation products using a chemical ionization Orbitrap (CI-Orbitrap; Q Exactive Plus Orbitrap, Thermo Scientific). We utilized protonated *N*-butylamine C_4_H_9_NH_3_^+^ as the reagent ion in the positive mode to monitor the less oxygenated oxidation products (1–6 oxygen atoms in compound)^6,21^ and nitrate NO_3_^–^ in negative mode to monitor products with seven or more oxygen atoms (Figure S4). We used sensitivity-optimized settings to ensure proper monitoring of the less abundant signals.^22^ We used a photometric ozone analyzer (model 400, Teledyne Instruments, USA) to monitor the ozone concentration. The relative humidity was not monitored constantly, but when monitored with a RH probe (HMP110, Vaisala), it was below the measurement range of the probe 0 ± 1.5%. We used mass flow controllers (G-series, MKS, USA) to control the flow rates and measured the total flow using a flow meter (Mass Flow Meter 4043, TSI, USA).

To get the total number of exchangeable hydrogen atoms in α-pinene (aut)oxidation products, we conducted the experiments using the same setup, but we added a humidified zero air flow to control the relative humidity in the flow reactor. We used a bubbler with 50 mL of water (H_2_O) or heavy water (D_2_O) and a 3 lpm zero air flow through the bubbler. We adjusted the flow reactor carrier flow accordingly to achieve the (15.5 ± 0.2) lpm total flow. We achieved roughly 10% relative humidity with these settings, which was enough to exchange the hydrogen from over 90% of the nitrate dimer signal to a deuterium. These experiments were conducted using only nitrate CI, as *N*-butylaminium was suboptimal due to it having three exchangeable hydrogens and thus proved challenging to use for this experiment.

Due to the lack of calibration standards for the products of interest, there are high uncertainties associated with the values reported in this study. Regardless, we minimize the effect of these uncertainties on our main conclusions by inspecting relative values of signals instead of using the estimated concentrations of the products directly. The nitrate spectra are more consistent over the years than the aminium spectra when considering the repeatability of the values over experiments done in different years (Figure S5). We consider <10% signal fractions to be insignificant and associate a ± 15 percentage point uncertainty to the signal fraction values. Additionally, we only consider a change in measured product yield significant if the observed value is at least double the reference from nonlabeled α-pinene spectrum or smaller than half of the reference.

### Quantum Chemistry

Barrier heights of the different isomerization pathways of α-pinene-derived Criegee intermediates were calculated using the conformational sampling protocol adapted from Mo̷ller et al.^23^ To study the fate of the α-pinene-derived Criegee intermediates, a full configurational sampling was carried out using the Spartan ‘20 program (Wavefunction, Inc.) with the MMFF method. Density functional methods (DFT) with ultrafine grid were used for geometry optimization and frequency calculations of the systems, first at the B3LYP/6–31+G(*) level^24−28^ and subsequently at the ωB97X-D/aug-cc-pVTZ^29−31^ level of theory. Gaussian 16 program was used for these computations.^32^ Unrestricted-DFT with the keyword guess(mix,always) was used for the optimization of the transition states (TS). Final energies were corrected by computing single-point electronic energies at the RHF-RCCSD(T)-F12a/VDZ-F12 level of theory.^33−36^ The CCSD(T) calculations were carried out using the Molpro 2022.2.2 program.^37^

When considering the dioxiranes, the reactive complex, TS, and product compounds are multireference because the ground state is an open-shell singlet. Multireference methods are therefore needed to calculate the activation barrier correctly. The extended multistate complete active space perturbation theory at the second-order (XMC-CASPT2)^38^ calculation was performed using the BAGEL software.^39^ The tzvpp basis set with 8 electrons in 7 molecular orbitals (MOs) and svp basis set with 6 electrons in 4 MOs were used for the model (methyl-dioxirane) and real (α-pinene dioxirane) systems, respectively.

## Results and Discussion

**Figure 2:**
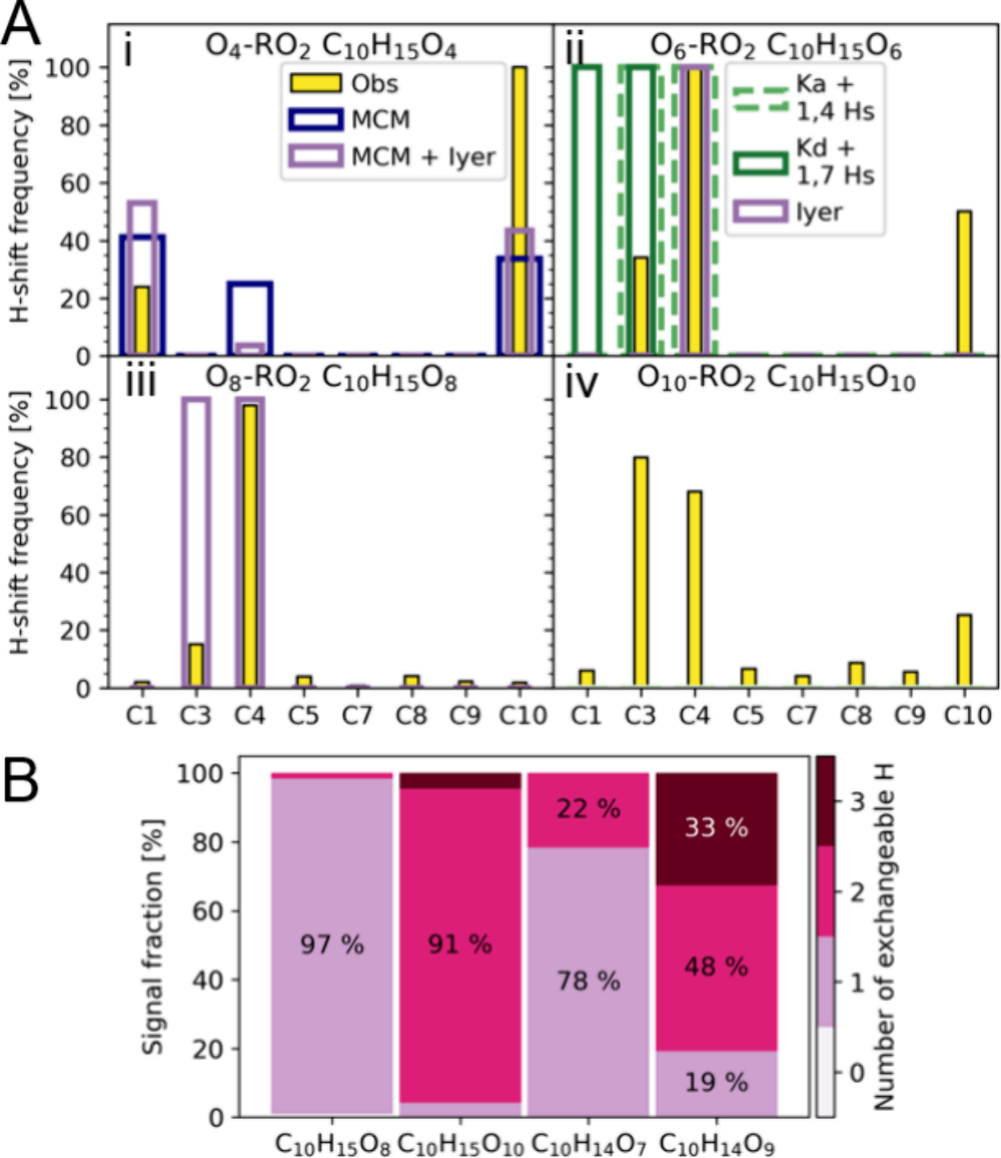
(A) Comparison of observed selective deuteration data and computational estimates. Computational estimates (unfilled bars) and observed (Obs, yellow bars) fractions of signal that has undergone a carbon-specific H-shift. The observed values are for peroxy radicals C_10_H_15_O_4,6,8,10_ at around 2.1 · 10^9^ cm^–3^ reacted precursor. The computational estimates for (i) C_10_H_15_O_4_ are from the Master Chemical Mechanism (navy blue, MCM) and MCM accounting for the fast reaction of RO_2_–I (lilac, MCM + Iyer). The computational estimates for (ii) C_10_H_15_O_6_ and (iii) C_10_H_15_O_8_ are from the 1,4 H-shift and oxygen addition of RO_2_–Ka (green dashed line, Ka + 1,4 Hs), the 1,7 H-shift and oxygen addition of RO_2_-*K*_d_ (dark green, *K*_d_ + 1,7 Hs), and the Iyer mechanism (lilac, Iyer), and they assume that all of the signal is formed through one computational pathway. There are no computational mechanisms proposed for the formation of C_10_H_15_O_10_. (B) Labile H distributions. Signal fractions of products corresponding to C_10_H_15_O_8, 10_ and C_10_H_14_O_7, 9_, where the color shows the observed number of exchangeable hydrogen atoms in the compounds based on H/D-exchange experiments using D_2_O. Bars that are below 5% are not annotated.

The selectively deuterated precursors have one (precursors C1, C3, C5), two (C4, C7), or three (C8, C9, C10) ^1^H exchanged for D (Figure 1A). When an H-shift has taken place from a specific carbon position, we refer to it as “Cx H-shift”. For example, we see from Figure 2A that C4 has always undergone an H-shift (C4 H-shift) when forming C_10_H_15_O_8_ (O_8_-RO_2_). During oxidation, the molecule can lose D through the H/D-exchange as a result of H-shifts: anywhere from zero to the number of D originally in the deuterated precursor can be lost, and twice as many D can be lost from the accretion products. Note that H-loss can happen in other ways during, e.g., termination (Section S3), and those are considered separately in our discussion. To simplify the analysis and limit interference from OH oxidation, we focus our main inspection on compounds that are known to form only through α-pinene ozonolysis, namely, RO_2_ with an even number of oxygen atoms C_10_H_15_O_even_ and closed-shell products with 14 hydrogen atoms and an odd number of oxygen atoms C_10_H_14_O_odd_.^11,40^ For the C8 precursor, the inspection of compounds is limited to only RO_2_ due to contamination affecting other signals (Section S4).

For each inspected composition, we can infer how frequently a carbon-specific H-shift takes place from the distribution of the signals with different numbers of D in the mass spectrum (see Section S1 for more details). We show how frequent H-shifts from the deuterated carbon positions are for the different radical species and compare them to previously proposed mechanisms (Figure 2A). The experimental results are depicted with filled bars, while the expected behavior based on previously proposed mechanisms is depicted with unfilled bars. The H-shift frequency describes the fraction of the total signal assigned to one molecular composition that has lost a D, i.e., undergone an H-shift from the deuterated position. For example, in Figure 2A, panel ii, the H-shift frequency for C10 H-shift is 50% based on our experimental data, which suggests that half of the observed O_6_-RO_2_ radicals have undergone an H-shift from carbon position 10, while the other half have not. This suggests the existence of at least two isomeric forms of this radical. Furthermore, a single species can undergo multiple H-shifts during its formation, which will lead to the sum of H-shift frequencies exceeding 100%. For example, in Figure 2A, panel ii, forming the O_6_-RO_2_ only through the computational mechanism called Ka + 1,4 Hs necessitates both a C3 and a C4 H-shift during the formation of the product. As a result, the predicted H-shift frequency is 100% for both positions, amounting to a 200% total H-shift frequency.

### Validating Proposed Autoxidation Mechanisms

We start with α-pinene ozonolysis autoxidation products with more than 4 oxygen atoms as these products are observed in the atmosphere, and the mechanisms associated with them are thought to have higher uncertainties.^4^ Only recently, a sufficiently rapid mechanism was discovered where the formation of up to eight-oxygen-atom-containing peroxy radicals C_10_H_15_O_8_ (O_8_-RO_2_) was possible through chemically activated vinoxy radical ring-breaking (Iyer mechanism; Figure 1B).^9^ This mechanism can explain a significant portion of the observed RO_2_ (Figure 2A) and the closed-shell ozonolysis product C_10_H_14_O_odd_ signal (Figure S12B). The Iyer mechanism predicts the formation of an O_6_-RO_2_ that has only undergone a C4 H-shift. Our experimental data show that all of the O_6_-RO_2_ have undergone a C4 H-shift; however, some have also undergone C3 and C10 H-shifts (Figure 2Aii). The O_6_-RO_2_ results can be explained if most of this radical species has undergone two separate H-shifts, one from the C4 and another from either the C3 or C10 positions. These would account for roughly 80% of the O_6_-RO_2_ signal, meaning that around a fifth of the observed O_6_-RO_2_ signal has undergone only a C4 H-shift and can thus be explained by the Iyer mechanism. Additionally, the observed signal of the closed-shell product C_10_H_14_O_5_ that would form from O_6_-RO_2_ can mostly be explained by the Iyer O_6_-RO_2_ terminating bimolecularly into a ketone (Figure S7), thus losing two H atoms from C4 during its formation (Figure S12).

Roughly a third of the O_6_-RO_2_ signal can be explained by another previously proposed mechanism. A quantum chemical computational study of O_6_-RO_2_ formation from the first-generation RO_2_ (O_4_-RO_2_) could only find two H-shifts taking place with rates faster than 0.1 s^–1^, a threshold often used to indicate competitiveness against bimolecular reactions (Figure 1B, Kurtén mechanisms).^10^ The Kurtén mechanism where the first-generation peroxy radical RO_2_–Ka has undergone a C3 H-shift can explain a third of the O_6_-RO_2_ signal, as it has undergone the required C4 and C3 H-shifts when formed (Ka + 1,4 Hs in Figure 2Aii). Nevertheless, a significant fraction of the O_6_-RO_2_ signal remains unexplained, as no fast-enough computational mechanism for the formation of O_6_-RO_2_ where a C10 H-shift takes place has been proposed.

The O_8_-RO_2_ signal can be explained by the Iyer mechanism, considering the significant decrease in the signal of the O_8_-RO_2_ for the C3 precursor. Using the selectively deuterated precursors may lead to a decreased yield of the products due to the kinetic isotope effect (KIE).^41^ We compared the yields of HOMs measured when using deuterated precursors to those when using nonlabeled α-pinene and observed a significant decrease in the yield of O_8_-RO_2_ when using the C3 precursor (Figure S19A). To form Iyer O_8_-RO_2_, the molecule has to undergo a C4 and a C3 H-shift. Accordingly, KIE significantly affects the C3 precursor at the step from the O_6_-RO_2_ to the O_8_-RO_2_ where the H-shift from C3 would take place. This is seen as the O_6_-RO_2_ signal not being affected, but the O_8_-RO_2_ signal being significantly decreased along with the closed-shell product C_10_H_14_O_7_ signal (Figure S19A). Roughly half of the C_10_H_14_O_7_ signal can be explained by termination from the O_8_-RO_2_–I, as it has lost two D when using the C4 precursor and one D when using the C3 precursor (Figure S12B), corresponding to a molecule that has undergone C4 and C3 H-shifts and then a C4 H-shift during unimolecular termination (Figure S7). The Iyer O_6_ to O_8_-RO_2_ mechanism is further supported by the number of exchangeable hydrogens in the observed O_8_-RO_2_ and C_10_H_14_O_7_ (Figure 2B). Our D_2_O experiments show that the O_8_-RO_2_ signal has one hydroxyl or hydroperoxy group, matching the Iyer O_8_-RO_2_ structure (Figure 1B). Additionally, the majority of the C_10_H_14_O_7_ signal has one exchangeable hydrogen, which corresponds to the closed-shell product formed from Iyer O_8_-RO_2_ (Figure S7).

There is no proposed computational mechanism for the formation of peroxy radicals with ten oxygen atoms (O_10_-RO_2_). Regardless, our data show that C3, C4, or C10 H-shifts take place when forming these compounds (Figure 2Aiv). O_10_-RO_2_ should also have two hydroxyl or hydroperoxy groups based on our D_2_O experiments (Figure 2B). Part of the signal could be explained by continuing the Iyer pathway, requiring the formation of one hydroxyl or hydroperoxy group. This is supported by the significant signal decrease observed for O_10_-RO_2_ in the C3 precursor spectrum (Figure S19A), implying that the O_8_-RO_2_ products whose signals are decreased go on to form the observed O_10_-RO_2_. However, around a fifth of the O_10_-RO_2_ signal cannot be formed via the Iyer mechanism as it has formed without going through an H-shift from C4 (Figure 2Aiv).

Moving to the less oxygenated products, we find that the computational predictions based on the MCM for the first-generation four-oxygen peroxy radicals (O_4_-RO_2_) underestimate the significance of RO_2_–Kb and overestimate RO_2_–Kc,d (Figure 2Ai).^17^ It should be noted that MCM does not predict any formation of RO_2_–Ka as it expects the stabilized Criegee intermediate to undergo CO fragmentation instead. We get the value used in Figure 2A by assuming that the stabilized Criegee intermediate forms RO_2_–Ka instead. The discrepancies between the observations and computational estimates remain even when the fast ring-formation and oxygen-addition reactions that deplete the observed signal of RO_2_–I of the Iyer pathway are accounted for, as the C1, C4, and C10 H-shift frequencies do not match the values from the proposed mechanisms (Figure 2Ai).^9^ We can observe products that have undergone C1 and C10 H-shifts that are needed to form RO_2_–Kc,d and RO_2_–Kb, respectively. However, we see no signal for the C4 H-shift products, which is required to form RO_2_–I/Ka. Accounting for the signal increase of O_4_-RO_2_ for the C10 precursor (Figure S25), we can explain our observation as one less-prominent product formed undergoing a C1 H-shift and another more-prominent product formed undergoing a C10 H-shift. The discrepancy between observation and estimates can lead to misrepresentation of the autoxidation process, as MCM is often used as a basis of models and mechanistic studies.^10,13,14^

### Explaining C_10_H_16_O_3_

Contrary to a commonly used assumption,^42,43^ our data suggests that the C_10_H_16_O_3_ signal is not predominantly pinonic acid formed via the hot acid pathway, as the signal is mostly composed of compounds that have had no H-loss take place in their formation (Figure S12E). In the hot acid pathway, first, a dioxirane is formed from a Criegee intermediate (APINOOB in MCM), which is then assumed to quickly convert to pinonic acid after undergoing a C3 H-shift.^17^ We believe the observed C_10_H_16_O_3_ signal is from ozonolysis and not significantly contaminated by OH oxidation, because the yield of C_10_H_16_O_3_ matches well with a previously reported value of ozonolysis-only oxidation (Figure S26C).^42^ Additionally, the hot acid pathway is assumed to be very dominant and is thus expected to explain a significant fraction of the signal even with OH oxidation interference, which is contradicted by our data. At most, a third of the signal has undergone a C4 or a C10 H-shift (Figure S12E) and can thus be explained by OH roaming or a bimolecular reaction between RO_2_–Kb or RO_2_–Ka and other RO_2_ where the forming C_10_H_16_O_3_ is an alcohol (Section S3). To explain the no-H-loss signal, we performed quantum chemical calculations and found that the signal can potentially be explained by two products (Section S5). The first is the secondary ozonide (SOZ) that forms from two Criegee intermediates (APINOOB and APINOOA in MCM) before any H-shifts have taken place. The second is the dioxirane forming from another Criegee intermediate (APINOOB in MCM), and it is currently assumed to be unstable in MCM.^17^ Nonetheless, our QC calculations show the dioxirane to be stable enough to be observed, and its formation is supported by our observations.

### H-Shifts from a Few Specific Carbons in α-Pinene Autoxidation

**Figure 3:**
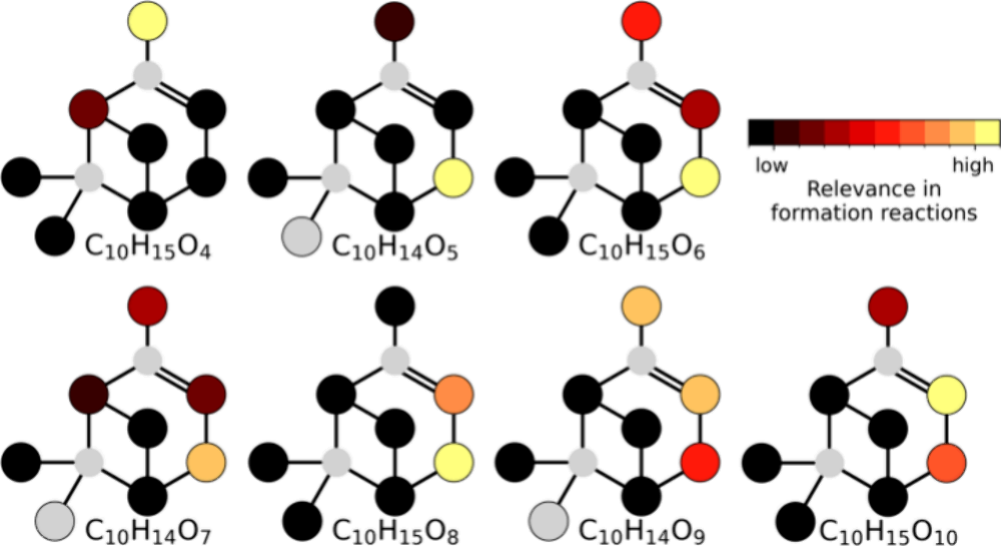
Significance of carbon position in forming α-pinene ozonolysis (aut)oxidation products. The color shows the fraction of signal of the product that has undergone at least one carbon-specific H-shift or H-loss divided by relative yield for each precursor shown on the structure of α-pinene averaged over experiments with 3.3 · 10^8^ to 2.1 · 10^9^ cm^–3^ reacted precursor. The fractions of signal for compounds with 6 or less oxygen atoms are not corrected with the relative yield. The relative yield is the observed yield of the product from the oxidation of a deuterated precursor relative to the yield of that product from the oxidation of nonlabeled α-pinene. The compounds included are monomers C_10_H_14_O_odd_ and radicals C_10_H_15_O_even_ that are known to form through only ozonolysis. The light gray color signifies missing data.

Looking at the broader trends in α-pinene autoxidation, we find that most H-shifts take place from surprisingly few carbons, namely, positions C3, C4, and C10, for both monomeric species (Figure 3) and accretion products (Figure S11B) despite the complex structure of the α-pinene molecule. The accretion products and lower-oxygenated monomeric species show indications for C1 and C5 H-shifts (Section S6); however, these and the other positions are not nearly as relevant as C3, C4, and C10 in forming the observed oxidation products at the 3 s time scale studied here, though this may change for later-generation oxidation. Nevertheless, this helps guide future endeavors to elucidate remaining unknowns in this system, potentially allowing for studies using simpler surrogate molecules where some nonreacting carbon atoms are left out.

Though used already decades ago, advanced isotopic labeling approaches have found new use here and in other recent studies, representing the most promising methodological way forward in experimentally assessing complex oxidation chemistries.^42,44−46^ However, given the highly structure-specific nature of (aut)oxidation pathways seen from our and earlier work, results from one system cannot be easily expanded to others. Unfortunately, these approaches require immense efforts, making it unfeasible to do such work for all relevant VOCs, let alone all monoterpenes or even α-pinene ozonolysis over longer time scales. We thus hope our data set^47^ will serve as a critical validation benchmark for future computational approaches revealing the most important pathways.

### Implications for Modeling Autoxidation

**Figure 4:**
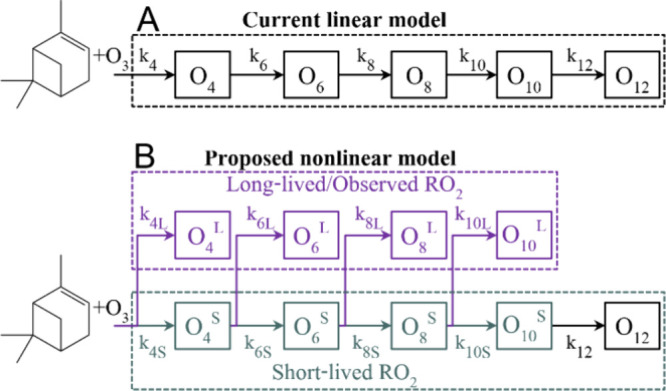
Current and proposed improved autoxidation modeling schemes. (A) Current linear modeling scheme for autoxidation where peroxy radicals with *n* oxygen atoms (O_n_) produce the next peroxy radicals with added oxygen (O_*n*+2_) at reaction rates *k*_*n*_ that are the same for all O_*n*_. (B) Nonlinear modeling scheme where the peroxy radicals are separated in short-lived peroxy radicals (O_*n*_^S^, teal) and long-lived and thus observable peroxy radicals (O_*n*_^L^, purple). The short-lived RO_2_ continues to autoxidize, forming the basis for the formation of the more oxygenated peroxy radicals. Contrastingly, autoxidation does not continue for the long-lived RO_2_ molecules, leading to their longer lifetimes. The reaction rates forming short-lived O_*n*_-RO_2_ are *k*_*n*S_, and the long-lived ones are *k*_*n*L_. In both schemes, the subsequent reactions are not shown for clarity.

An interesting observation from the previous discussion is that many of the less-oxygenated RO_2_ have undergone carbon-specific H-shifts that the higher-oxygenated RO_2_ have not. For example, O_4_-RO_2_ have undergone C1 and C10 H-shifts, while O_8_-RO_2_ have undergone C4 and C3 H-shifts and no C1 or C10 H-shifts. This means that the observed O_8_-RO_2_ cannot be formed from the observed O_4_-RO_2_. We also observe similar behavior in the RO_2_ + RO_2_ accretion product data (Section S6). These findings suggest that autoxidation in this system does not progress “linearly” and thus should not be modeled as a linear process (Figure 4A) where lumped O_4_-RO_2_ form O_6_-RO_2_, which then form O_8_-RO_2_ and so on. In contrast, many current models assume this linear progression, where the higher-oxygenated products are necessarily formed from the lower-oxygenated products.^13−15^ Instead, our results suggest that autoxidation should be modeled as a nonlinear process (Figure 4B). This can be done by including more than one RO_2_ species with a specific number of oxygen atoms in the models. The species are divided into long-lived RO_2_ that accumulate to model the observed high concentrations, and short-lived RO_2_ that quickly form the more oxygenated RO_2_. The long-lived RO_2_ can accumulate and are thus more abundant and subsequently dominate the observed RO_2_, although the total formation rate may be lower than that for the short-lived counterparts.

Such nonlinearity is well established in many mechanisms where only specific isomers can undergo said reactions, a prime example being the discussed Iyer mechanism.^9^ Furthermore, “nonlinear” models have already been implemented for some monoterpenes, but not for α-pinene.^48^ Additionally, we believe that accounting for the nonlinearity is also important for molecules other than α-pinene. In fact, we find that the observed RO_2_ kinetics during cyclohexene ozonolysis^49^ can be best explained with a model that allows independent, i.e., nonlinear, pathways to the O_6_-, O_8_-, and O_10_-RO_2_ radicals (Section S7).

## Conclusions

We studied α-pinene ozonolysis and autoxidation using selectively deuterated α-pinene analogues and heavy water in a flow reactor to gain mechanistic insights into the processes. We found that the Iyer mechanism could fully explain the observed O_8_-RO_2_ signal and most of the closed-shell product signals formed from them. Additionally, the mechanism could explain roughly one-fifth of the observed O_6_-RO_2_ signal and much of the closed-shell product formed from O_6_-RO_2_ through bimolecular termination. Nevertheless, we found indications in our data that RO_2_ are formed also through other mechanisms. Additionally, we found that the observed C_10_H_16_O_3_ signal could not be explained by the hot acid route; instead, the signal could be explained by the α-pinene-derived secondary ozonide and dioxirane based on our experimental findings and quantum chemical calculations. Surprisingly, few carbon atom positions, namely, positions 3, 4, and 10, participated in H-shifts during the formation reactions of most of the observed products.

Our data also highlighted the fact that α-pinene ozonolysis and autoxidation necessarily happen through multiple mechanisms, and that should be accounted for in oxidation models used to predict atmospheric aerosol behaviors. A correct kinetic and mechanistic description of the RO_2_ is important for assessing how autoxidation and HOM formation are impacted by atmospheric composition such as NO concentrations. If the H-shifts forming the most oxygenated RO_2_ are very fast, even moderate NO concentrations will not greatly hamper their occurrence, allowing for efficient OA formation under polluted conditions. Moreover, to accurately parametrize observations for implementation into atmospheric models, our findings suggest that autoxidation is likely best captured by nonlinear autoxidation schemes. This, in turn, is critical for correctly predicting SOA formation in the future warming climate, where NO and NO_2_ levels decrease following stricter air quality standards.

## Supporting Information Available

Data interpretation, extended experimental section, α-pinene ozonolysis and autoxidation and closed-shell product formation, C8 contamination determination, QC findings, accretion product findings, kinetic modeling comparison, applied data corrections, yield calculations, extended heavy water experiment results, and associated figures; yield comparisons; and most prominent observed oxidation products (PDF)
