# Supplementary material for: Selective Deuteration Reveals the Importance of Multiple Branching Pathways in α-Pinene Autoxidation
Source: J Am Chem Soc. 2025 Apr 15;147(17):14131–8. doi: 10.1021/jacs.4c14462 (PMC12046550; doi:10.1021/jacs.4c14462)
Supplement: Supplementary file 1 — ja4c14462_si_001.pdf [file ja4c14462_si_001.pdf]

Supplementary Materials for

Selective deuteration reveals the importance of multiple branching pathways in α-pinene autoxidation

Melissa Meder, Frans Graeffe, Yuanyuan Luo, Jingyi Luo, Siddharth Iyer, Rashid Valiev, Runlong Cai, Matti Rissanen, Theo Kurtén, Jonathan G. Varelas, Franz M. Geiger, Regan J. Thomson, and Mikael Ehn
Corresponding authors: melissa.meder@helsinki.fi, mikael.ehn@helsinki.fi

The PDF file includes:

S1. Interpreting the data....3
Fig. S1. Example mass spectra....4
Fig. S2. Structures and names of all selectively deuterated precursors used in this study.....5
S2. Extended experimental section....6
Fig. S3. The experimental set up.....7
Fig. S4. Precursor comparison.....9
Fig. S5. Comparison of product yields over different measurement years.....17
S3. Pathways for α-pinene ozonolysis and autoxidation, and closed-shell product formation....18
Fig. S6. α-pinene ozonolysis and autoxidation reactions....19
Fig. S7. Unimolecular and bimolecular termination reactions....20
S4. C8 precursor contamination determination....21
Fig. S8. C8 precursor contamination determination.....23
S5. Quantum chemical calculations on $C_{10}H_{16}O_3$....24
Fig. S9. Criegee intermediates from α-pinene ozonolysis.....24
Fig. S10. Isomerization reactions of dioxirane.....25
S6. Carbons partaking in H-shifts in accretion product formation....26
Fig. S11. Heatmap of relevance of H-shifts....28
Fig. S12. Signal distributions....29
S7. Kinetic modeling: comparison of linear and non-linear models....30
Fig. S13. Comparison of linear and non-linear models for cyclohexene autoxidation.....31
S8. Data corrections....32
Fig. S14. Orbitrap data corrections....33
Fig. S15. Comparison to previously measured data....34
S9. Yield calculations....35
S10. Exchangeable hydrogens in HOMs....36
Fig. S16. Labile H distributions....38
Fig. S17. Heavy water data....40
S11. Oxidation product yield comparisons....41
Figs. S18-25. Yields from deuterated precursors against α-pinene....41-47
S12. Estimated yields for selectively deuterated precursor data....50

Fig. S26. Total estimated yields 50
S13. Extended data 51
Table S2. Top 20 yield products..51

S1. Interpreting the data

During oxidation D can be lost from the molecule, the number ranging from zero to all D lost. As a result, for every mass in the α-pinene ozonolysis spectrum, there can be up to four masses that we can observe in the spectrum of each selectively deuterated precursor, as there are at most 3 deuterium atoms in some of the precursors (Figs. S4 and S5). These masses correspond to products that are structurally different as they have undergone a different number of H-shifts or H-loss from different carbons but would have the same chemical formula and thus mass when deuteration is not used. In the case of accretion products ROOR, there can be double the amount of D in the products and thus up to 7 masses that correspond to one mass in non-labelled α-pinene spectrum.

We can infer the number of H-shifts (H referring to both ^{1}H and D) that have taken place from the labelled carbon position from the number of D-atoms lost from an observed product. As an example, the O_4-RO_2 signal of the precursor whose C-atom at position 10 (henceforth "C10") is deuterated (Fig. S4, blue C10) consists fully of $C_{10}H_{13}D_2O_4$ meaning that one D is always lost from C10 during the oxidation. Since the chemical composition has not changed and there are in total 15 H-atoms, we know that a H-shift has taken place and shifted a D to a hydroxide or hydroperoxide group in order to be lost from the compound. This is because when a D-atom is in a hydroxide or hydroperoxide group, it is labile and readily exchanged with ^{1}H when the molecule encounters water or other exchangeable hydrogens. In other words, we find that all observed O_4-RO_2 have undergone a H-shift from C10. H-loss can happen in other ways during e.g. autoxidation termination (Section S2).

In our pilot study, we introduced the methodology used here for a small subset of the deuterated precursors (3D1, 7D2, 10D3 corresponding to C3, C7, and C10) using a continuous-flow stirred-tank reactor.[1] Unfortunately, it was not possible to deduce anything concerning the radical signals due to overlapping and much larger closed-shell signals that result from the gradual RO_2 conversion into oxidation products in the long time-scale experiments. This problem was eliminated by using a short reaction time of 3 s in a flow reactor set-up leaving the radical signals more prominent in both the aminium and nitrate spectra.

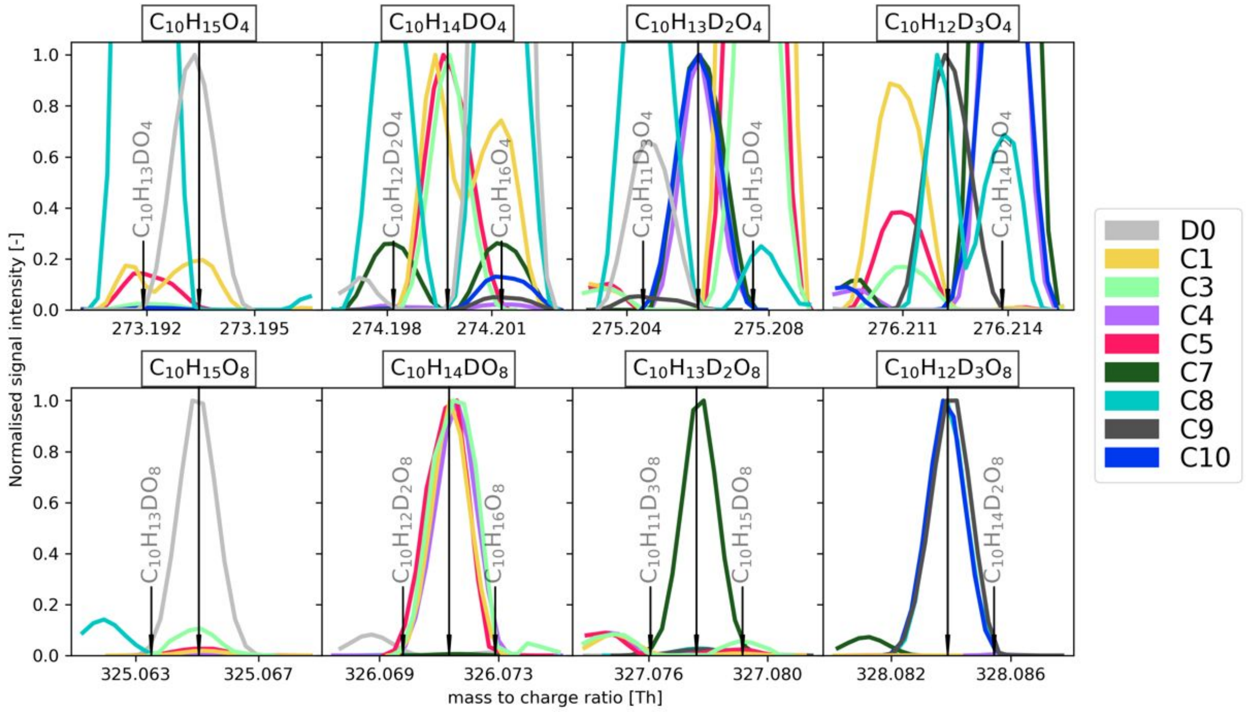

Fig. S1. Example mass spectra

Example mass spectra of $C_{10}H_{15}O_4$ for aminium data (upper row) and $C_{10}H_{15}O_8$ for nitrate data (lower row) for each precursor. The signals are normalized with each precursor's largest radical signal within the inspected range and the signals are measured at reacted precursor $\sim 1.5 \cdot 10^9$ cm^{-3}. If a precursor has lost D-atoms, it reveals that a H-shift of H-loss has taken place from the labeled position. The 'D0' data is from the non-labelled α-pinene experiments. All inspected products are NO_3^- or $C_4H_9NH_3^+$ adducts in nitrate and N-butylaminium data, respectively, and the reagent ion is omitted from the compositions shown for clarity. Note that separating the closely lying peaks requires the high mass-resolving power of the applied Orbitrap instrument.

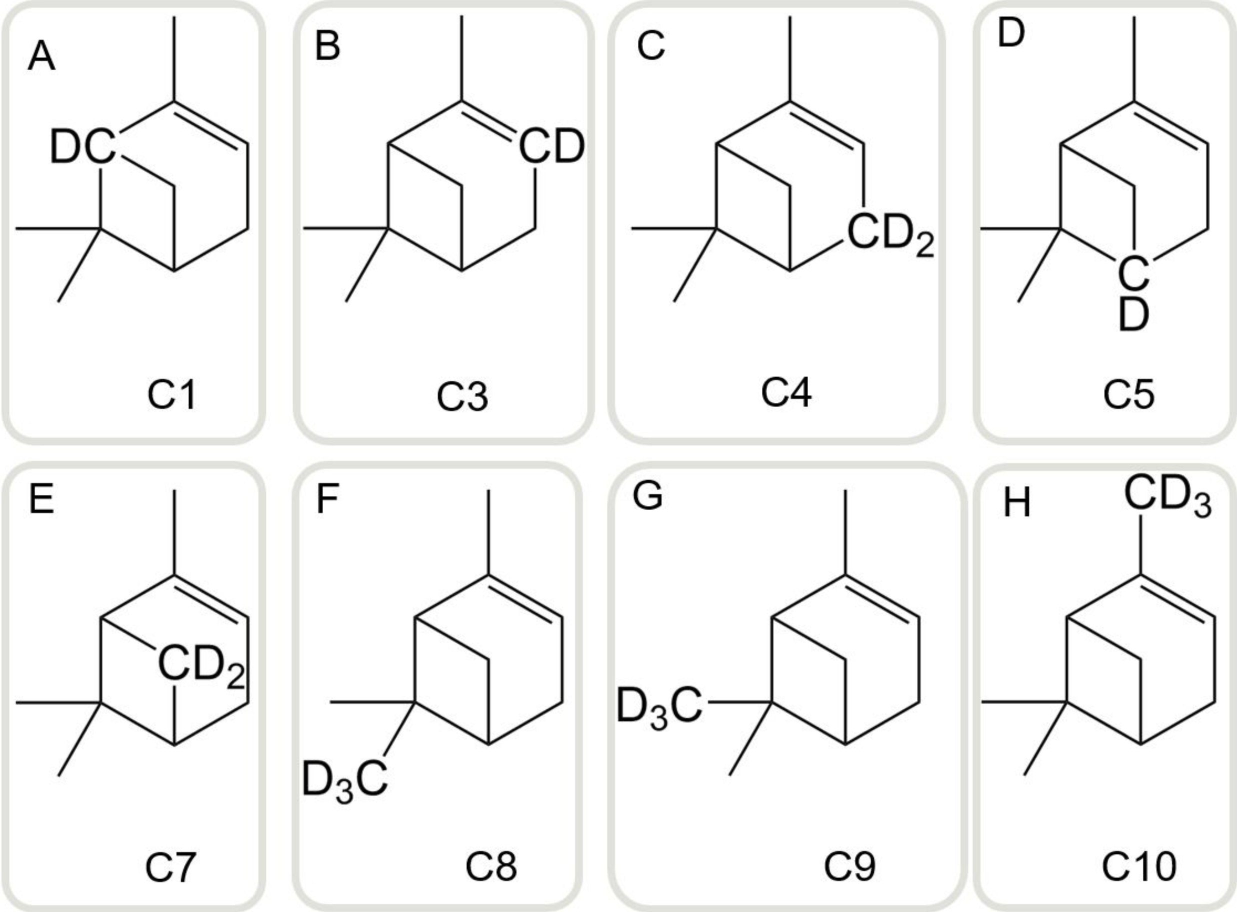

Fig. S2. Structures and names of all selectively deuterated precursors used in this study.
(**A**) α-pinene-1-*d* (C1), (**B**) α-pinene-3-*d* (C3), (**C**) α-pinene-4,4-d_2 (C4), (**D**) α-pinene-5-*d* (C5), (**E**) α-pinene-7,7-d_2 (C7), (**F**) α-pinene-8,8,8-d_3 (C8), (**G**) α-pinene-9,9,9-d_3 (C9), (**H**) α-pinene-10,10,10-d_3 (C10).

S2. Extended experimental section

The experimental set up is shown in Figure S1, and the comparison between nitrate and N-butylammonium CI-Orbitrap data are shown in figure S2. The data on the repeatability of the measurements over the years are shown in figure S3.

The experiment procedure.

We conducted all experiments in the following way. First, we injected only ozone into the flow reactor to measure the background. Second, and only after measuring the background, we inserted the syringe into the set-up and started injecting the precursors. We ramped the precursor concentration in steps by increasing the injection rate, aiming at 20-25, 45-55, 80-95, 110-130, 200-220, and 320 ppb precursor concentrations. We measured each stable precursor concentration step until we had acquired three data points with orbitrap which took roughly 15 minutes per step. The under 100 ppb steps were done to get mostly products that were formed via unimolecular reactions or at most only terminated bimolecularly into monomeric species (i.e., $RO_2 + HO_2 \rightarrow ROOH$). At over 100 ppb precursor concentrations, bimolecularly formed ROOR products started to appear in the spectra. Third, after acquiring the data from the desired concentration steps, we removed the syringe containing the precursor from the set-up and left the instrument to measure. Last, in preparation for the next experiment, we evaporated possible contaminants by heating the flow reactor to 200 ^{o}C, keeping the CI-inlet connected but disconnecting lines for O3 analyzer, Vocus, and RH probe if used. We left the set-up to cool for at least an hour and usually overnight after heating for at least 30 minutes. Before starting the next experiments, the spectra were visually checked to have gone back to background levels.

Data processing

We preprocessed the CI-Orbitrap data with Orbitool (v2.3.0)[2] and Vocus PTR-ToF data with tofTools [3] and tofware (Tofware version 3.2.2, www.tofwerk.com/tofware). We inspected products that are NO_3^- or $C_4H_9NH_3^+$ adducts in nitrate and N-butylaminium data, respectively, and we show the compositions of the products omitting the reagent ion for clarity.

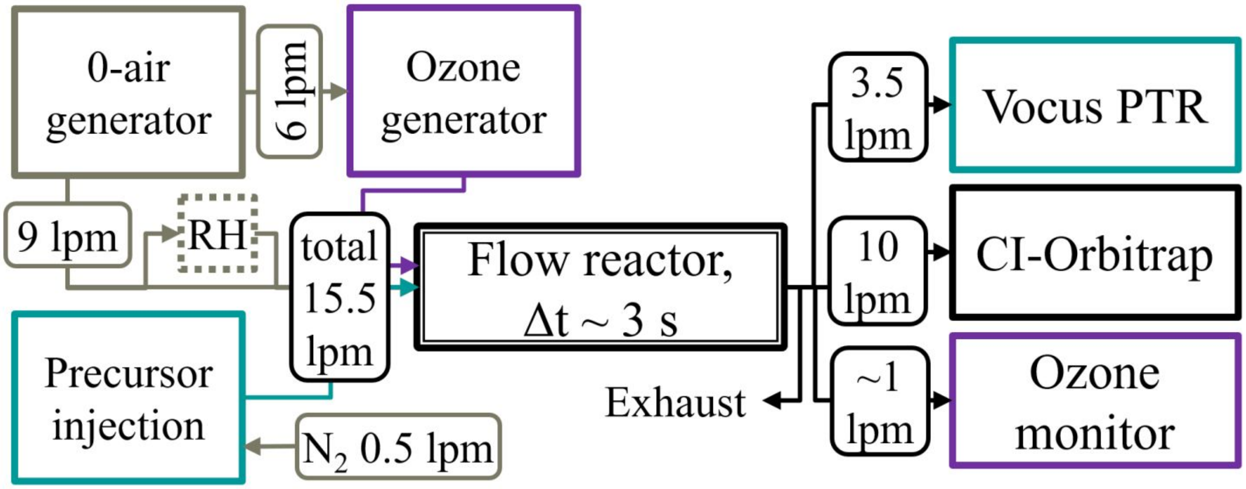

Fig. S3. The experimental set up.

The precursor is injected into a flow reactor where it reacts with ozone and zero air mixture. The residence time in the flow reactor is 3 seconds. The gas mixture is sampled with a CI-Orbitrap and Vocus PTR-ToF and the ozone concentration is monitored with an ozone monitor. Humidification (RH) is added with a bubbler when necessary.

A

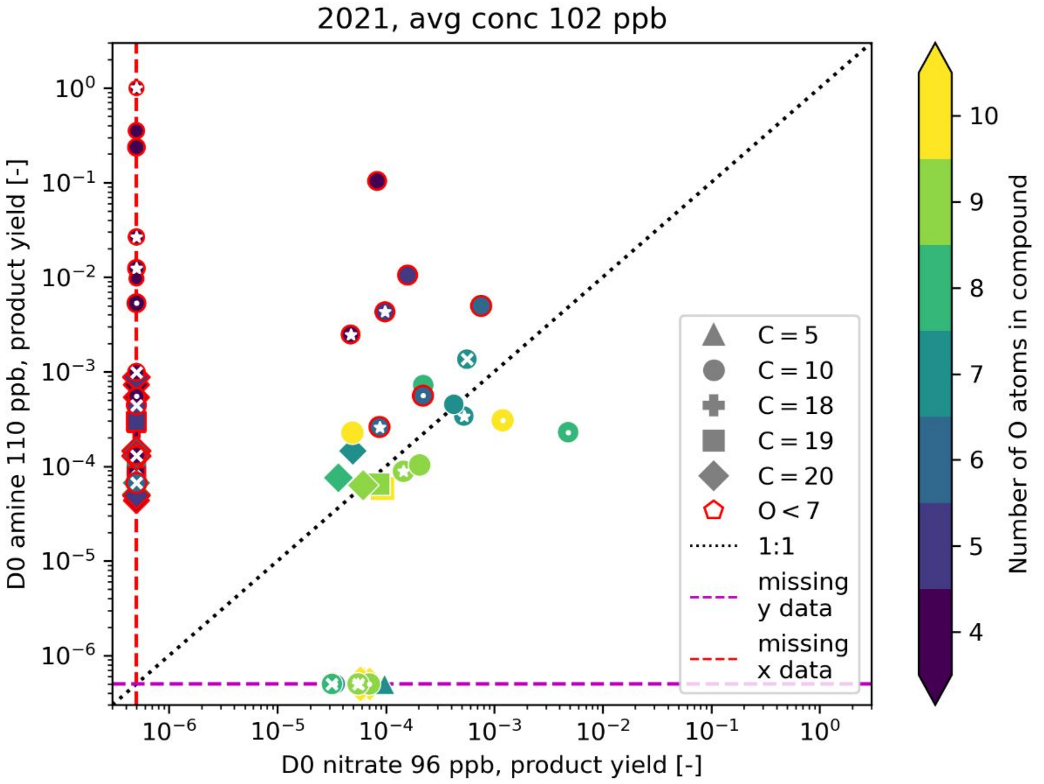

2021, avg conc 102 ppb
D0 amine 110 ppb, product yield [-]
D0 nitrate 96 ppb, product yield [-]
C = 5
C = 10
C = 18
C = 19
C = 20
O < 7
1:1
missing y data
missing x data
Number of O atoms in compound

B

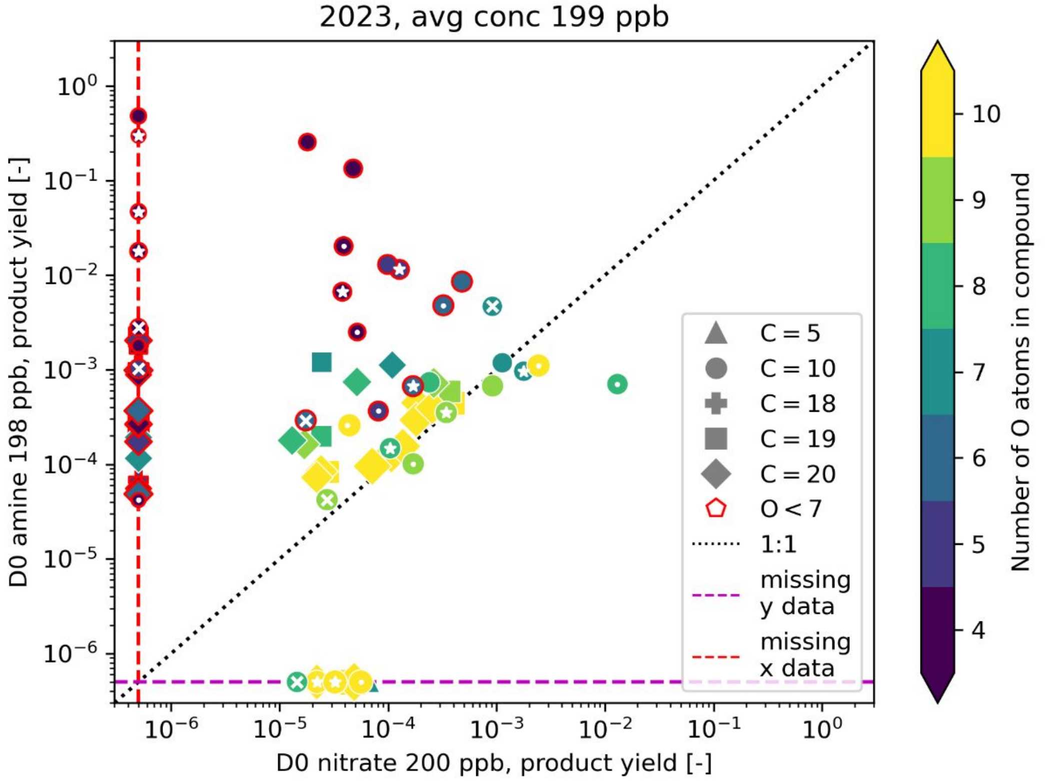

2023, avg conc 199 ppb
D0 amine 198 ppb, product yield [-]
D0 nitrate 200 ppb, product yield [-]
C = 5
C = 10
C = 18
C = 19
C = 20
O < 7
1:1
missing y data
missing x data
Number of O atoms in compound

C

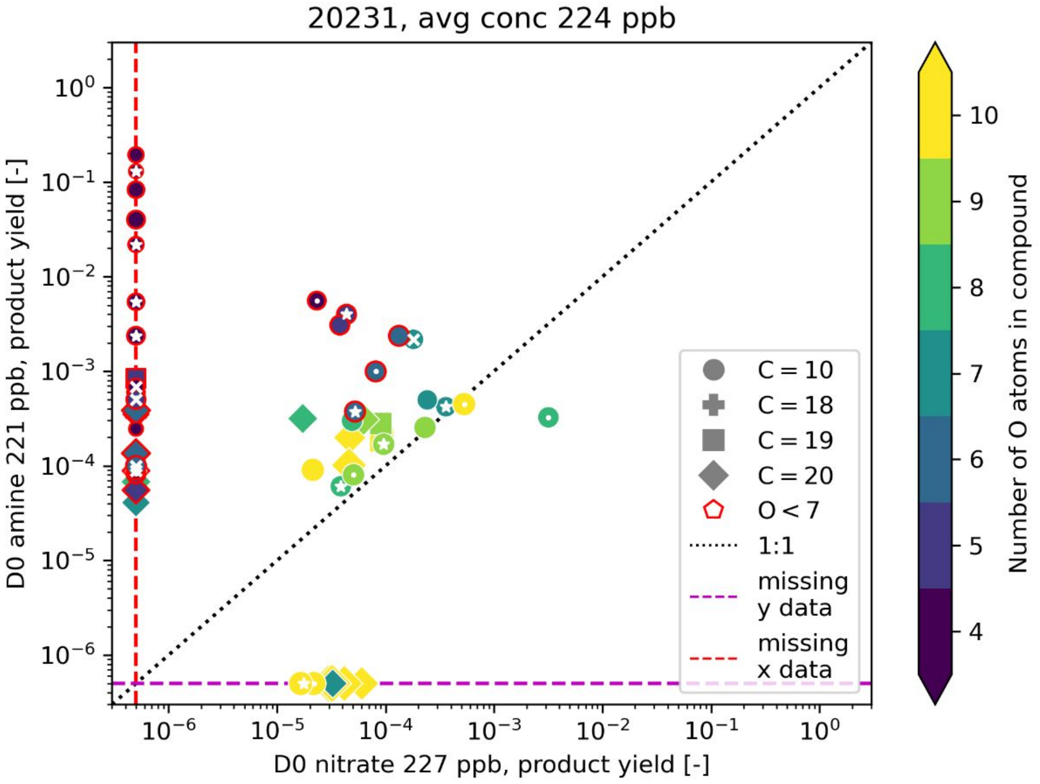

Fig. S4. Precursor comparison.

Yields of oxidation products for N-butylaminium data against those for nitrate data where the color shows the number of oxygen atoms in the products. The data is from (**A**) 2021, (**B**) 2023 January and February, and (**C**) 2023 December using non-labelled α-pinene (D0). The products with less than 7 oxygen atoms are marked with red circles, and products $C_{10}H_{14}O_n$ are marked with a white star, $C_{10}H_{15}O_n$ with a white dot, and $C_{10}H_{17}O_n$ with a white cross.

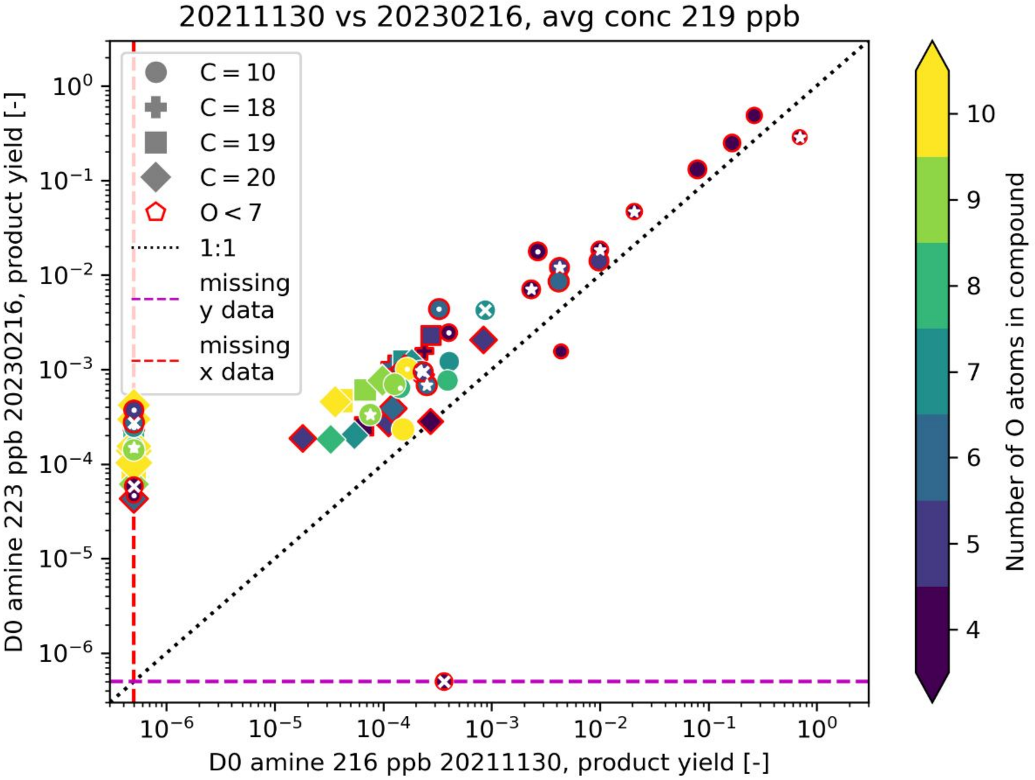
20211130 vs 20230216, avg conc 219 ppb
C = 10
C = 18
C = 19
C = 20
O < 7
1:1
missing y data
missing x data
D0 amine 223 ppb 20230216, product yield [-]
D0 amine 216 ppb 20211130, product yield [-]
Number of O atoms in compound

A

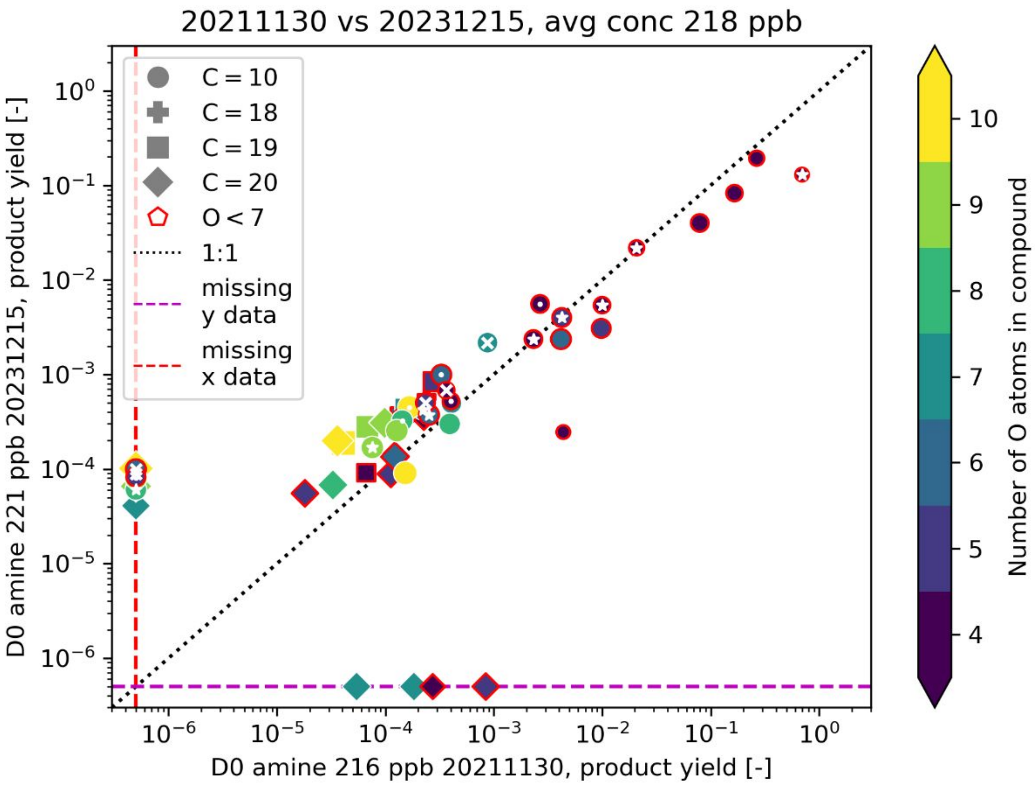
20211130 vs 20231215, avg conc 218 ppb
C = 10
C = 18
C = 19
C = 20
O < 7
1:1
missing y data
missing x data
D0 amine 221 ppb 20231215, product yield [-]
D0 amine 216 ppb 20211130, product yield [-]
Number of O atoms in compound

B

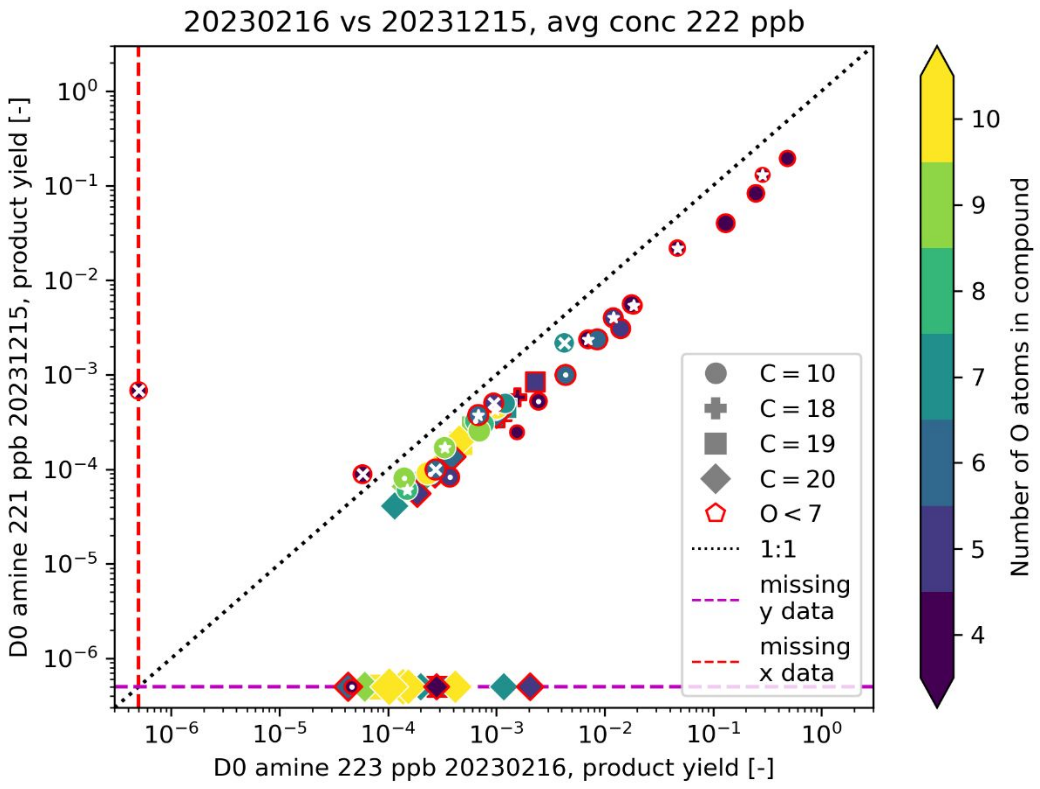

20230216 vs 20231215, avg conc 222 ppb
D0 amine 221 ppb 20231215, product yield [-]
D0 amine 223 ppb 20230216, product yield [-]
C = 10
C = 18
C = 19
C = 20
O < 7
1:1
missing y data
missing x data
Number of O atoms in compound

C

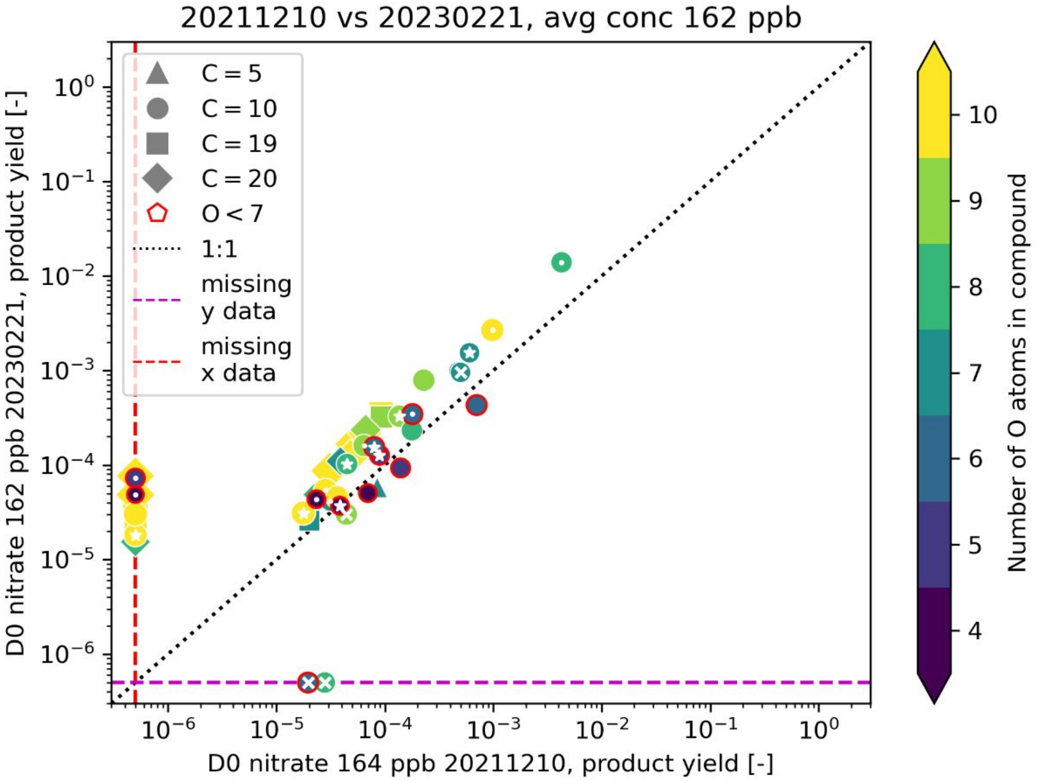
20211210 vs 20230221, avg conc 162 ppb
C = 5
C = 10
C = 19
C = 20
O < 7
1:1
missing y data
missing x data
D0 nitrate 162 ppb 20230221, product yield [-]
D0 nitrate 164 ppb 20211210, product yield [-]
Number of O atoms in compound
10
9
8
7
6
5
4

D

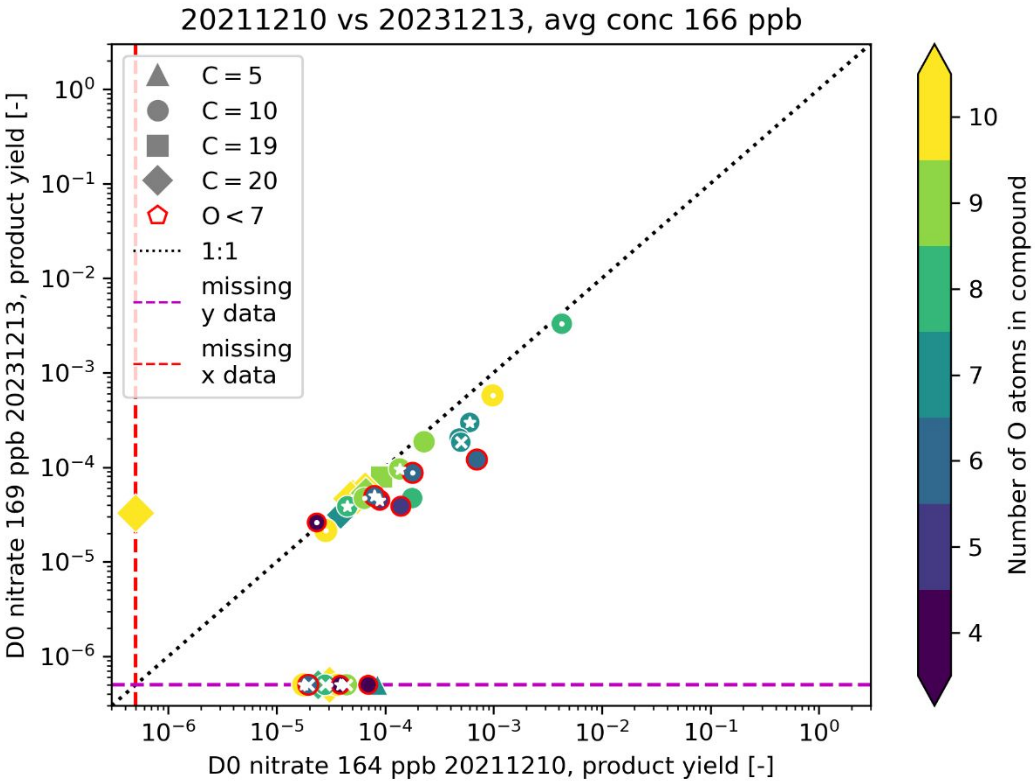

20211210 vs 20231213, avg conc 166 ppb
C = 5
C = 10
C = 19
C = 20
O < 7
1:1
missing y data
missing x data
D0 nitrate 169 ppb 20231213, product yield [-]
D0 nitrate 164 ppb 20211210, product yield [-]
Number of O atoms in compound

E

F

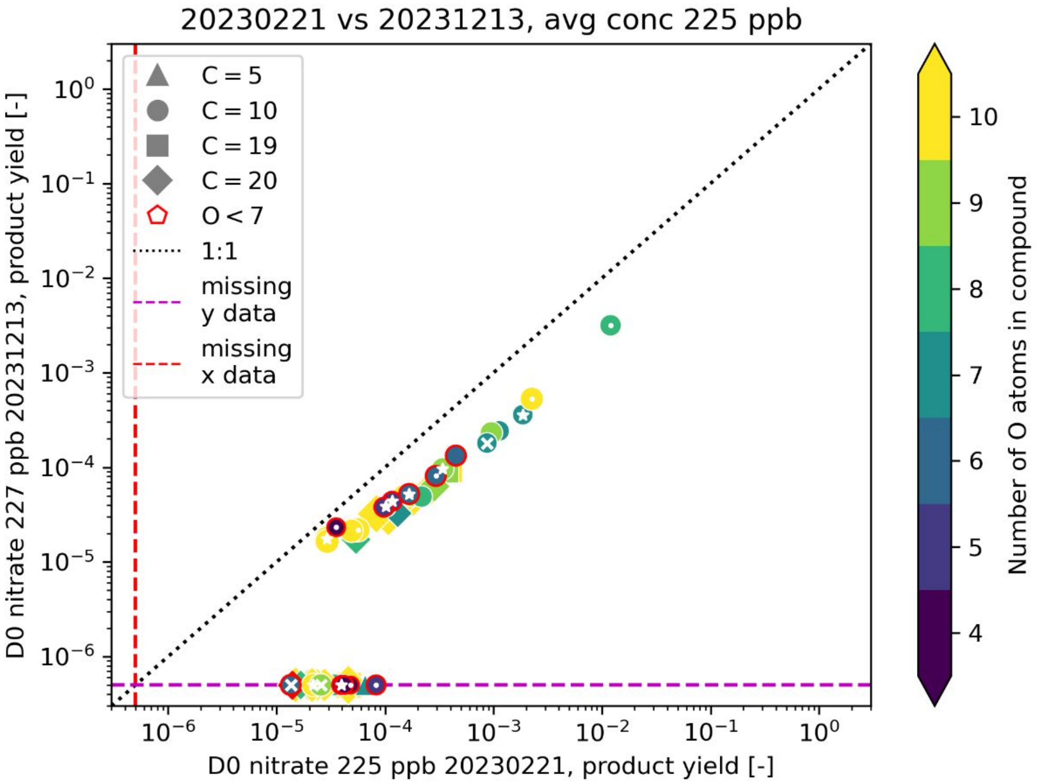
20230221 vs 20231213, avg conc 225 ppb
C = 5
C = 10
C = 19
C = 20
O < 7
1:1
missing y data
missing x data
D0 nitrate 227 ppb 20231213, product yield [-]
D0 nitrate 225 ppb 20230221, product yield [-]
Number of O atoms in compound

G

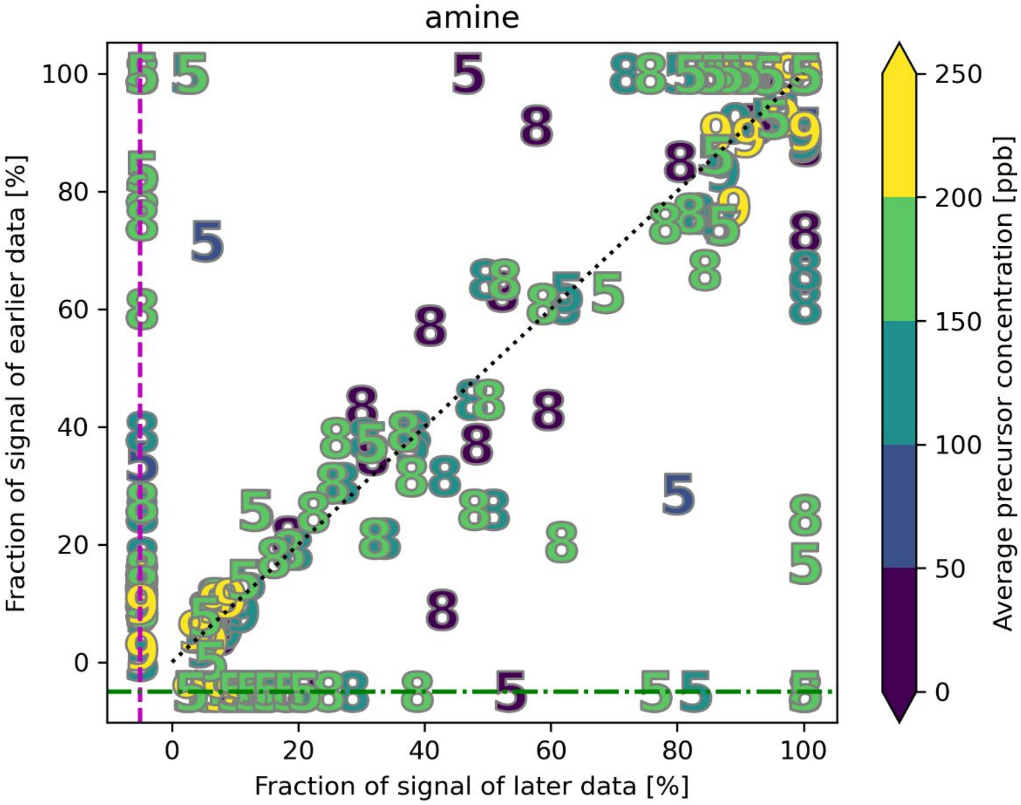
amine
Fraction of signal of earlier data [%]
Fraction of signal of later data [%]
Average precursor concentration [ppb]
0
20
40
60
80
100
0
50
100
150
200
250

H

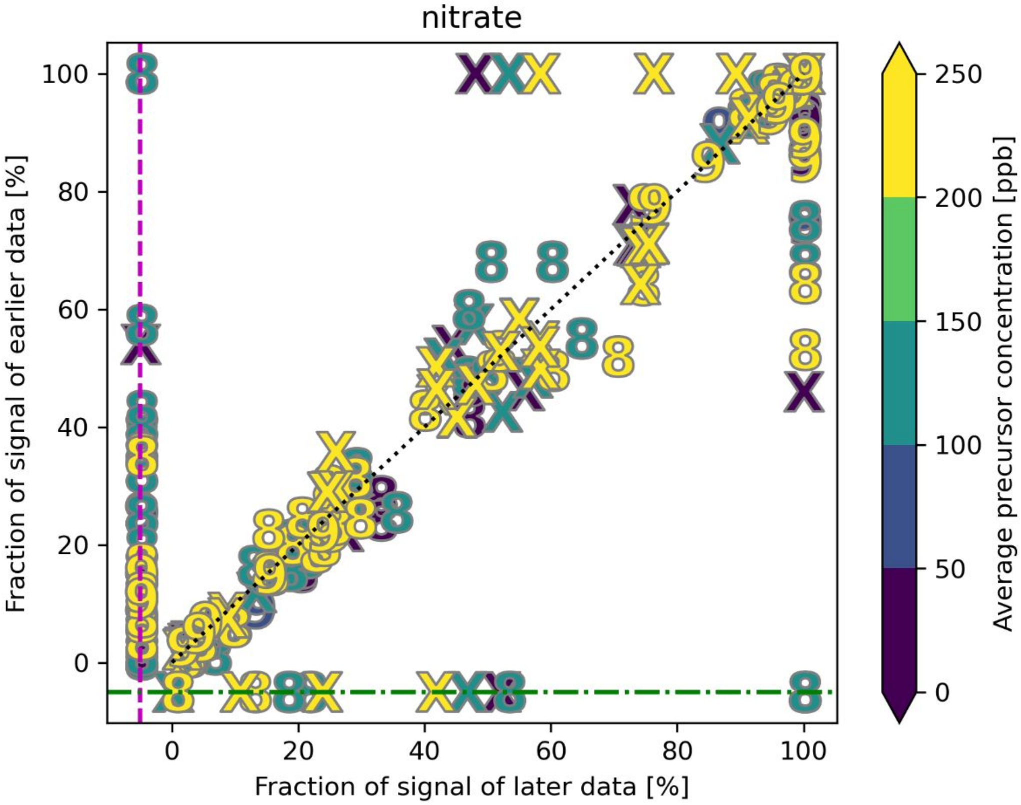
nitrate
Fraction of signal of earlier data [%]
Fraction of signal of later data [%]
Average precursor concentration [ppb]
0
20
40
60
80
100
0
50
100
150
200
250

Fig. S5. Comparison of product yields over different measurement years.

(**A-F**) Yields of oxidation products for later data against those for earlier data of non-labelled α-pinene (D0) ozonolysis. The color shows the number of oxygen atoms in the products. The products with less than 7 oxygen atoms are marked with red circles, and products $C_{10}H_{14}O_n$ are marked with a white star, $C_{10}H_{15}O_n$ with a white dot, and $C_{10}H_{17}O_n$ with a white cross. (**G, H**) Fractions of signals that have lost different numbers of deuterium atoms of earlier data against those of later data for nitrate (G) and N-butylaminium (H) experiments. The color shows the average precursor concentration in the flow reactor, and the marker shows the precursor used (5 = C5 precursor, 8 = C8 precursor, 9 = C9 precursor, X = C10 precursor).

S3. Pathways for α-pinene ozonolysis and autoxidation, and closed-shell product formation

The extended reaction mechanisms for α-pinene ozonolysis are shown in Fig. S6. The first steps of α-pinene ozonolysis include the formation of a primary ozonide and its isomerization to Criegee intermediates (CI), before forming the first-generation RO_2 with four oxygen atoms $C_{10}H_{15}O_4$ (Fig. S6A). Both the ozonide and the CIs can isomerize in different ways, and current knowledge suggests five different first-generation RO_2 resulting from the initial α-pinene and ozone reaction. [4–6] There are two O_4-RO_2 that undergo a C4 H-shift, and they are separated by whether they undergo a butyl ring breaking reaction (RO_2-I) or not (RO_2-Ka). The O_4-RO_2 can also be formed by undergoing a C10 H-shift (RO_2-Kb) or a C1 H-shift (RO_2-Kcd). Pathway IV can continue from the O_4-RO_2 to O_6-RO_2 via endoperoxide functionality formation and oxygen addition.[6] It can then undergo a H-shift from C3 and gain oxygen to form O_8-RO_2 (Fig. S6B).

The closed-shell species relevant to this study are formed either through unimolecular (R1)[7] or bimolecular (R2, R3) termination reactions[8,9] and the structures of the relevant species and their simplified formation mechanisms are shown in Fig. S7.

$$HOORO2^{\cdot} \rightarrow O = ROOH + OH^{\cdot} \quad (R1)$$

$$R'O2^{\cdot} + RO2^{\cdot} \rightarrow R'OH + R_{-H} = O + O_2 \qquad (R2)$$

$$R'O2^{\cdot} + RO2^{\cdot} \rightarrow R'OOR + O_2 \qquad (R3)$$

In the unimolecular case, a peroxy radical with a hydroperoxide (OOH) group undergoes a H-shift from the carbon with the OOH group, shifting the H to the OO* group. Then the OOH group connected to the carbon from which the H-shift took place forms a ketone or aldehyde group R=O and OH is lost, resulting in the chemical composition $C_{10}H_{14}O_{n-1}$ (R1), where the molecule has undergone a loss of H (H-loss). Note that while this reaction is a propagation reaction in the general radical kinetics sense, it is nevertheless usually called a "termination" in the context of aerosol-relevant oxidation chemistry as the large organic reactant has lost its radical center.

In bimolecular termination, O_n-RO_2 can react with another O_m-RO_2 forming an alcohol ROH with chemical composition $C_{10}H_{16}O_{m-1}$, a ketone or aldehyde R=O with chemical composition $C_{10}H_{14}O_{n-1}$ and oxygen O_2 (R2). The $C_{10}H_{14}O_{n-1}$ products have again undergone H-loss, and the H lost from the RO_2 forming the R=O comes from the carbon that had the OO* functionality.

O_n-RO_2 can also terminate bimolecularly by reacting with another O_m-RO_2 forming accretion products ROOR with chemical composition $C_{10}H_{30}O_{n+m-2}$ and O_2 (R3). Additionally, the mechanism for the formation of accretion products that have lost a carbon resulting in chemical formula $C_{19}H_{28}O_{4+m-3}$ is shown in Fig. S6C.[10] In this mechanism, the peroxy radical RO_2-Kb reacts with O_m-RO_2 forming O_2 and fragmenting CH_2O from RO_2-Kb. The fragmented carbon had the OO* group, i.e., C10 is fragmented. This reaction can take place between two RO_2-Kb where the CH_2O can be fragmented from both or one of the RO_2-Kb resulting in accretion products $C_{18}O_{26}O_4$ and $C_{19}H_{28}O_5$, respectively. Alternatively, two RO_2-Kb can undergo the R3 accretion reaction forming ROOR $C_{20}H_{30}O_{6.}$

The peroxy radicals formed from α-pinene ozonolysis have an even number of oxygens in them. As a result, the closed-shell products $C_{10}H_{14}O_n$ always have an odd number of oxygen atoms. Additionally, the accretion products with 20 carbon atoms always have an even number

of oxygen atoms $C_{20}H_{30}O_{even}$, and the accretion products with 19 carbon atoms have an odd number of oxygen atoms $C_{19}H_{28}O_{odd}$.

Noteworthy, the closed-shell product $C_{10}H_{14}O_7$ formed through unimolecular termination of O_8-RO_2 shown in Fig. S7 can be effectively achieved also through the bimolecular termination reaction R2. The difference would be that the lost H would originate from C4 instead of C3, and the H in the OOH group would be from C3 instead of C4. These products are indistinguishable with the selective deuteration used here, because both D from C3 and C4 would be lost as they would end up in OOH groups and would be exchanged for ^{1}H.

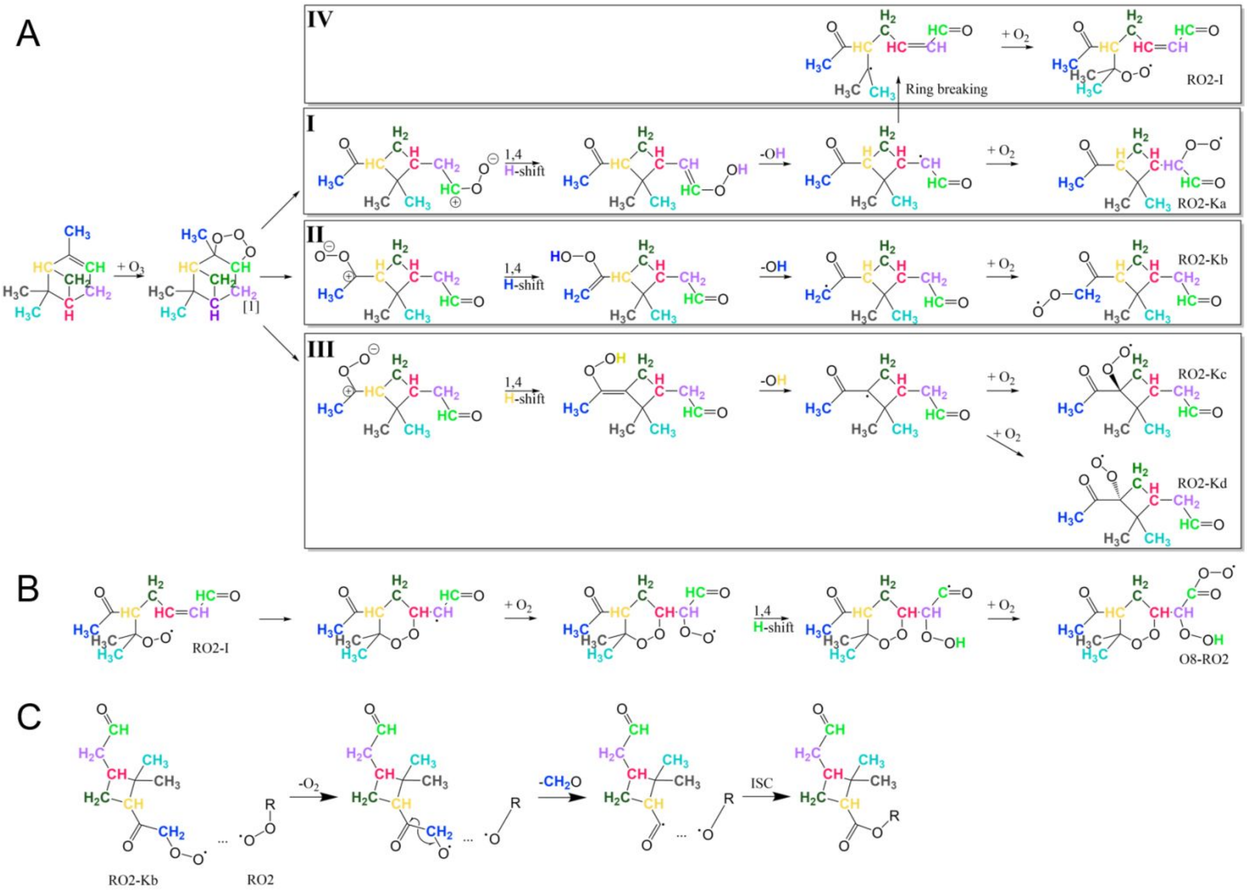

Fig. S6. α-pinene ozonolysis and autoxidation reactions

Computationally studied α-pinene ozonolysis pathways with carbons and hydrogens colored based on which selectively deuterated precursor they correspond with. (**A**) Pathways I-III[4], PIV[6], (**B**) continuation of PIV to O_8-RO_2, and (**C**) Reaction pathway for forming $C_{19}H_{28}O_n$ products from RO_2-Kb and RO_2 [10]. A and B are adapted from Meder et al. (2023)[1] under CC BY 4.0 license (https://creativecommons.org/licenses/by/4.0/), copyright 2023 Meder et al. (2023). C is adapted from Peräkylä et al. (2023)[10] copyright 2023 American Chemical Society.

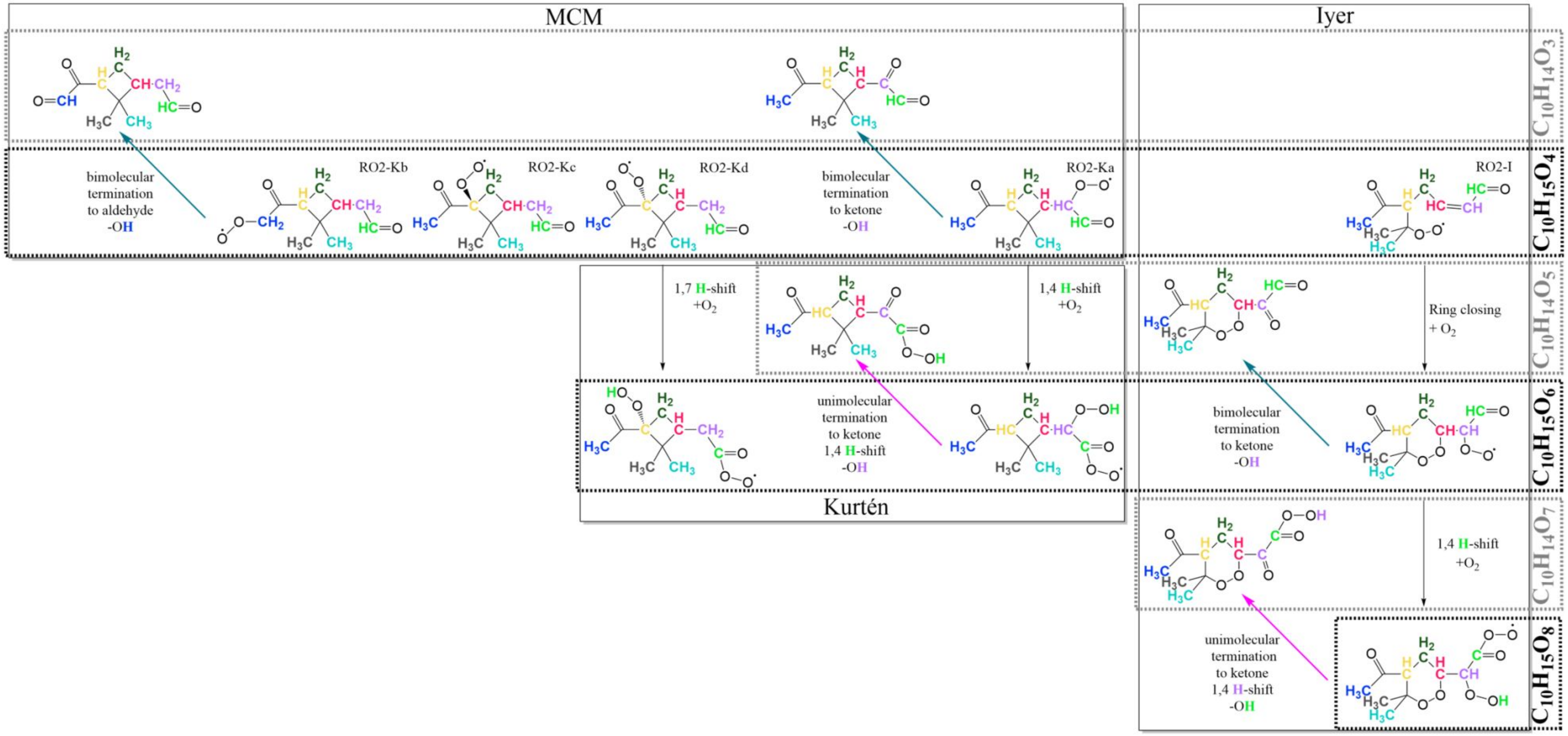

Fig. S7. Unimolecular and bimolecular termination reactions

Unimolecular and bimolecular termination reactions for α-pinene ozonolysis peroxy radicals RO_2 forming closed-shell products $C_{10}H_{14}O_{odd}$. Unimolecular termination reactions are shown with pink arrows and bimolecular termination reactions are shown with teal arrows.

S4. C8 precursor contamination determination

The spectra from C8 precursor oxidation differs significantly from every other precursor, showing significant increase in the relative yields of $C_{10}H_{14}$ products and producing dimers $C_{20}H_{28}$ that were not seen at such a rate with non-labelled α-pinene (e.g. Figs. S23-25). We found the reason to this behavior to be a contamination of $C_{10}H_{12}D_2$ that is likely formed during the synthesis of the deuterated precursor. The contamination could be seen in C8 precursor Vocus spectrum, however, no similar signal could be detected in C9 or non-labelled precursor spectra (Fig. S8A-C). Additionally, the contamination signal correlates with the injected precursor signal, implying the contamination comes from the sample (Fig. S8D-I). Furthermore, a small signal corresponding to the suspected $C_{10}H_{12}D_2$ could be seen in the ^{1}HNMR analysis of the precursor.

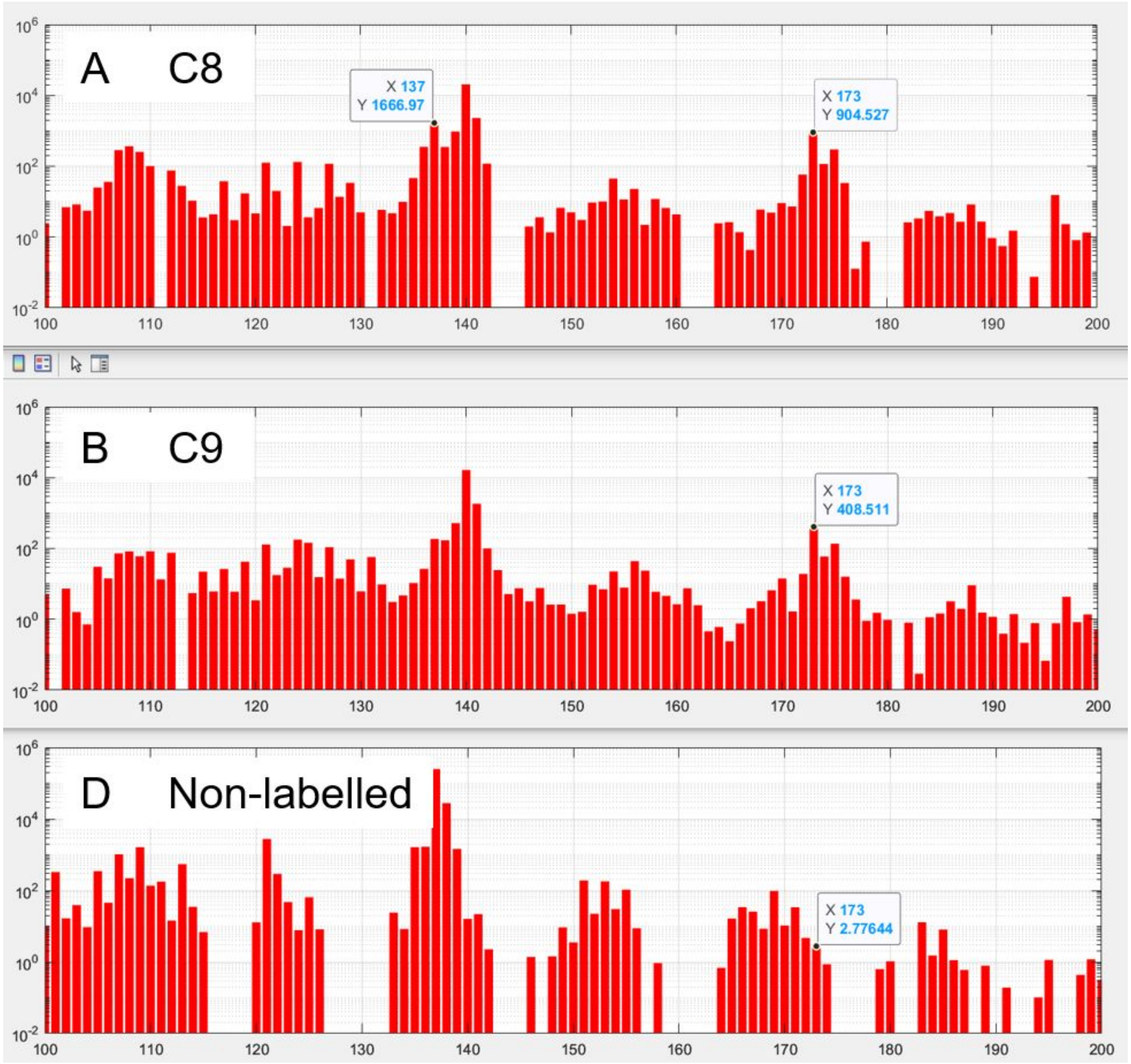

A C8
X 137
Y 1666.97
X 173
Y 904.527
B C9
X 173
Y 408.511
D Non-labelled
X 173
Y 2.77644

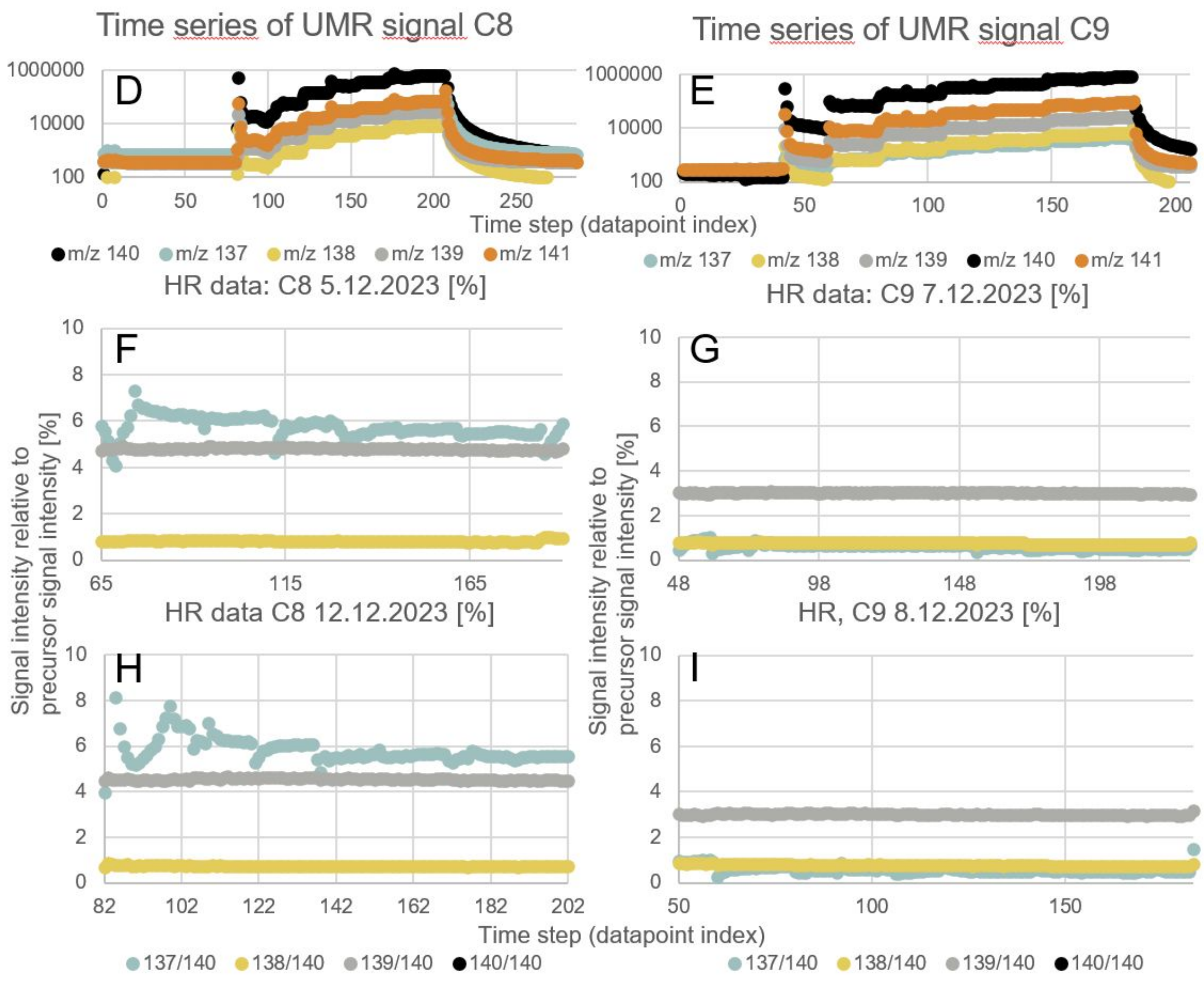

Fig. S8. C8 precursor contamination determination.

The unit mass resolution (UMR) mass spectra for (**A**) C8 precursor, (**B**) C9 precursor, and (**C**) non-labelled α-pinene. The time series of UMR data for (**D**) C8 precursor, and (**E**) C9 precursor. The time series of the signal intensities relative to the (**F**) C8 precursor signal on 5th of Dec 2023, (**G**) C9 precursor signal on 7th Dec 2023, (**H**) C8 precursor signal on 12th Dec 2023, and (**I**) C9 precursor signal on 8th Dec 2023. The values are measured with the Vocus PTR-ToF.

S5. Quantum chemical calculations on $C_{10}H_{16}O_3$

Quantum chemical methods were used to study the fates of the four possible Criegee intermediates from α-pinene ozonolysis shown in Fig. S9. Three possible pathways were considered, 1) 1,4 H-shift that leads to a vinyl hydroperoxide, 2) isomerization to a secondary ozonide (SOZ) and 3) isomerization to a dioxirane. The calculated energy barriers are shown in Table S1.

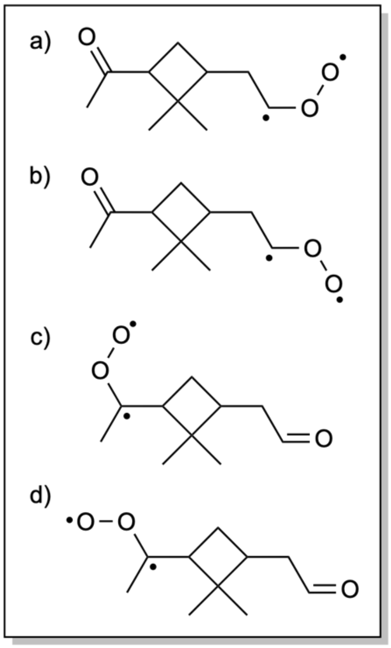

Fig. S9. Criegee intermediates from α-pinene ozonolysis.

Table S1.

Transition state (TS) energies of the three unimolecular reactions of Criegee intermediates. The energies were computed at the RHF-RCCSD(T)-F12a/VDZ-F12//ωB97X-D/aug-cc-pVTZ level of theory.

Isomer	TS kcal/mol		
	1,4 H-shift (VHP)	SOZ	Dioxirane
a)	15.9	15.4	21.6
b)	-	18.2	14.9
c)	16.7	13.8	19.9
d)	15.9	32.9	20.9

For CIs a) and c), the formation of SOZ proceeds at similar or even faster rates than the 1,4 H-shift that leads to VHP, indicating significant SOZ yields through these pathways. Once formed, SOZ are likely thermally stable and measured by the employed CIMS method as $C_{10}H_{16}O_3$.

CI d) has a significantly lower barrier for the VHP pathway compared to the other two and likely the sole thermal fate of the CI. Residual excess energy could lead to a non-negligible yield of dioxirane. Considering the high barrier, SOZ is unlikely to form along this pathway.

Finally, CI b) has no 1,4 H-shift pathway available to it and is likely to lead almost entirely to dioxirane, which was previously considered to rapidly convert to pinonic acid. The mechanism to form a carboxylic acid such as pinonic acid from a dioxirane involves two transition states (TS), an initial TSa that breaks the O---O bond of the dioxirane to form a diradical, and a second TSb that involves an H-shift to form the acid (see Fig. S10).[11] TSa was calculated at the ωB97X-D/aug-cc-pVTZ level of theory and found to be significantly high at 33.5 kcal/mol. This was recomputed using a multireference method at the XMC-CASPT2(6,4)/svp level of theory and found to be 24 kcal/mol. This indicates that the dioxirane is thermally stable and unlikely to rapidly convert to pinonic acid. We do note that if the dioxirane retains some of the excess energy from its formation, there can be a non-negligible yield of the diradical.

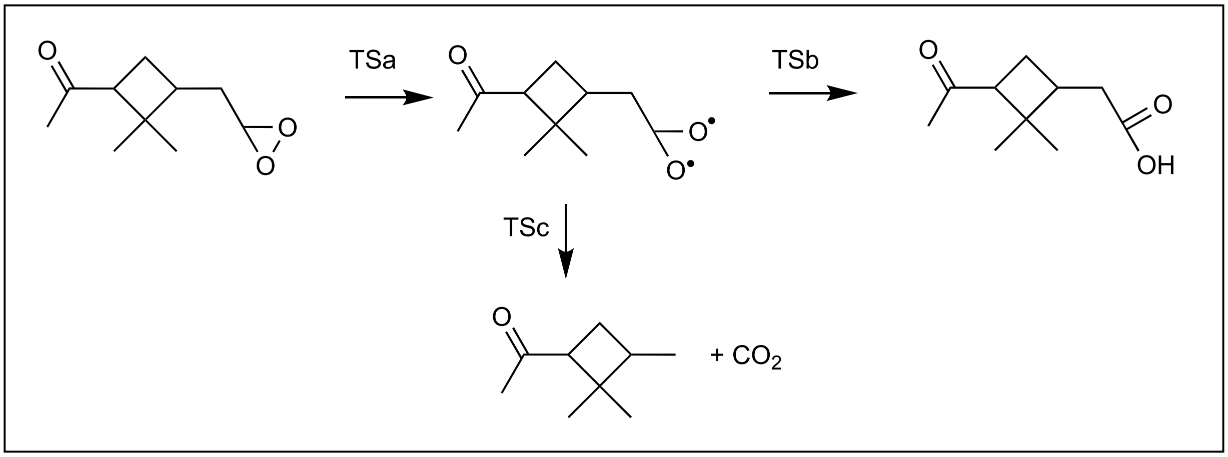

Fig. S10. Isomerization reactions of dioxirane.
The dioxirane initially forms a diradical via TSa and subsequently either a carboxylic acid or a CO_2 loss product via TSb and TSc, respectively.

The fate of the diradical was also studied, but DFT calculations were unable to find TSb leading to pinonic acid. A different TSc was found instead that lead to CO_2 loss and a C_9 product. Considering the likely strong multireference character of the diradical reactant, single-reference methods are unreliable. Multireference calculations (XMC-CASPT2 with 8 electrons in 7 MOs and tzvpp basis set) were carried out on a significantly smaller methyl-dioxirane test system. At this level of theory, TSc corresponding to the methyl-dioxirane system was found to be only 2.2 kcal/mol above the methyl-diradical intermediate, indicating prompt CO_2 loss. We were unable to find TSb despite more accurately treating the multireference system, so we cannot be certain that CO_2 loss is the principal fate of the methyl-diradical. We also note that we cannot be certain that the actual barrier for CO_2 loss in the α-pinene system is similarly low as the small test system. However, when juxtaposed with our measurements, this result is a strong indicator of the absence of a unimolecular pathway to pinonic acid from α-pinene ozonolysis.

These calculations indicate that the $C_{10}H_{16}O_3$ signal measured from our α-pinene ozonolysis experiments is most likely from SOZ and/or dioxirane.

S6. Carbons partaking in H-shifts in accretion product formation

The carbon positions that participated in H-shifts in the formation of the observed RO_2 also partake in the formation of the observed accretion products (Fig. S11), namely C4, C10, and C3. Additionally, we see some signal where carbon specific H-shifts from C1, C5 and C7 have taken place. To make our inspection more straightforward, we can focus on products whose forming reactants are possible to narrow down to two chemical formulae i.e. one for each reactant. These are $C_{20}H_{30}O_{4<m<10}$ and $C_{19}H_{28}O_n$. The former are known to form from two RO_2 losing O_2 in the process, thus we know that the reactants forming $C_{20}H_{30}O_6$ are $C_{10}H_{15}O_4$ and $C_{10}H_{15}O_4$, and the reactants forming $C_{20}H_{30}O_8$ are $C_{10}H_{15}O_4$ and $C_{10}H_{15}O_6$. As for the $C_{19}H_{28}O_n$, we found that these accretion products always lost C10 (Fig. S12D), which indicates that these products are formed via the mechanism shown in Fig. S6C (30). In other words, one of the reactants forming the $C_{19}H_{28}O_n$ products is always the RO_2-Kb with four oxygen atoms. This means that all the other signals can be attributed to the non-RO_2-Kb partner. Because in total 3 oxygen atoms are lost in the formation reaction, we can deduce that the reaction partners when forming $C_{19}H_{28}O_n$ are RO_2-Kb and $C_{10}H_{15}O_{n-1}$.

Let us start from the $C_{20}H_{30}O_m$ accretion products. C4 and C10 H-shifts explain most of the $C_{20}H_{30}O_6$ signal, i.e. $C_{20}H_{30}O_6$ could be formed from O_4-RO_2 where C4 H-shift has taken place and O_4-RO_2 where C10 H-shift has taken place (Fig. S11B). However, the signal that has C10 participating in H-shifts consists of signal that has lost two D, meaning that two C10 H-shifts have had to take place (Fig S12). Additionally, around a third of the signal where C4 is participating in H-shifts has had two D lost, meaning again that two C4 H-shifts have had to take place. One way to explain this is to have the part of the signal be formed from two O_4-RO_2 that have undergone one C4 H-shift each, and part of the signal formed from two O_4-RO_2 that have undergone one C10 H-shift each. Thus, we could explain around 70 % of the $C_{20}H_{30}O_6$ signal with RO_2-Kb and RO_2-I/Ka. The rest of the signal could be explained by RO_2-I/Ka reacting with O_4-RO_2 that has undergone a C5 or C9 H-shift, however, the signal fractions corresponding to C5 and C9 H-shifts are so small that they have very high uncertainties.

As for $C_{20}H_{30}O_8$, which should be formed from $C_{10}H_{15}O_4$ and $C_{10}H_{15}O_6$, only C4 H-shifts have taken place. Around half of the signal has had two D lost, meaning that two C4 H-shifts have had to take place. With the same logic applied previously, we can explain around 50 % of the $C_{20}H_{30}O_8$ signal with RO_2-Ka/I reacting with O_6-RO_2 from the Iyer mechanism (O_6-RO_2-I). We could explain the rest of the $C_{20}H_{30}O_8$ signal by one reaction partner being either RO_2-Ka/I or O_6-RO_2-I, but the other reaction partner remains unknown. If we consider the kinetic isotope effect (KIE) and that the signal for $C_{20}H_{30}O_8$ is completely missing from the C10 deuterated precursor, it is possible that the other reaction partner has undergone C10 H-shift, but we can only speculate. C3 can be ruled out as a potential partner despite it also having KIE influence its signals as the total signal for $C_{20}H_{30}O_8$ is not significantly decreased compared to the that of non-labelled α-pinene spectrum and we still do not see signal for D-loss.

Moving to the $C_{19}H_{28}O_n$ species, the $C_{19}H_{28}O_5$ signal shows that C4, C5 and C10 H-shifts have taken place during the formation reactions. The C10 signal shows that around half the signal has four D lost, implying that both reactants are RO_2-Kb 50 % of the time. The other half of the signal can be from reactions between RO_2-Kb and RO_2 that has had C4 and C5 H-shifts take place. Thus, around a third of the signal could be RO_2-Kb reacting with RO_2-Ka/I.

Noteworthy, $C_{19}H_{28}O_5$ data imply the formation of O_4-RO_2 where C5 H-shifts take place though at low amounts, as was the case with $C_{20}H_{30}O_6$.

Due to the low signal of $C_{19}H_{28}O_7$, we cannot say what reactants usually form it. The $C_{19}H_{28}O_9$ data show that RO_2 other than O_8-RO_2-I is formed, even though the O_8-RO_2 signal could be explained fully by O_8-RO_2-I. When $C_{19}H_{28}O_9$ is formed, one of the reaction partners is always RO_2-Kb that has lost C10 and the other reactant is O_8-RO_2. The accretion product signal shows that C4 has undergone a H-shift whereas C3 never has, meaning that the O_8-RO_2 signal cannot be explained solely by the Iyer pathway. Additionally, around a fifth of the signal has had two C4 H-shifts take place, and to a lesser extent also a C1 H-shift has taken place.

When we account for the RO_2-Kb signal in $C_{19}H_{28}O_{11}$, the unaccounted signal follows a similar pattern of behavior as the observed O_{10}-RO_2, with some increase in C1,and C3 specific H-shift fractions.

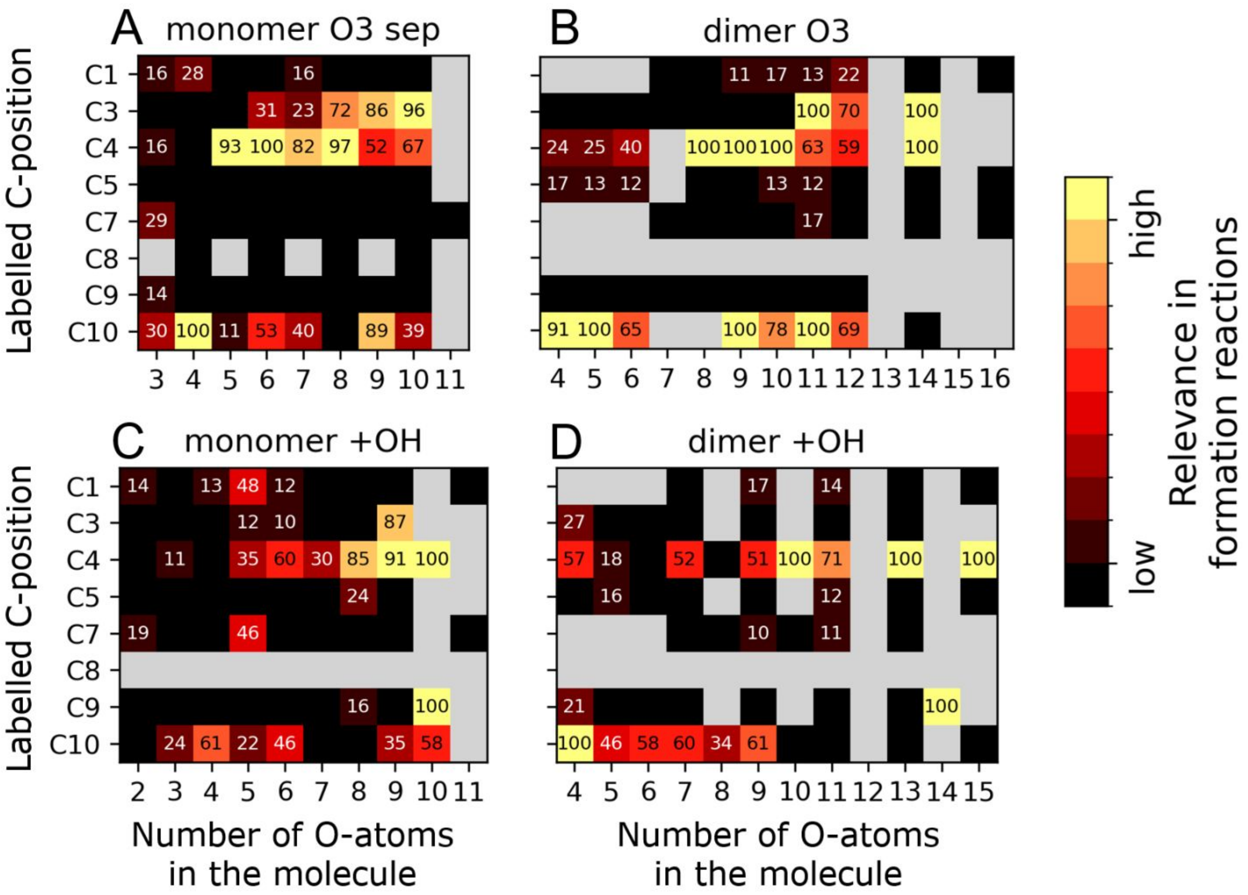

Fig. S11. Heatmap of relevance of H-shifts

Heatmap of relevance of H-shifts from certain carbons in the α-pinene molecule as a function of number of oxygen atoms in the products. The products are grouped into (**A**) monomers formed through α-pinene ozonolysis oxidation $C_{10}H_{15}O_{even}$, $C_{10}H_{14}O_{odd}$, (**B**) accretion products formed through α-pinene ozonolysis oxidation $C_{18}H_{26}O_4$, $C_{19}H_{28}O_{odd}$, $C_{20}H_{30}O_{even}$, (**C**) monomers that can be formed through α-pinene ozonolysis oxidation and OH oxidation $C_{10}H_{16,17}O_n$, $C_{10}H_{14}O_{even}$, $C_{10}H_{15}O_{odd}$ and (**D**) accretion products that are formed through α-pinene ozonolysis oxidation and OH oxidation $C_{20}H_{28,32}O_n$, $C_{20}H_{30}O_{odd}$, $C_{19}H_{28}O_{even}$. The monomer values are averaged over experiments with $3.3 \cdot 10^8$ cm^{-3} to $2.1 \cdot 10^9$ cm^{-3} reacted precursor, and accretion product values are averaged over experiments with reacted precursor $\geq 3.3 \cdot 10^9$ cm^{-3}. The color shows the fraction of signal that has lost at least one D corrected for the KIE, i.e., the relevance of the H-shifts and H-loss from the specific carbon in the formation reaction of the products.

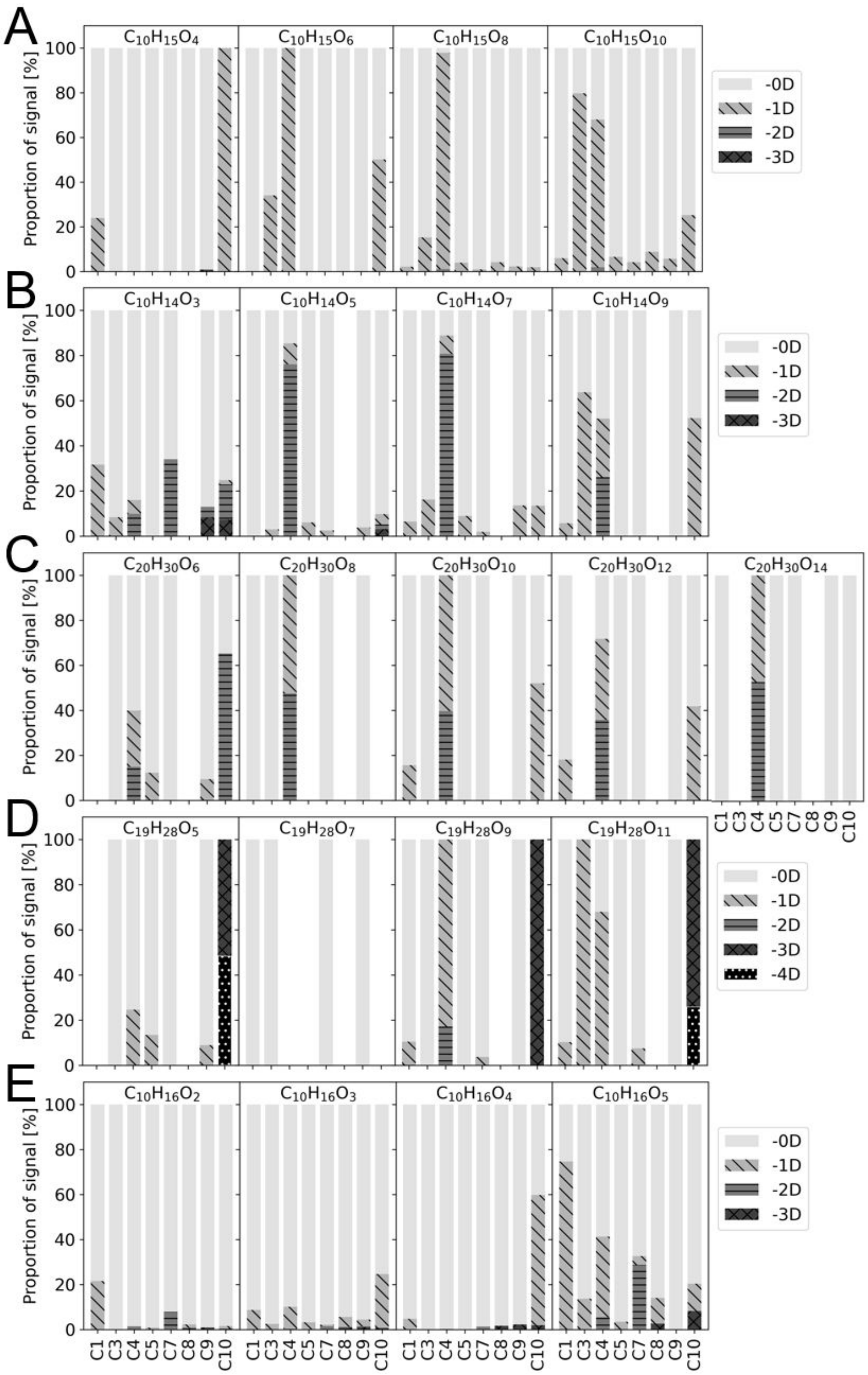

Fig. S12. Signal distributions

Observed signal distributions between isomers for each deuterated precursor. The hatching and coloring show the number of deuterium atoms lost from the products. (**A**) Products $C_{10}H_{15}O_{even}$ at $2.1 \cdot 10^9$ cm^{-3} reacted precursor, (**B**) $C_{10}H_{14}O_{odd}$ at $2.1 \cdot 10^9$ cm^{-3} reacted precursor, (**C**) $C_{20}H_{30}O_{6,\ 8,\ 10,\ 12,\ 14}$ at $3.6 \cdot 10^9$ cm^{-3} reacted precursor, (**D**) $C_{19}H_{28}O_{5,7,9,11}$ at $3.6 \cdot 10^9$ cm^{-3} reacted precursor, and (**E**) $C_{10}H_{16}O_{2\text{-}5}$ at $8.2 \cdot 10^8$ cm^{-3} reacted precursor.

S7. Kinetic modeling: comparison of linear and non-linear models

We used a simple 0D kinetic model to probe the behavior of cyclohexene (CH) autoxidation under the conditions depicted in Figure 8 in Berndt et al. (2015).[12] We tested two different approaches, the "linear" and the "non-linear" case. In both cases, the reaction of CH and O_3 forms the first-generation RO_2 radicals with composition $C_6H_9O_4$. In the linear case, 5% (roughly corresponding to the HOM yield for this system) of the O_4-RO_2 can undergo autoxidation to form the O_6-RO_2, which, in turn, undergoes autoxidation to the O_8-RO_2, which can form the final O_{10}-RO_2. The top panels of Fig. S13 depict the results of this linear case with different rate coefficients (k_autox = 0.1, 1, or 50 s^{-1}). For simplicity, this rate was identical for each autoxidation step, i.e. O_4->O_6 and O_8->O_{10} occurred at the same rates. This is unlikely to be the case in reality, but the results already clearly show that observations (depicted by crosses) cannot be reproduced with this approach. If focusing on the fact that experiments show that O_8-RO_2 is much more abundant than the O_6-RO2, the rates forming the O_8-RO_2 cannot be too low (e.g. Fig. S13A), but at higher rates, the O_6-RO_2 loss rate becomes so high that the concentration plateaus after a few seconds, unlike the observations. In the "non-linear" case, we assume that four different types of O_4-RO_2 are formed. As in the linear case, 95% forms an O_4-RO_2 that cannot undergo autoxidation, while the three other types can undergo autoxidation to different RO_2 before forming a radical that cannot undergo anymore autoxidation: the first can undergo exactly one autoxidation step to form O_6-RO_2, the second can undergo two steps to first form O_6-RO_2 and then the O_8-RO_2, while the third can undergo three steps to form O_6, O_8, and O_{10}-RO_2. The approach is able to reproduce the continuous increasing trend of the observations, since each RO_2 has one isomer that can accumulate. The yields of each type of O_4-RO_2 were chosen to fit the observations and were 95%-0.16%-4.8%-0.04%. With these values, and a very high assumed autoxidation rate, the results can be reproduced very well (Fig. S13F). We acknowledge that these rates of 50 s^{-1} are much higher than typically predicted by QC, but as also noted by Berndt et al. (2015), the appearance of $C_6H_9O_{10}$ within seconds means that the effective autoxidation rates much be higher than 1 s^{-1}.[12]

We chose to model the CH case as it had data for each of the different RO_2, but a similar type of figure was published by Berndt et al. (2018) Figure S9[13], for the case of α-pinene, though with only the O_4 and O_8 radicals depicted. However, the study shows that all the O_6, O_8, and O_{10}-RO_2 radicals are roughly equal in abundance, and thus very similar behavior is expected as in the CH case.

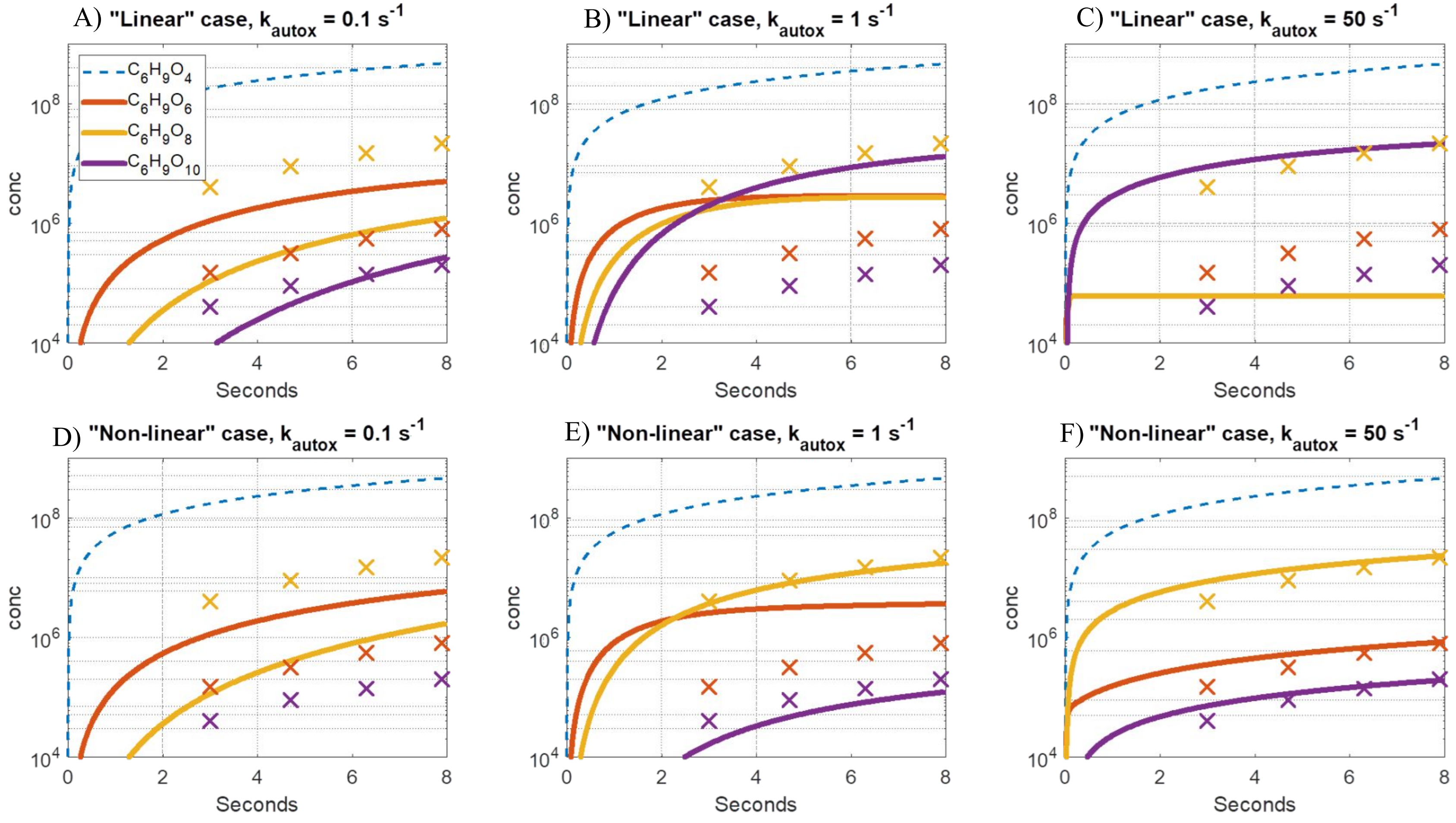

Fig. S13. Comparison of linear and non-linear models for cyclohexene autoxidation.

(**A-C**) Linear models using and (**D-F**) non-linear models using a slow autoxidation reaction rate (A, D), an autoxidation reaction rate competitive with bimolecular reactions (B, E), and a fast autoxidation reaction rate (C, F). The modelled values are shown with curves and the matching observations are shown with markers with matching colors.

S8. Data corrections

We corrected the Orbitrap data for three behaviors: 1. Decreasing sensitivity as signal intensity decreases[14], 2. relative ion transmission affecting signal intensities as a function of mass[14,15], and 3. fragmentation of $C_{10}H_{15}O_4$ signal in aminium ionization to $C_{10}H_{15}O_4$ and $C_{10}H_{15}O_2$ [16]. The correction functions for behavior 1 are shown in Fig. S14A-C and they are obtained from the distributions of natural isotopes of elements N, O, and C. The relative ion transmission correction function (Fig. S14D) is used before signal intensities are normalized with the total reagent ion signal. Behavior 3 is corrected by summing the signals of $C_{10}H_{15}O_4$ and $C_{10}H_{15}O_2$ and removing the $C_{10}H_{15}O_2$ signal from the inspection.

The sensitivity threshold of the CI-orbitrap can be determined from the sensitivity data (Fig. S14A-C) for nitrate and N-butylammonium separately. We use 20 % as the limit, meaning that the sensitivity threshold corresponds to the normalized signal intensity at which the measured abundance of a natural isotope is 20 % of the theoretical abundance. We normalize the signal intensities in Fig. S14A-C with the total reagent ion signal, which was roughly 2×10^6 cps in our experiments for both nitrate and N-butylammonium ionization. Thus, the sensitivity threshold of the nitrate CI-orbitrap is below 10^{-5} ncps, and the N-butylammonium CI-orbitrap is below 2.5×10^{-5} ncps. Noteworthy, the total reagent ion signal decreased significantly when using $> 2 \times 10^9$ cm^{-3} reacted precursor concentrations with N-butylammonium ionization which might have affected the sensitivity of the instrument.

We can compare our main radical signals from both ozonolysis and OH oxidation to those measured in another flow reactor set up with CI-API-ToF at atmospherically relevant conditions shown in Fig. S15.[13] Our experiments were conducted in conditions where the reacted precursor was higher than what is atmospherically relevant. Our estimated concentrations are in general lower and the distribution of the RO_2 differs from the previous study. The order of ozonolysis RO_2 from most abundant to least for our data is $C_{10}H_{15}O_{4,6,10,8}$ for aminium data and $C_{10}H_{15}O_{8,10,6,4}$ for nitrate data, whereas for the previous study utilizing aminium CI it is $C_{10}H_{15}O_{4,10,(8,6)}$. The difference in the order of the most abundant RO_2 is due to the different reagent ions being selective towards different compounds, as Berndt et al. 2018 utilized protonated N-propylamine $C_3H_7NH_4^+$ instead of N-butylaminium $C_4H_9NH_3^+$ and nitrate NO_3^- that we used.

When analyzing the D_2O experiment data, we need to account for the similar mass-to-charge ratio (m/z) between a product that has had 3 or more hydrogen atoms exchanged for deuterium atoms ($C_nH_mD_xO_y$) and a product with two more total number of hydrogen atoms, 3 less deuterium atoms and one carbon atom being replaced by its isotope ^{13}C ($C_{n-1}{}^{13}CH_{m+2+3}D_{x-3}O_y$). The m/z difference between $C_nH_mD_xO_y$ and $C_{n-1}{}^{13}CH_{m+2+3}D_{x-3}O_y$ is very small at -0.000175 Th, rendering the difference below the ± 1 ppm mass accuracy that the orbitrap reliably has. Additionally, the difference between the m/z of these two compounds is less than a fifth of the peak width ΔM (full width at half maximum) achieved with the 280 000 Th/Th mass-resolving power even for the lowest m/z compounds inspected, namely 270 Th.

$$m/z(C_nH_mD_xO_y) - m/z(C_{n-1}{}^{13}CH_{m+3+2}D_{x-3}O_y)$$
$$\Leftrightarrow m/z(CD_3) - m/z(^{13}CH_{3+2})$$
$$\approx 18.042305\,Th - 18.042480\,Th = -0.000175\,Th$$
$$\Delta M = \frac{M}{R} = \frac{270\,Th}{280\,000} \approx 0.000964\,Th$$

To account for this, we went through all peaks that were identified to potentially have 3 or more deuterium atoms and checked the spectrum to find the signal of the main isotope of the

corresponding ^{13}C product. If the main isotope was found and it was significant enough to explain half of the product signal, we considered that compound to be the ^{13}C product and excluded it from the inspection.

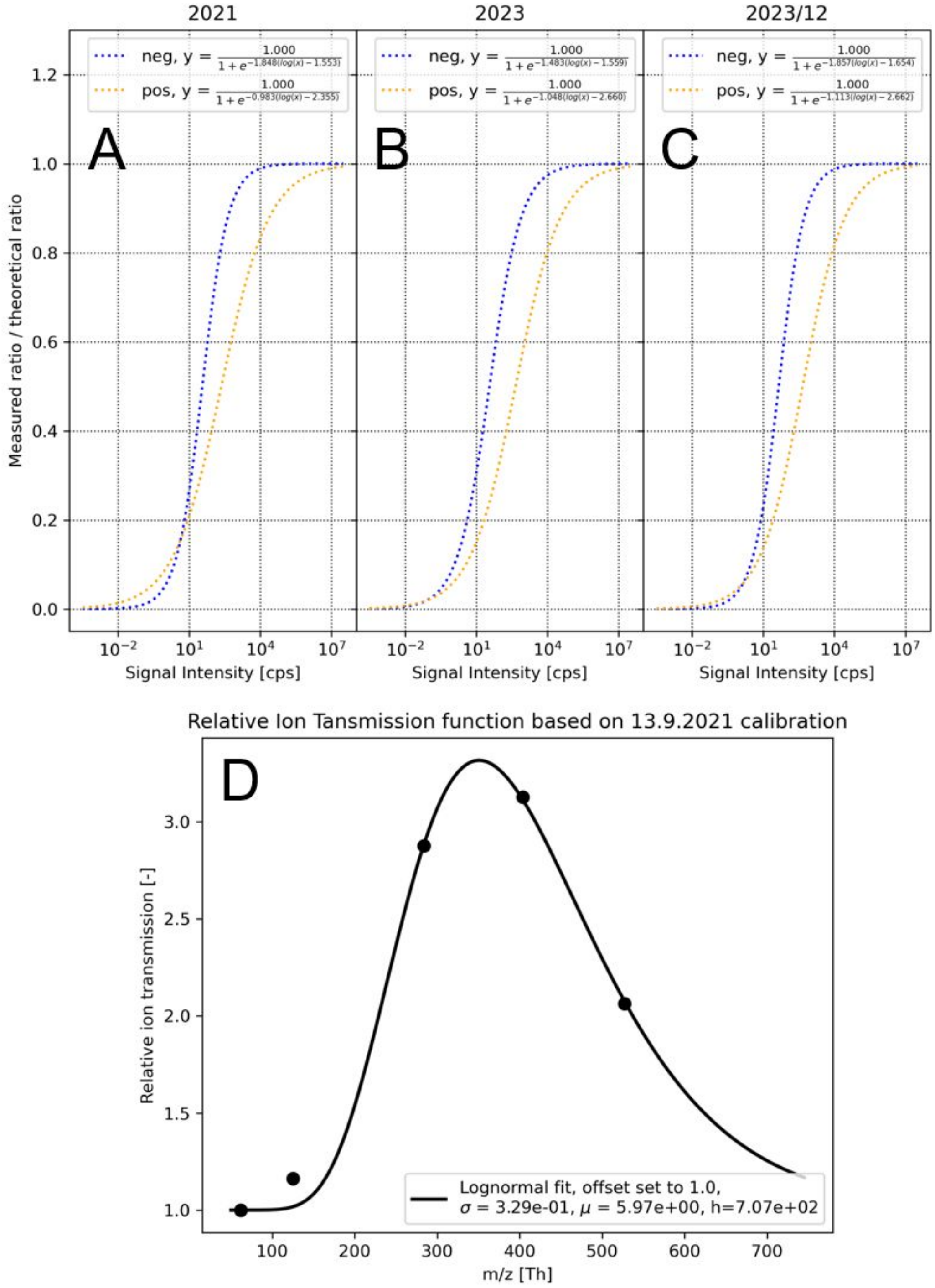

Fig. S14. Orbitrap data corrections

Data correction functions for correcting two of Orbitrap's behavior: 1. Sensitivity as a function of signal intensity where the sensitivity is acquired from natural isotope distributions of C, N, and O (**A**, **B**, **C**). 2. Relative ion transmission as a function of mass to charge ratio (**D**).

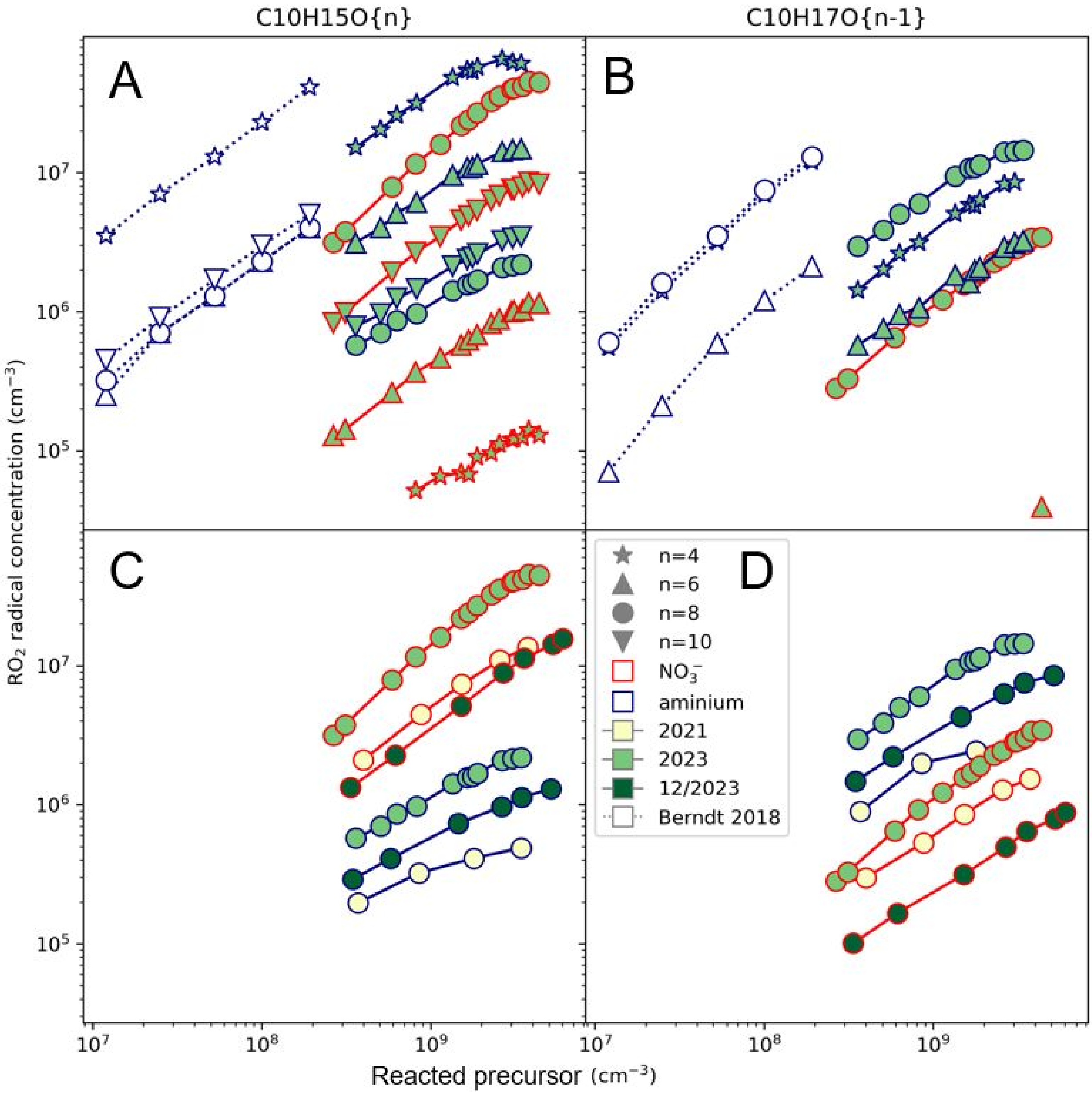

Fig. S15. Comparison to previously measured data

Comparison of our data to previously measured radical data adapted from Berndt et al. 2018. We used nitrate NO_3^- and N-butylaminium $C_4H_9NH_3^+$ ionization, and the previous experiment used protonated propylamine $C_3H_7NH_3^+$. Estimated RO_2 concentration as a function of reacted precursor. (**A, C**) Ozonolysis RO_2 and (**B, D**) OH oxidation RO2, where the markers highlighted in red are from nitrate data and markers highlighted in blue are from aminium data. The marker shows the number of oxygen atoms in the product, and the fill color tells the year the values were measured, showing Berndt et al. 2018 data with white fill color.[13]

S9. Yield calculations

Molar yields γ are estimated using the rate of change of product concentration [HOM] assuming there are no losses. The OH oxidation is not taken into account when calculating the yield resulting in an overestimation of the total yield. However, the yields from α-pinene ozonolysis for the $C_{10}H_{14}O_{odd}$, $C_{10}H_{15}O_{even}$ species are not overestimated as they are only formed through ozonolysis.

$$\frac{\Delta[HOM]}{\Delta t} = production - loss = \gamma k_1[O_3][\alpha p] - 0$$

$$\gamma = [HOM]/(k_1[O_3][\alpha p]\Delta t),$$

where $k_1 = 8.89 \times 10^{-17} cm^3 s^{-1}$ is the α-pinene ozonolysis reaction rate coefficient at 23 ^oC, $\Delta t \approx 3\ s$ is the reaction time in the flow reactor, and $[O_3]$ and $[\alpha p]$ are ozone and α-pinene or precursor concentrations, respectively.[17] The product concentration can be obtained by normalizing the product signal with total reagent ion signal and multiplying it with a calibration coefficient C. Here we use $C = 10^{10} cm^{-3}$ to get estimates for the product concentrations. Using this coefficient value brings significant additional uncertainties. However, when discussing the yields in the main text, we always use relative yield, i.e., the ratio of yields between a compound from a deuterated sample and a matching compound from non-labelled α-pinene, thus eliminating the uncertainty from the calibration coefficient. Nevertheless, this does not eliminate all uncertainties, and significant uncertainties remain in the data due to the nature of CIMS measurements and the lack of direct calibration. The yields of the selectively deuterated precursors plotted against matching products' yields from non-labelled α-pinene oxidation are shown in Figs S18-25 for all selectively deuterated precursors used.

$k_1[O_3][\alpha p]\Delta t$ is also known as the reacted α-pinene, or reacted precursor when deuterated precursors are used. We calculate this and the yields using measured values instead of initial values, because the amount of precursor that reacts is very small, roughly 0.066 % of the original concentration when estimating it by dividing the reacted precursor at 100 ppb ozone concentration with precursor concentration.

$$\frac{k_1[O_3][\alpha p]\Delta t}{[\alpha p]} = k_1[O_3]\Delta t \approx 0.00066 = 0.066\ \%$$

S10. Exchangeable hydrogens in HOMs

Hydrogen atoms in hydroxide and hydroperoxide groups are readily exchanged with deuterium in heavy water, hence we can study the formation of these groups through the exchangeable hydrogen atoms with hydrogen-deuterium exchange.[18]

The D_2O experiment results correspond with previously reported values, while adding detail to the inspection.[18] The eight-oxygen peroxy radical O_8-RO_2 $C_{10}H_{15}O_8$ has one exchangeable hydrogen atom (EH) which corresponds with the Iyer pathway O_8-RO_2, and O_{10}-RO_2 has mostly two (Fig. S16). The closed-shell products that are formed from the RO_2 (Fig. S7) have more variety in the number of these groups. Four fifths of $C_{10}H_{14}O_7$ signal have one exchangeable hydrogen which corresponds with the closed-shell product unimolecularly formed from Iyer O_8-RO_2, and one fifth of the signal has two EH. $C_{10}H_{14}O_9$ one fifth has one EH, half have two EH and one third have three EH. These distributions did not significantly change with increasing reacted precursor concentration, except for $C_{10}H_{14}O_7$ where the fraction that has one EH increased around 20 percentage points when the reacted precursor concentration increased from ~$4.8 \cdot 10^8$ cm^{-3} to ~$3.8 \cdot 10^9$ cm^{-3} (Fig. S16A). The values for compounds with under 7 oxygen atoms can be distorted by the fact that nitrate CIMS is sensitive toward higher oxygenated products. Additionally, nitrate CIMS sensitivity toward products with fewer EH decreases and is already poor towards compounds with under 2 EH. With these factors combined, it is known that the nitrate CIMS does not generally detect products with zero EH although they are certainly formed.

The number of EH for other oxidation products are shown in Fig. S16B-E, and the estimated concentrations for the inspected products are shown in Fig. S17.

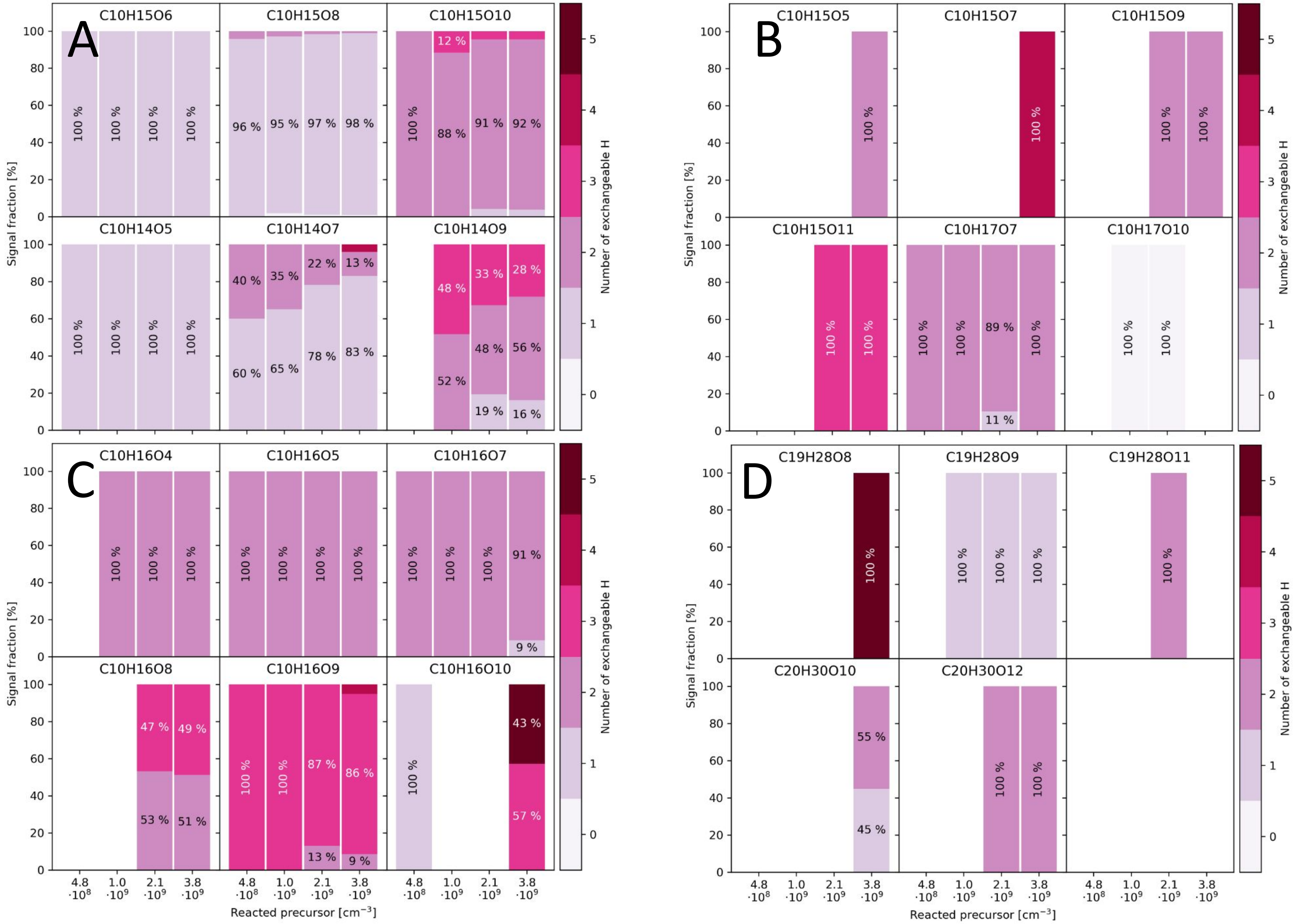

A
B
C
D
C10H15O6
C10H15O8
C10H15O10
C10H14O5
C10H14O7
C10H14O9
C10H15O5
C10H15O7
C10H15O9
C10H15O11
C10H17O7
C10H17O10
C10H16O4
C10H16O5
C10H16O7
C10H16O8
C10H16O9
C10H16O10
C19H28O8
C19H28O9
C19H28O11
C20H30O10
C20H30O12
Signal fraction [%]
Number of exchangeable H
Reacted precursor [cm^{-3}]

E

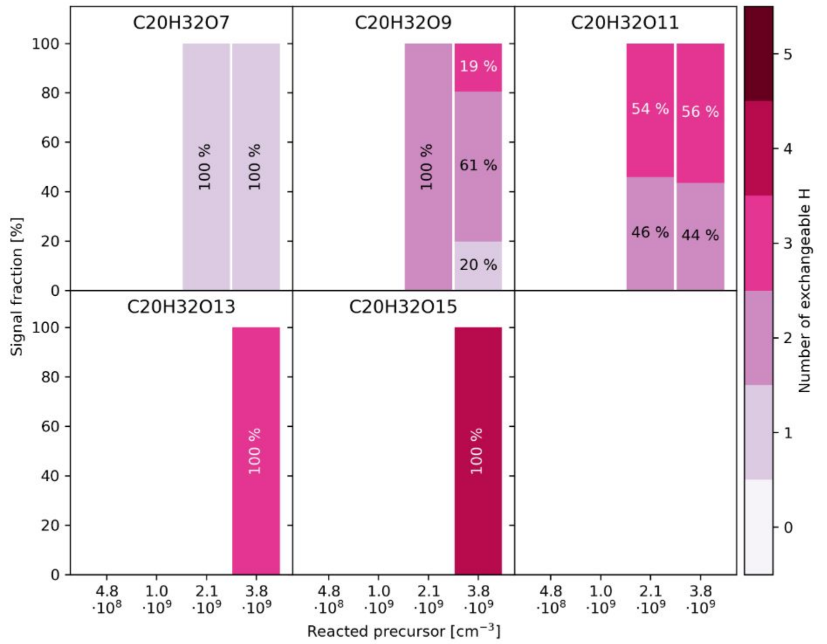

Fig. S16. Labile H distributions

Number of exchangeable hydrogens in α-pinene ozonolysis and OH oxidation products as a function of reacted α-pinene. (**A**) Products $C_{10}H_{15}O_{6,8,10}$ and $C_{10}H_{14}O_{5,7,9}$, (**B**) $C_{10}H_{15}O_{odd}$ and $C_{10}H_{17}O_{7,10}$, (**C**) $C_{10}H_{16}O_{4,5,7\text{-}10}$, (**D**) $C_{19}H_{28}O_{8,9,11}$ and $C_{20}H_{30}O_{10,12}$, and (**E**) $C_{20}H_{32}O_{odd}$.

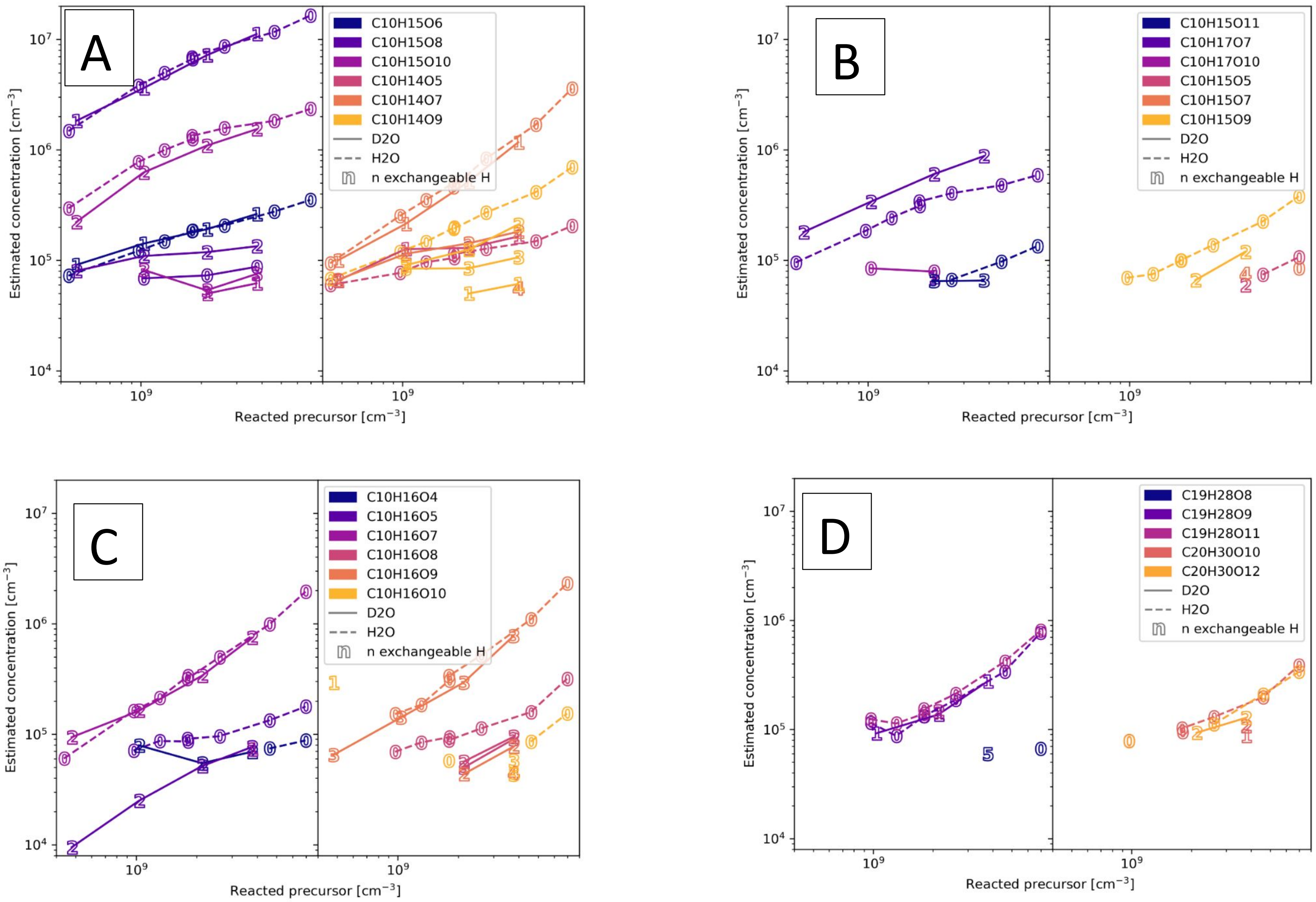
A
C10H15O6
C10H15O8
C10H15O10
C10H14O5
C10H14O7
C10H14O9
D2O
H2O
n exchangeable H
B
C10H15O11
C10H17O7
C10H17O10
C10H15O5
C10H15O7
C10H15O9
D2O
H2O
n exchangeable H
C
C10H16O4
C10H16O5
C10H16O7
C10H16O8
C10H16O9
C10H16O10
D2O
H2O
n exchangeable H
D
C19H28O8
C19H28O9
C19H28O11
C20H30O10
C20H30O12
D2O
H2O
n exchangeable H
Estimated concentration [cm^{-3}]
Reacted precursor [cm^{-3}]

E

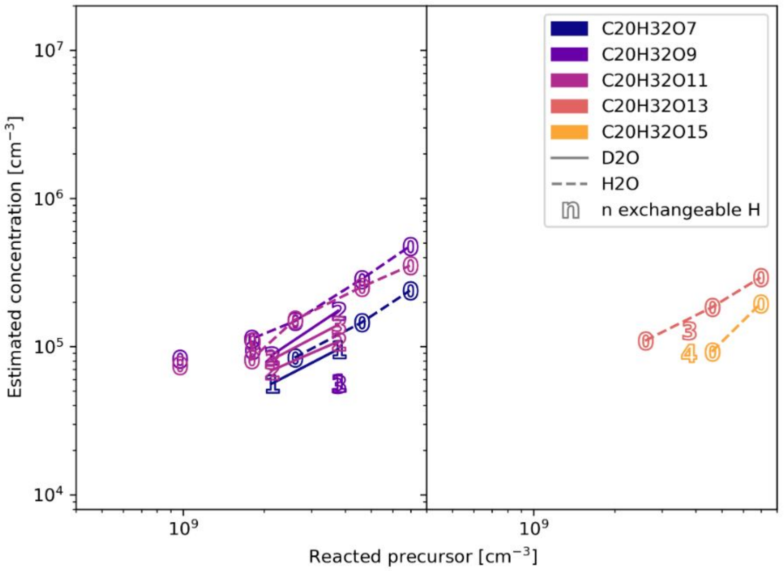

Fig. S17. Heavy water data

Estimated concentrations of α-pinene ozonolysis and OH oxidation products as a function of reacted α-pinene where the dashed line is H_2O experiment data and solid line is D_2O data, and the marker shows the number of exchangeable hydrogens in the product. (**A**) Products $C_{10}H_{15}O_{6,8,10}$ and $C_{10}H_{14}O_{5,7,9}$, (**B**) $C_{10}H_{15}O_{odd}$ and $C_{10}H_{17}O_{7,10}$, (**C**) $C_{10}H_{16}O_{4,5,7\text{-}10}$, (**D**) $C_{19}H_{28}O_{8,9,11}$ and $C_{20}H_{30}O_{10,12}$, and (**E**) $C_{20}H_{32}O_{odd}$.

S11. Oxidation product yield comparisons

Comparison of oxidation product yields between each selectively deuterated precursor and non-labelled α-pinene, where the used data have comparable precursor concentrations.

Figs. S18-25. Yields from deuterated precursors against α-pinene

Product yields of each deuterated precursor as a function of matching product yields of non-labelled α-pinene where the color shows (**A, B**) the number of O atoms in the compound, and (**C, D**) the average amount of D atoms lost per compound. The yields for nitrate data are shown on (A, C) and amine data on (B, D). The marker size decreases with decreasing number of oxygen atoms in the product. The products with less than 7 oxygen atoms are marked with red circles, and products $C_{10}H_{14}O_n$ are marked with a white star, $C_{10}H_{15}O_n$ with a white dot, and $C_{10}H_{17}O_n$ with a white cross.

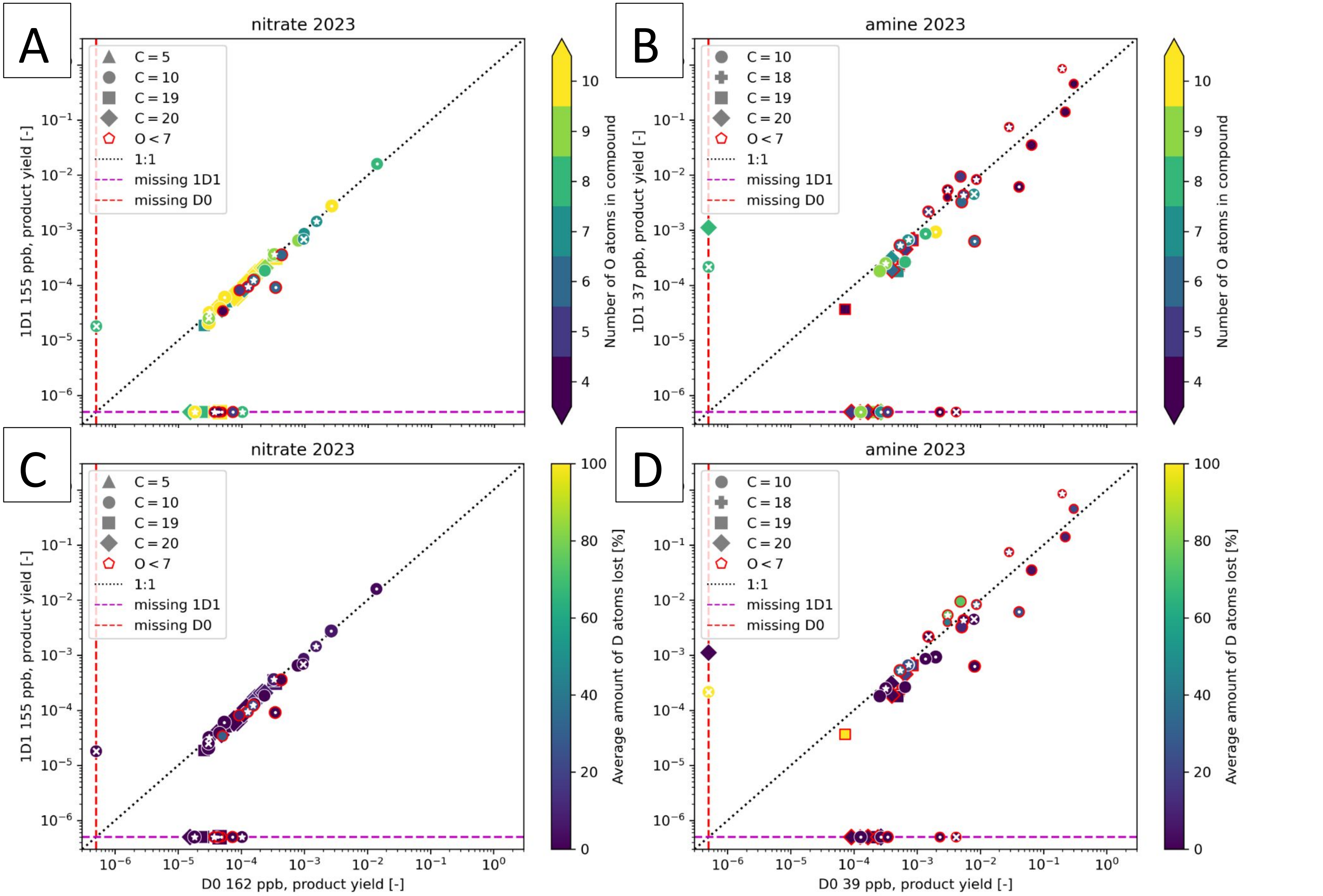

Fig. S18. Product yields of C1 deuterated precursor (1D1).

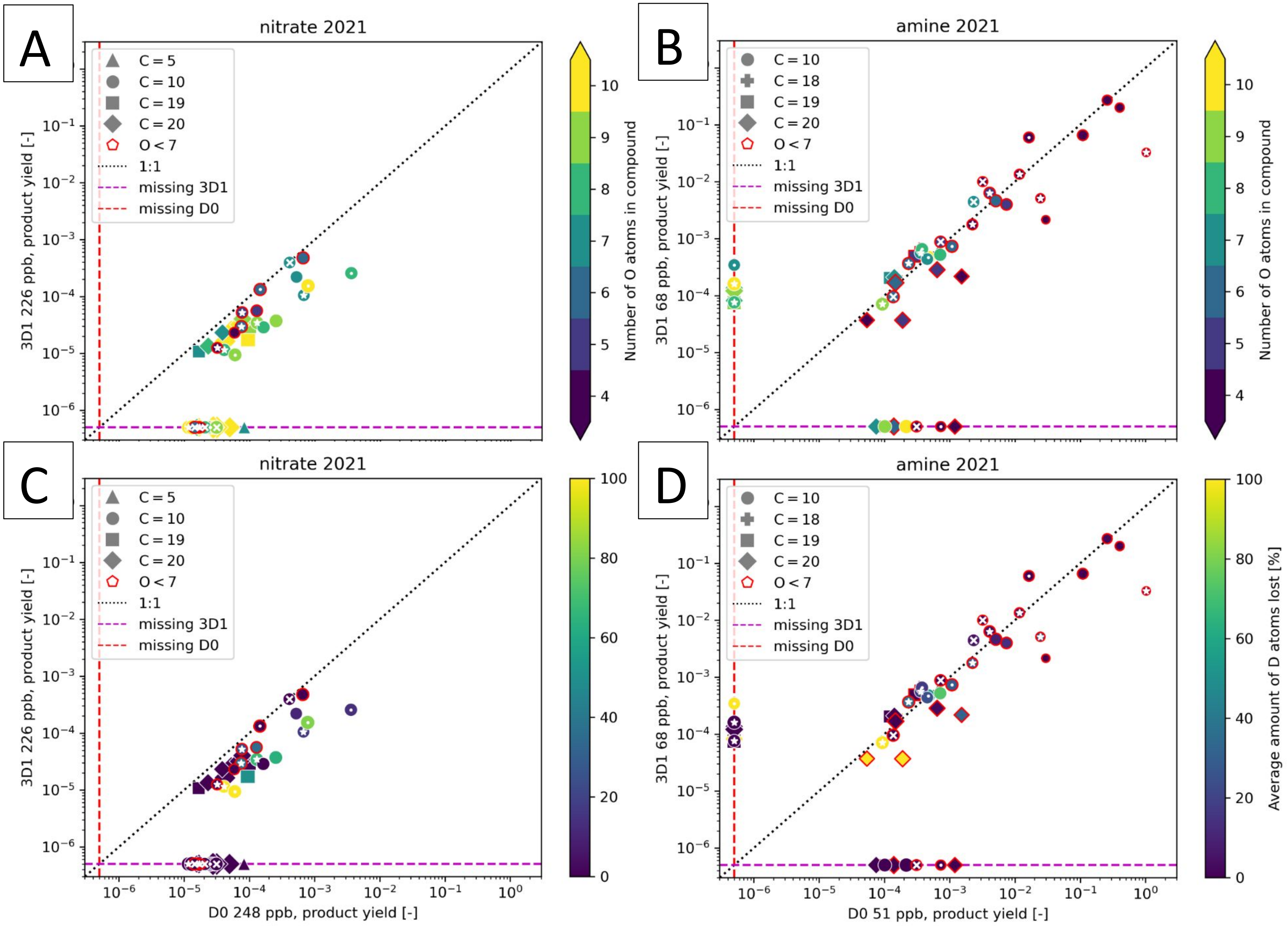

Fig. S19. Product yields of C3 deuterated precursor (3D1).

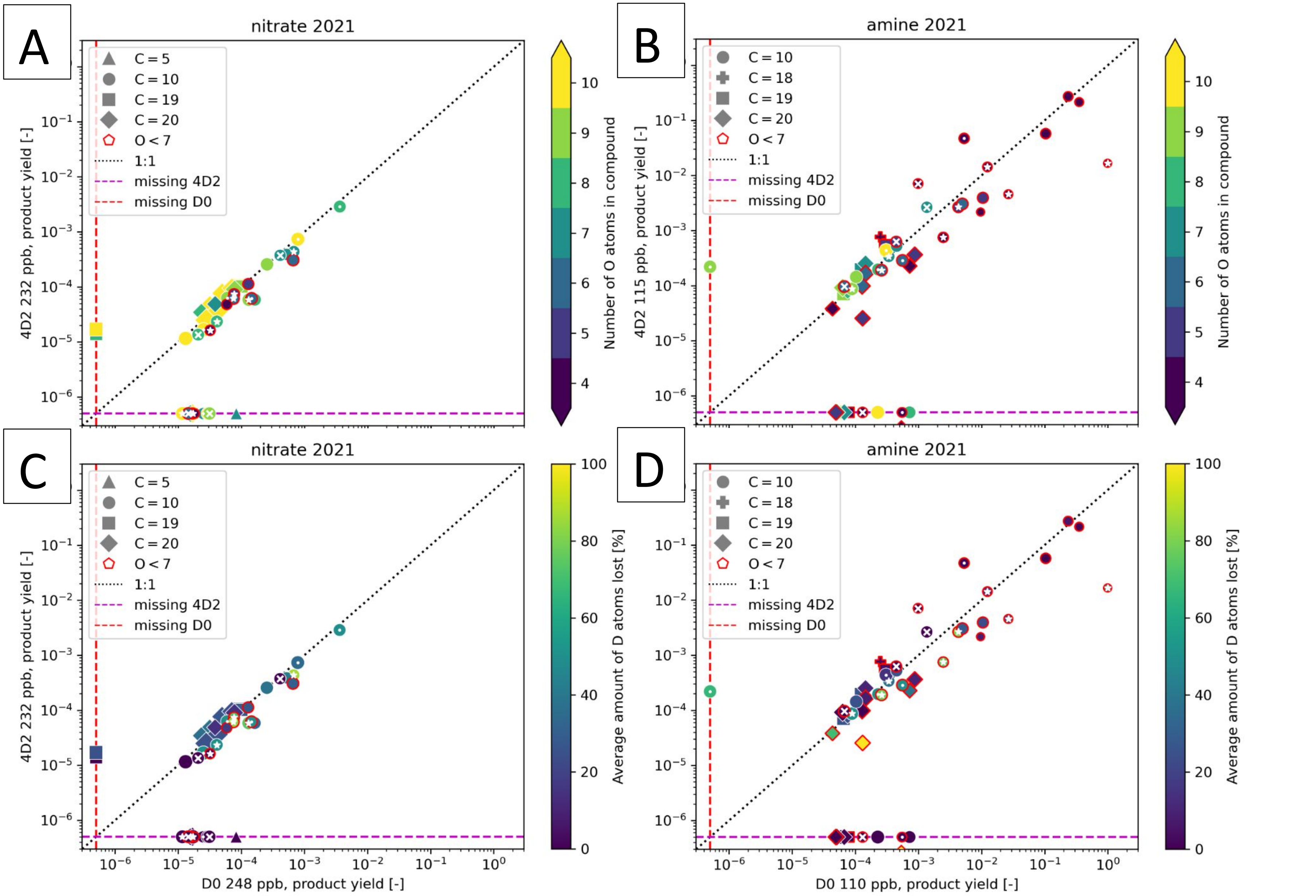

Fig. S20. Product yields of C4 deuterated precursor (4D2).

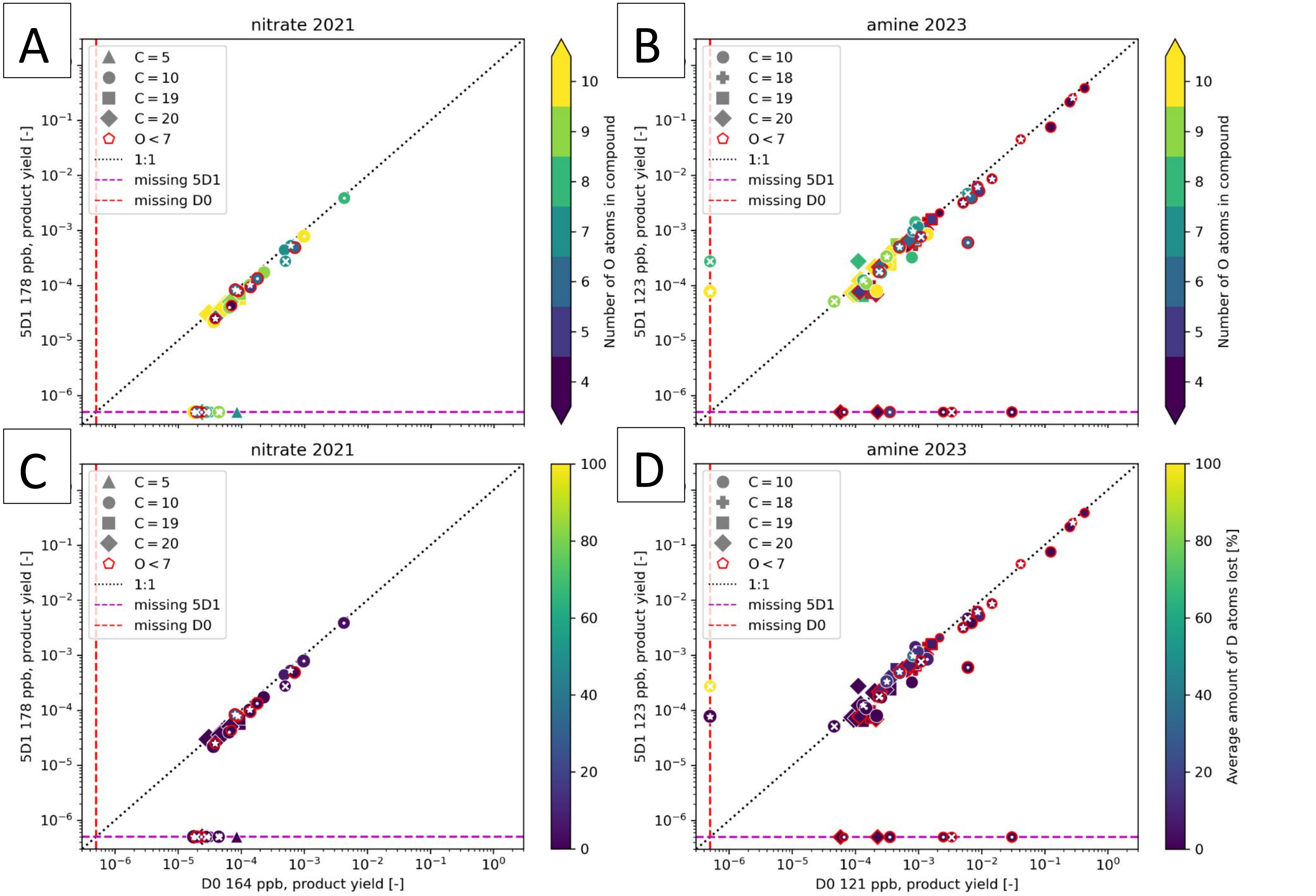

Fig. S21. Product yields of C5 deuterated precursor (5D1).

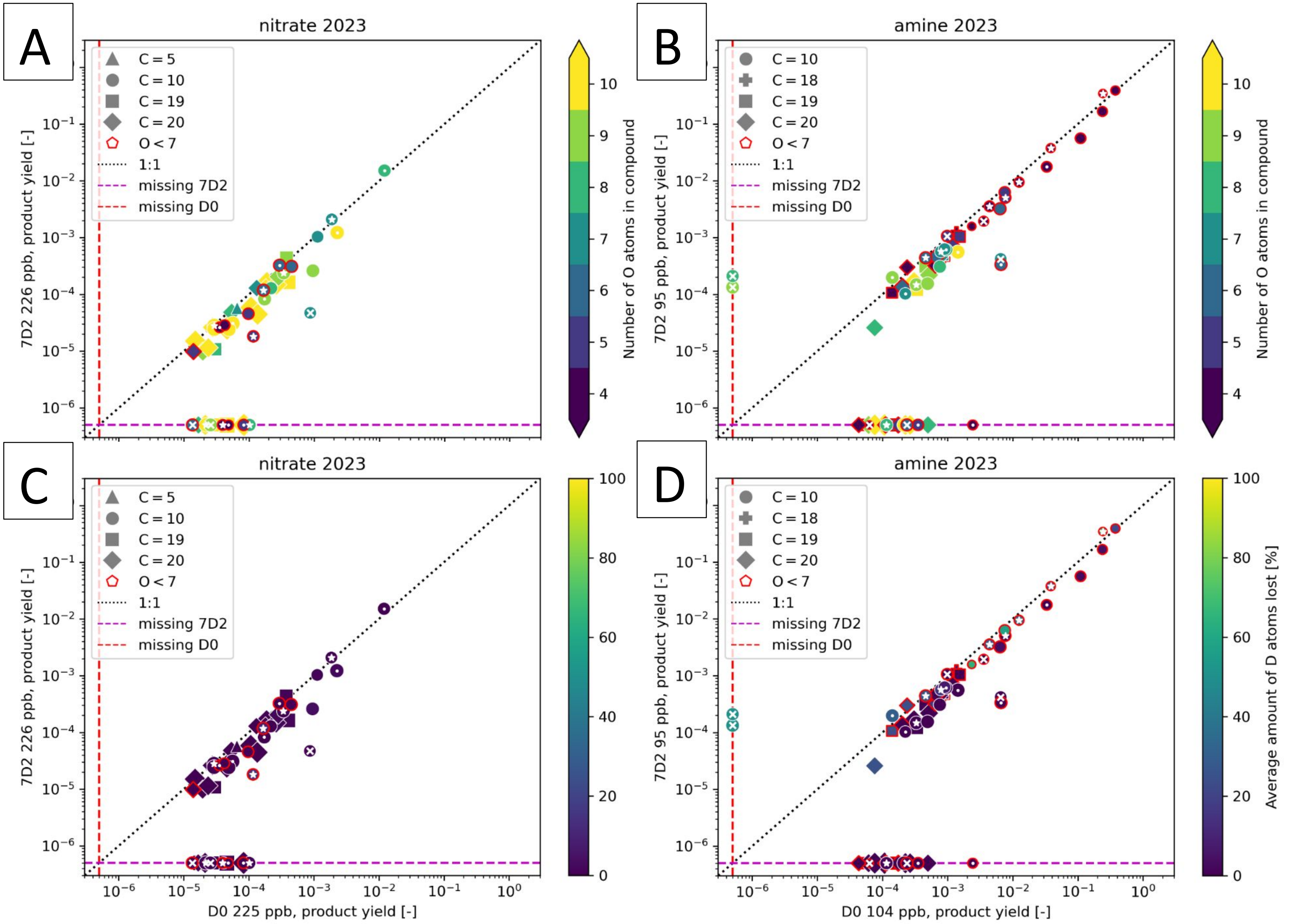

Fig. S22. Product yields of C7 deuterated precursor (7D2).

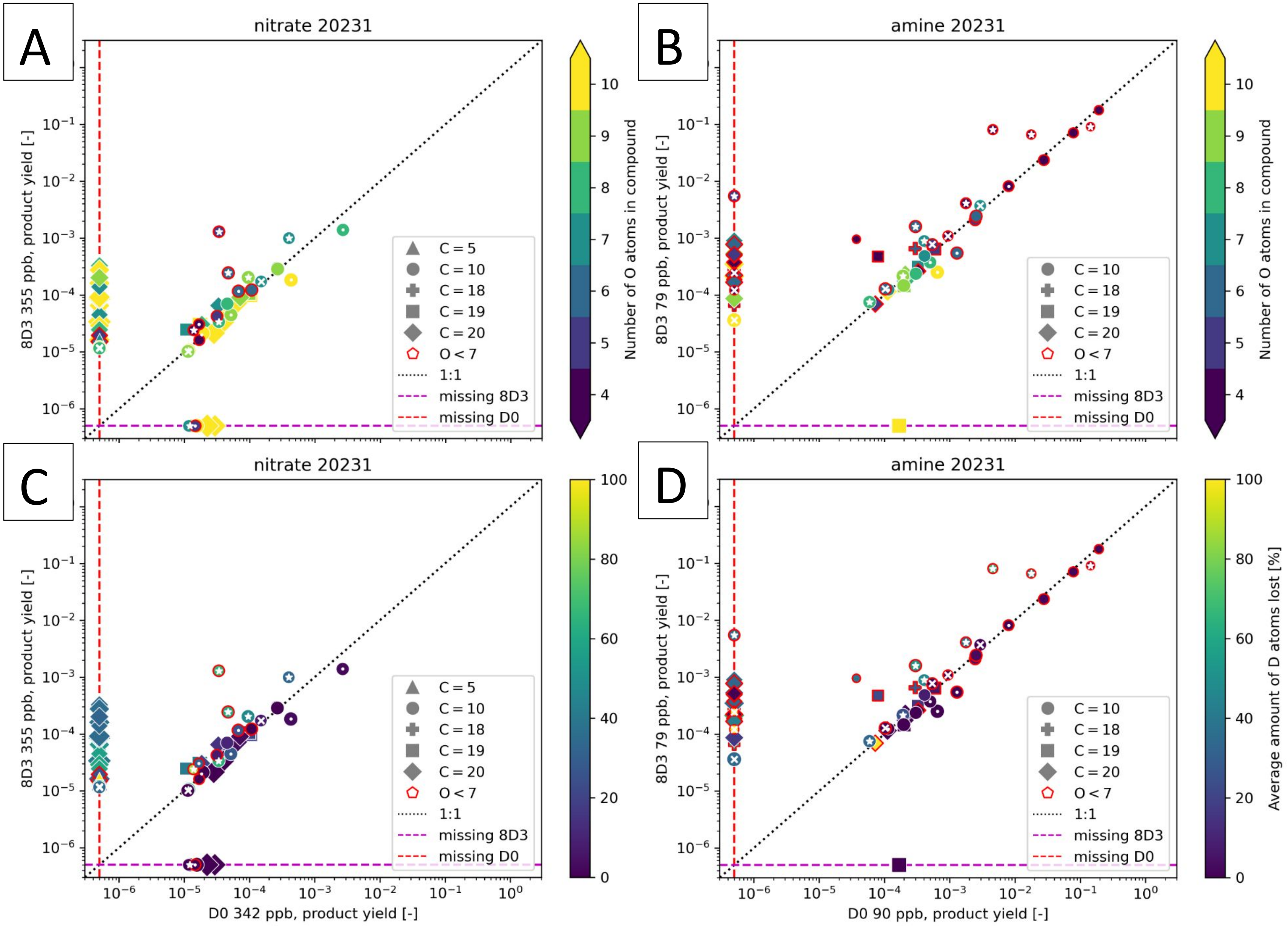

Fig. S23. Product yields of C8 deuterated precursor (8D3).

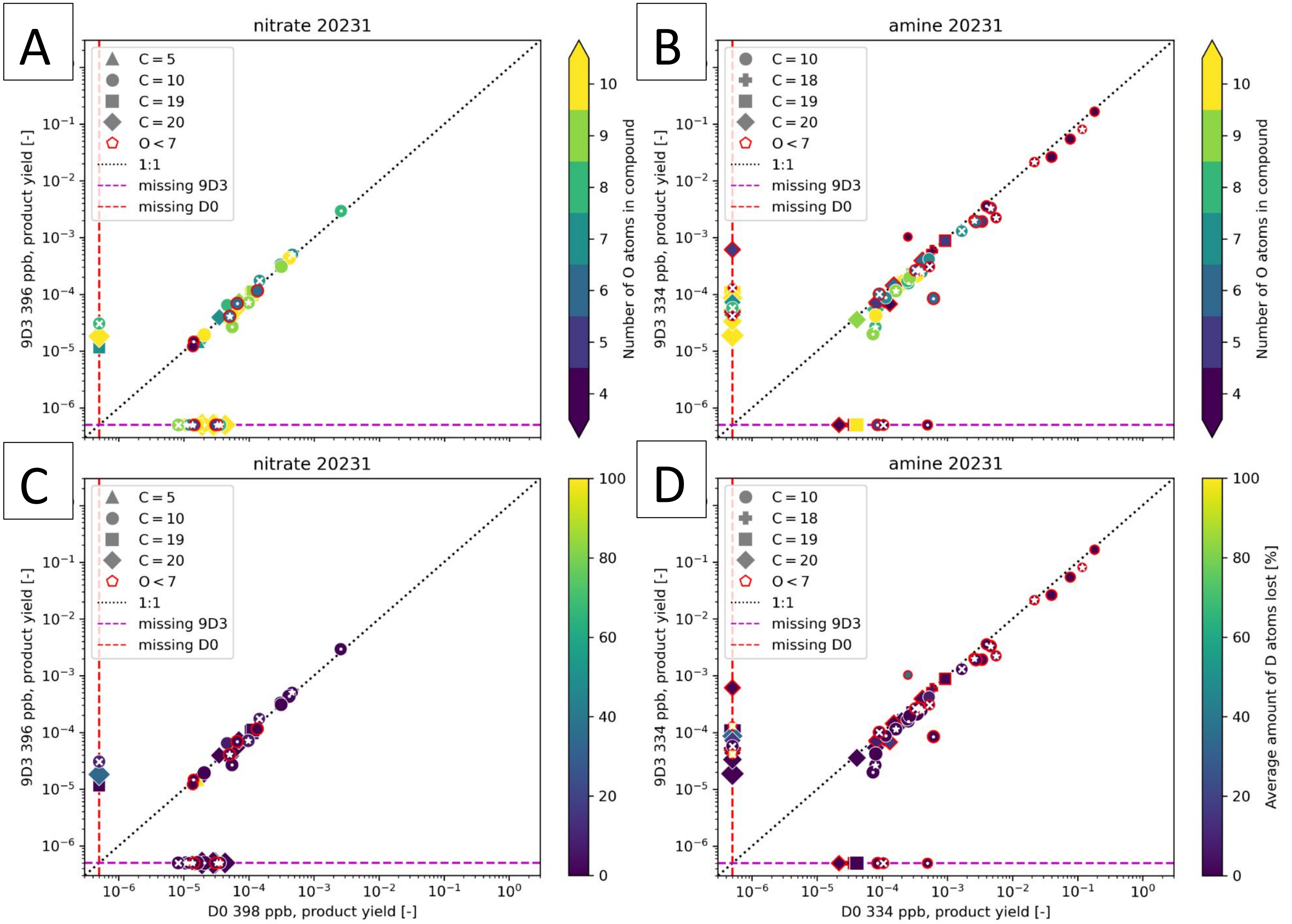

Fig. S24. Product yields of C9 deuterated precursor (9D3).

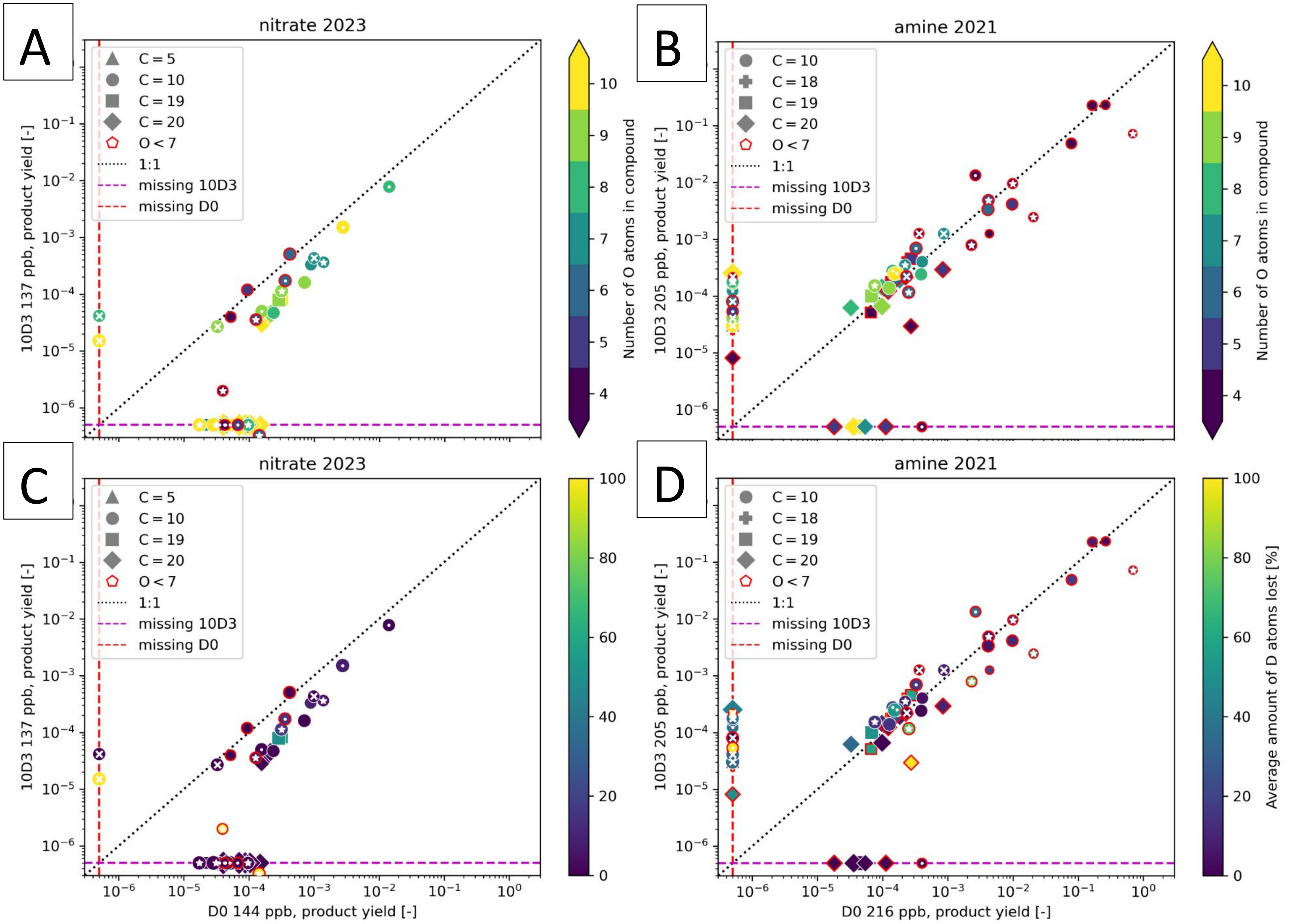

Fig. S25. Product yields of C10 deuterated precursor (10D3).

S12. Estimated yields for selectively deuterated precursor data

The HOM yields, i.e. yields of products with 7 or more oxygen atoms, as a function of precursor concentration in the flow tube when the data is acquired. And the yield of C10H16O3 from N-butylaminium data. The yields do not differ based on the used precursor when comparing the yields per the experiment year.

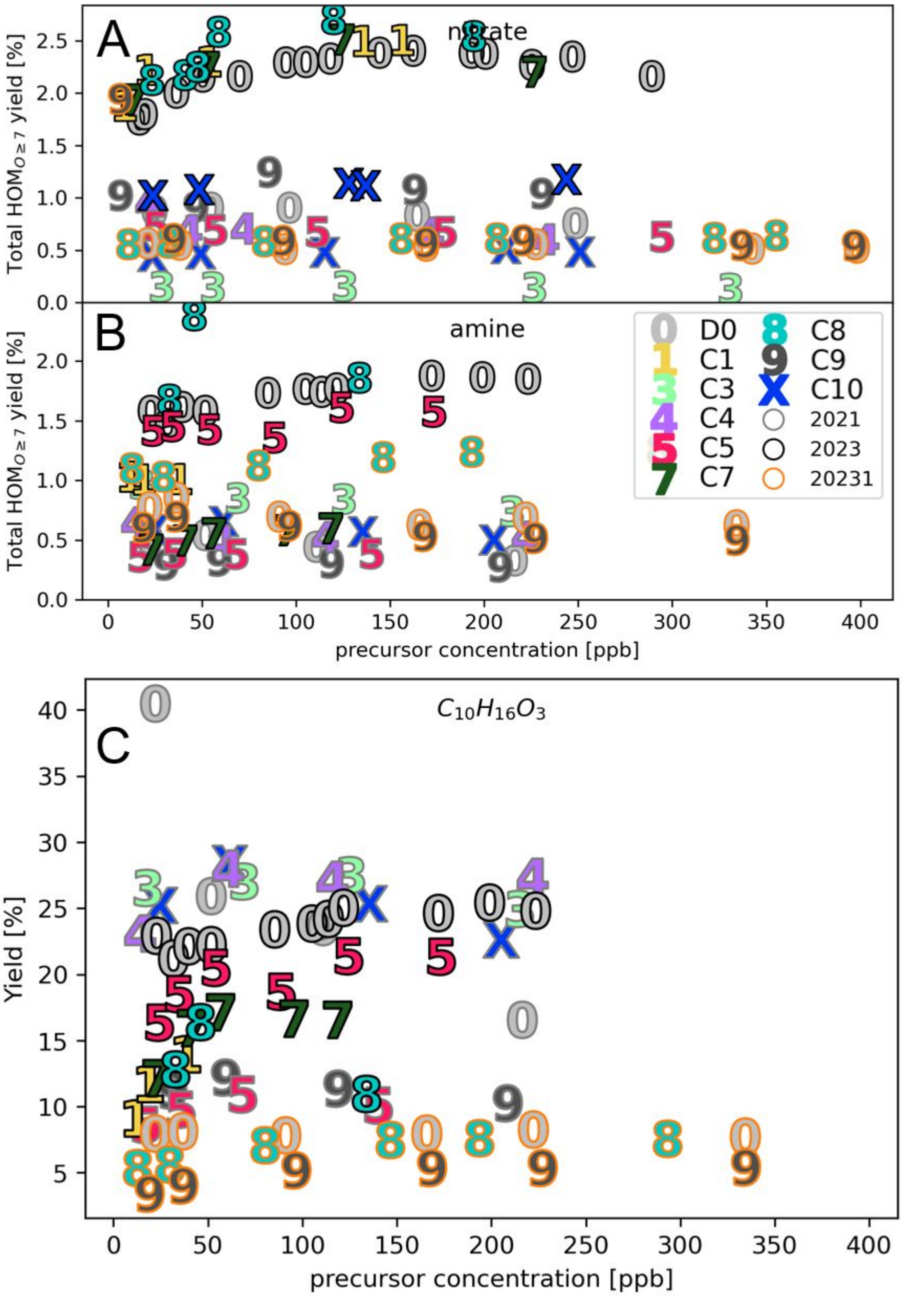

Fig. S26. Total estimated yields

Yields as a function of precursor concentration for all deuterated precursors. (**A, B**) Total yields of HOMs (7 or more oxygen atoms in molecule), (**C**) yields of $C_{10}H_{16}O_3$ (N-butylaminium data).

S13. Extended data

The extended data showing the top 20 yield products observed when using non-labelled α-pinene.

Table S2. Top 20 yield products

Top 20 highest yields of inspected compounds from D0 experiment in descending order for nitrate and aminium data separately at two reacted precursor concentrations, namely $8.2 \cdot 10^8$ cm^{-3} and $3.7 \cdot 10^9$ cm^{-3}. The lower concentration shows the yields when mostly unimolecular reactions take place, and the higher shows the yields when there is a significant number of bimolecular reactions.

**aminium 8.4e8 cm^{-3}**		**aminium 3.7e9 cm^{-3}**		**nitrate 8.2e8 cm^{-3}**		**nitrate 3.7e9 cm^{-3}**	
**compound**	**yield (-)**	**compound**	**yield (-)**	**compound**	**yield (-)**	**compound**	**yield (-)**
$C_{10}H_{16}O_2$	3.10e-1	$C_{10}H_{16}O_2$	4.86e-1	$C_{10}H_{15}O_8$	1.41e-2	$C_{10}H_{15}O_8$	1.20e-2
$C_{10}H_{16}O_3$	2.22e-1	$C_{10}H_{14}O$	2.87e-1	$C_{10}H_{15}O_{10}$	3.26e-3	$C_{10}H_{15}O_{10}$	2.26e-3
$C_{10}H_{14}O$	2.10e-1	$C_{10}H_{16}O_3$	2.49e-1	$C_{10}H_{17}O_7$	1.13e-3	$C_{10}H_{14}O_7$	1.86e-3
$C_{10}H_{16}O_4$	7.49e-2	$C_{10}H_{16}O_4$	1.31e-1	$C_{10}H_{14}O_7$	7.16e-4	$C_{10}H_{16}O_7$	1.13e-3
$C_{10}H_{15}O_4$	3.81e-2	$C_{10}H_{14}O_2$	4.68e-2	$C_{10}H_{16}O_7$	5.22e-4	$C_{10}H_{16}O_9$	9.55e-4
$C_{10}H_{14}O_2$	2.97e-2	$C_{10}H_{14}O_3$	1.84e-2	$C_{10}H_{15}O_6$	4.48e-4	$C_{10}H_{17}O_7$	8.69e-4
$C_{10}H_{14}O_3$	9.00e-3	$C_{10}H_{15}O_4$	1.77e-2	$C_{10}H_{16}O_6$	3.78e-4	$C_{10}H_{16}O_6$	4.50e-4
$C_{10}H_{15}O_6$	7.41e-3	$C_{10}H_{16}O_5$	1.41e-2	$C_{10}H_{16}O_9$	3.19e-4	$C_{19}H_{28}O_{11}$	4.00e-4
$C_{10}H_{17}O_7$	7.27e-3	$C_{10}H_{14}O_5$	1.20e-2	$C_{10}H_{14}O_9$	2.67e-4	$C_{19}H_{28}O_9$	3.74e-4
$C_{10}H_{14}O_5$	5.60e-3	$C_{10}H_{16}O_6$	8.54e-3	$C_{10}H_{16}O_8$	2.44e-4	$C_{10}H_{14}O_9$	3.40e-4
$C_{10}H_{16}O_6$	5.14e-3	$C_{10}H_{14}O_4$	7.03e-3	$C_{19}H_{28}O_{11}$	1.59e-4	$C_{10}H_{15}O_6$	2.94e-4
$C_{10}H_{16}O_5$	4.97e-3	$C_{10}H_{15}O_6$	4.35e-3	$C_{19}H_{28}O_9$	1.43e-4	$C_{20}H_{32}O_9$	2.85e-4
$C_{10}H_{17}O_3$	3.80e-3	$C_{10}H_{17}O_7$	4.23e-3	$C_{10}H_{14}O_5$	1.32e-4	$C_{20}H_{32}O_{11}$	2.64e-4
$C_{10}H_{14}O_4$	3.11e-3	$C_{10}H_{15}O_3$	2.45e-3	$C_{10}H_{14}O_6$	1.13e-4	$C_{10}H_{16}O_8$	2.17e-4
$C_{10}H_{16}O$	2.75e-3	$C_{19}H_{28}O_5$	2.29e-3	$C_{20}H_{32}O_9$	1.12e-4	$C_{20}H_{30}O_{10}$	1.88e-4
$C_{10}H_{15}O_3$	2.23e-3	$C_{20}H_{32}O_5$	2.05e-3	$C_{10}H_{14}O_6$	1.08e-4	$C_{20}H_{30}O_{12}$	1.80e-4
$C_{10}H_{15}O_{10}$	1.77e-3	$C_{18}H_{26}O_4$	1.57e-3	$C_{20}H_{32}O_{11}$	1.02e-4	$C_{10}H_{15}O_9$	1.73e-4
$C_{10}H_{17}O_5$	1.29e-3	$C_{10}H_{16}O$	1.55e-3	$C_{10}H_{15}O_9$	1.00e-4	$C_{10}H_{14}O_6$	1.67e-4
$C_{10}H_{15}O_8$	1.18e-3	$C_{19}H_{28}O_7$	1.24e-3	$C_{20}H_{30}O_{10}$	7.74e-5	$C_{20}H_{32}O_{13}$	1.35e-4
$C_{18}H_{26}O_4$	9.50e-4	$C_{10}H_{16}O_7$	1.21e-3	$C_{20}H_{32}O_{13}$	7.07e-5	$C_{20}H_{32}O_7$	1.31e-4

Supplementary references

(1) Meder, M.; Peräkylä, O.; Varelas, J. G.; Luo, J.; Cai, R.; Zhang, Y.; Kurtén, T.; Riva, M.; Rissanen, M.; Geiger, F. M.; Thomson, R. J.; Ehn, M. Selective Deuteration as a Tool for Resolving Autoxidation Mechanisms in *α* -Pinene Ozonolysis. *Atmos Chem Phys* 2023, *23* (7), 4373–4390. https://doi.org/10.5194/acp-23-4373-2023.

(2) Cai, R.; Li, Y.; Clément, Y.; Li, D.; Dubois, C.; Fabre, M.; Besson, L.; Perrier, S.; George, C.; Ehn, M.; Huang, C.; Yi, P.; Ma, Y.; Riva, M. Orbitool: A Software Tool for Analyzing Online Orbitrap Mass Spectrometry Data. *Atmos Meas Tech* 2021, *14* (3), 2377–2387. https://doi.org/10.5194/amt-14-2377-2021.

(3) Junninen, H.; Ehn, M.; Petäjä, T.; Luosujärvi, L.; Kotiaho, T.; Kostiainen, R.; Rohner, U.; Gonin, M.; Fuhrer, K.; Kulmala, M.; Worsnop, D. R. A High-Resolution Mass Spectrometer to Measure Atmospheric Ion Composition. *Atmos Meas Tech* 2010, *3* (4), 1039–1053. https://doi.org/10.5194/amt-3-1039-2010.

(4) Kurtén, T.; Rissanen, M. P.; Mackeprang, K.; Thornton, J. A.; Hyttinen, N.; Jørgensen, S.; Ehn, M.; Kjaergaard, H. G. Computational Study of Hydrogen Shifts and Ring-Opening Mechanisms in α-Pinene Ozonolysis Products. *J Phys Chem A* 2015, *119* (46), 11366–11375. https://doi.org/10.1021/acs.jpca.5b08948.

(5) Saunders, S. M.; Jenkin, M. E.; Derwent, R. G.; Pilling, M. J. Protocol for the Development of the Master Chemical Mechanism, MCM v3 (Part A): Tropospheric Degradation of Non-Aromatic Volatile Organic Compounds. *Atmos Chem Phys* 2003, *3* (1), 161–180. https://doi.org/10.5194/acp-3-161-2003.

(6) Iyer, S.; Rissanen, M. P.; Valiev, R.; Barua, S.; Krechmer, J. E.; Thornton, J.; Ehn, M.; Kurtén, T. Molecular Mechanism for Rapid Autoxidation in α-Pinene Ozonolysis. *Nat Commun* 2021, *12* (1), 878. https://doi.org/10.1038/s41467-021-21172-w.

(7) Crounse, J. D.; Nielsen, L. B.; Jørgensen, S.; Kjaergaard, H. G.; Wennberg, P. O. Autoxidation of Organic Compounds in the Atmosphere. *J Phys Chem Lett* 2013, *4* (20), 3513–3520. https://doi.org/10.1021/jz4019207.

(8) Iyer, S.; Reiman, H.; Møller, K. H.; Rissanen, M. P.; Kjaergaard, H. G.; Kurtén, T. Computational Investigation of RO 2 + HO 2 and RO 2 + RO 2 Reactions of Monoterpene Derived First-Generation Peroxy Radicals Leading to Radical Recycling. *J Phys Chem A* 2018, *122* (49), 9542–9552. https://doi.org/10.1021/acs.jpca.8b09241.

(9) Orlando, J. J.; Tyndall, G. S. Laboratory Studies of Organic Peroxy Radical Chemistry: An Overview with Emphasis on Recent Issues of Atmospheric Significance. *Chem Soc Rev* 2012, *41* (19), 6294. https://doi.org/10.1039/c2cs35166h.

(10) Peräkylä, O.; Berndt, T.; Franzon, L.; Hasan, G.; Meder, M.; Valiev, R. R.; Daub, C. D.; Varelas, J. G.; Geiger, F. M.; Thomson, R. J.; Rissanen, M.; Kurtén, T.; Ehn, M. Large Gas-Phase Source of Esters and Other Accretion Products in the Atmosphere. *J Am Chem Soc* 2023, *145* (14), 7780–7790. https://doi.org/10.1021/jacs.2c10398.

(11) Cremer, D.; Kraka, E.; Sclay, P. G. Decomposition Modes of Dioxirane, Methyldioxirane and Dimethyldioxirane — a CCSD T , MR-AQCC and DFT Investigation. *Chem Phys Lett* 1998, *292*, 97–109.

(12) Berndt, T.; Richters, S.; Kaethner, R.; Voigtländer, J.; Stratmann, F.; Sipilä, M.; Kulmala, M.; Herrmann, H. Gas-Phase Ozonolysis of Cycloalkenes: Formation of Highly Oxidized

RO 2 Radicals and Their Reactions with NO, NO 2 , SO 2 , and Other RO 2 Radicals. *J Phys Chem A* 2015, *119* (41), 10336–10348. https://doi.org/10.1021/acs.jpca.5b07295.
(13) Berndt, T.; Mentler, B.; Scholz, W.; Fischer, L.; Herrmann, H.; Kulmala, M.; Hansel, A. Accretion Product Formation from Ozonolysis and OH Radical Reaction of α-Pinene: Mechanistic Insight and the Influence of Isoprene and Ethylene. *Environ Sci Technol* 2018, *52* (19), 11069–11077. https://doi.org/10.1021/acs.est.8b02210.
(14) Riva, M.; Brüggemann, M.; Li, D.; Perrier, S.; George, C.; Herrmann, H.; Berndt, T. Capability of CI-Orbitrap for Gas-Phase Analysis in Atmospheric Chemistry: A Comparison with the CI-APi-TOF Technique. *Anal Chem* 2020, *92* (12), 8142–8150. https://doi.org/10.1021/acs.analchem.0c00111.
(15) Heinritzi, M.; Simon, M.; Steiner, G.; Wagner, A. C.; Kürten, A.; Hansel, A.; Curtius, J. Characterization of the Mass-Dependent Transmission Efficiency of a CIMS. *Atmos Meas Tech* 2016, *9* (4), 1449–1460. https://doi.org/10.5194/amt-9-1449-2016.
(16) Berndt, T. Peroxy Radical and Product Formation in the Gas-Phase Ozonolysis of α-Pinene under Near-Atmospheric Conditions: Occurrence of an Additional Series of Peroxy Radicals O,O–C 10 H 15 O(O 2) *y* O 2 with *y* = 1–3. *J Phys Chem A* 2022, *126* (37), 6526–6537. https://doi.org/10.1021/acs.jpca.2c05094.
(17) Atkinson, R.; Baulch, D. L.; Cox, R. A.; Crowley, J. N.; Hampson, R. F.; Hynes, R. G.; Jenkin, M. E.; Rossi, M. J.; Troe, J. Evaluated Kinetic and Photochemical Data for Atmospheric Chemistry: Volume II – Gas Phase Reactions of Organic Species. *Atmos Chem Phys* 2006, *6* (11), 3625–4055. https://doi.org/10.5194/acp-6-3625-2006.
(18) Rissanen, M. P.; Kurtén, T.; Sipilä, M.; Thornton, J. A.; Kausiala, O.; Garmash, O.; Kjaergaard, H. G.; Petäjä, T.; Worsnop, D. R.; Ehn, M.; Kulmala, M. Effects of Chemical Complexity on the Autoxidation Mechanisms of Endocyclic Alkene Ozonolysis Products: From Methylcyclohexenes toward Understanding α-Pinene. *J Phys Chem A* 2015, *119* (19), 4633–4650. https://doi.org/10.1021/jp510966g.
